# Anti-Fireblight Potential of Fabclavines, Synthesized by the Type Strains of *Xenorhabdus szentirmaii* and *Xenorhabdus budapestensis*

**DOI:** 10.3390/antibiotics15100961

**Published:** 2026-09-28

**Authors:** Zsófia Boros, András Fodor, János Kiss, Ferenc Olasz, Pál Tamás Szabó, Mária Hevesi, Eustachio Tarasco, László Makrai, Patrik Mag, Ákos Jerzsele, Tibor Vellai, Lajos Szabolcs Földes

**Affiliations:** 1 Department of Genetics, Eötvös Loránd University, Pázmány Péter Sétány 1/C, H-1117 Budapest, Hungary; boroszsofia@student.elte.hu (Z.B.); drfodorandras1@gmail.com or fodor.andras@ttk.elte.hu (A.F.); vellai.tibor@ttk.elte.hu (T.V.); 2 Doctoral School of Biology, Faculty of Science, Eötvös Loránd University, Pázmány Péter Sétány 1/C, H-1117 Budapest, Hungary; 3 Agribiotechnology and Precision Breeding for Food Security National Laboratory, Institute of Genetics and Biotechnology, Hungarian University of Agriculture and Life Sciences (MATE), H-2100 Gödöllő, Hungary; kiss.janos@uni-mate.hu (J.K.); olasz.ferenc.gyorgy@uni-mate.hu (F.O.); 4 MS Metabolomics Research Laboratory, Centre for Structural Science, HUN-REN Research Centre for Natural Sciences, Magyar Tudósok krt 2, H-1117 Budapest, Hungary; szabo.pal@ttk.hu; 5 Department of Fruit Growing, Institute of Horticultural Sciences, Hungarian University of Agriculture and Life Sciences (MATE), Villányi u. 29-43, H-1118 Budapest, Hungary; hevesilaszl13@gmail.com; 6 Department of Soil, Plant and Food Sciences, University of Bari “Aldo Moro”, 70121 Bari, Italy; eustachio.tarasco@uniba.it; 7 Autovakcina Kft., H-1171 Budapest, Hungary; info@autovakcina.hu; 8 Department of Pharmacology and Toxicology, University of Veterinary Medicine Budapest, H-1078 Budapest, Hungary; mag.patrik@univet.hu (P.M.); jerzsele.akos@univet.hu (Á.J.); 9 National Laboratory of Infectious Animal Diseases, Antimicrobial Resistance, Veterinary Public Health and Food Chain Safety, University of Veterinary Medicine Budapest, H-1078 Budapest, Hungary; 10 HUN-REN-ELTE Genetics Research Group, Eötvös Loránd University, H-1117 Budapest, Hungary; 11 Plant Health Department, Division of Agriculture, Agricultural Office of Szabolcs-Szatmár-Bereg County, H-4400 Nyíregyháza, Hungary

**Keywords:** *Erwinia amylovora*, antimicrobial peptides, easy PACId, *Xenorhabdus*, plant protection, biological control

## Abstract

The options for controlling agricultural plant-pathogenic bacterial diseases with antibiotics are rather limited. Applications of naturally derived antimicrobial peptides may offer an alternative. **Objectives.** The cell-free conditioned media of *Xenorhabdus budapestensis* and *X. szentirmaii* bacteria inactivate *Erwinia amylovora*, the causative agent of fire blight in Rosaceae, both in vitro and in planta. The working hypothesis was that the active ingredient was fabclavine. To test this hypothesis, inducible fabclavine-producing strains were constructed in both *Xenorhabdus* species. The anti-*Erwinia* potential of non-induced and induced fabclavine-producing cultures was compared in vitro and in planta. **Methods.** Promoter exchange mutants known to exclusively produce the fabclavine antibiotic were generated and their fabclavine production was confirmed by LC-HRMS, and in vitro bioassays. Finally, apple blossoms in an incubator were subjected to antimicrobial and control treatments before experimental inoculation of the buds with *Erwinia amylovora* strain Ea1. **Results.** Cell-free medium from both induced fabclavine-producing *Xenorhabdus* species exerted strong antagonistic effects on Ea1 cells in vitro, whereas the non-induced ones showed substantially reduced antibacterial activity. Similarly, both induced culture media prevented fire blight symptoms in experimentally inoculated flowers in a dose-dependent manner. The non-induced *X. szentirmaii* mutant strain was completely inactive in planta, whereas the *X. budapestensis* strain exhibited some protective activity. **Conclusions.** The results support the hypothesis that the fermentation product of the inducible *Xenorhabdus* mutants producing only fabclavines can be used for protection against *E. amylovora*. Some aspects of the application perspectives are discussed in comparison with conventional streptomycin and kasugamycin treatments.

## 1. Introduction

Fire blight is a devastating bacterial plant disease that attacks fruit orchards and severely impacts global apple and pear production. The causative agent of fire blight, the Gram-negative plant pathogen *Erwinia amylovora*, is one of the most important pome fruit pathogens worldwide [1,2,3]. The areas where the distribution of *E. amylovora* overlaps its host production area are considerable on all continents and are expected to expand further under future climatic conditions [3]; however, the severe economic losses caused by *E. amylovora* could be reduced by half by implementing appropriate control measures [3].

Fire blight is one of the most difficult plant diseases to control, because *E. amylovora* has an extensive host range within the Rosaceae family (*Pyracantha*, *Cotoneaster*, *Crataegus*, *Sorbus*) which often serve as hidden foci of infection, as was also confirmed in Hungary, where fire blight appeared in 1995 [4].

Among the limited number of control options currently available, prophylactic application of antibiotics during the bloom period still seems the most effective [5]. Historically, antibiotics have been used in plant medicine since the 1950s to control certain bacterial diseases in high-value fruit, vegetable, and ornamental plants without reports of adverse effects on human health or persistent impacts on the environment [6]. Springtime antibiotic sprays suppressed pathogen growth on flowers and leaf surfaces before infection; however, the use of antibiotics after infection was ineffective [7].

Streptomycin has generally been used in many countries, while the use of oxytetracycline, oxolinic acid, and gentamicin is limited to only a few countries [7]. Antibiotics are applied only during periods of high disease risk; consequently, most orchards are not treated annually [7]. Extensive use of streptomycin to control fire blight has led to natural selection for streptomycin-resistant strains all over the world, and, as alternatives, oxytetracycline and kasugamycin have been permitted to be used in the USA against fire blight since 1974 and 2014, respectively, despite the appearance of oxytetracycline-resistant *E. amylovora* strains in California in 2018 [8].

As for kasugamycin resistance [9,10,11], although resistant *E. amylovora* strains have not yet been isolated from natural habitats, kasugamycin-resistant *E. amylovora* mutants could be generated by chemical mutagenesis [12]. Furthermore, a gene encoding the novel acetyltransferase, AAC(2′)-IIa, responsible for enzymatic inactivation of kasugamycin, appeared in a possibly transferable genomic island IncP of the rice-pathogen bacteria *Burkholderia glumae* and *Acidovorax avenae* [13]. All of these suggest that kasugamycin resistance can also easily emerge in nature, and it has spread among these plant pathogens through horizontal gene transfer [14]. Despite concerns regarding the development of resistance and environmental impacts [2], antibiotics—including streptomycin, oxytetracycline and kasugamycin—are still widely used to combat economically important bacterial plant diseases [15], unlike in EU countries such as Hungary, where the use of antibiotics for crop protection in open fields is not allowed.

The concerns about antibiotic resistance problems justify the need for novel antimicrobial agents with novel modes of action. Among non-antibiotic control methods, phage-based biocontrol [16], also called phage therapy [17,18], is an emerging method for managing plant pathogens. Phage therapy has recently become increasingly popular as an approach that avoids the unintended selection of life-threatening multidrug-resistant pathogenic bacteria and addresses the growing awareness of the transfer of resistance genes between pathogens [19]. Technologies for using broad host-range lytic bacteriophages, like “Keyvirus” [20], for disease management have recently been developed [16,21,22,23]. The advantages of phage therapy include its minimal impact on microbial community equilibrium, the lack of detrimental impacts on plants and beneficial microorganisms, and its capacity to eradicate drug-resistant bacteria [21]. In Korea, a phage cocktail in combination with kasugamycin was suggested as a potential treatment for fire blight [24]. Phage resistance, however, is a crucial challenge that should be overcome to enhance the effectiveness of phage therapy.

Antimicrobial peptides (AMPs) seem to be another promising choice. AMPs [25,26,27,28,29,30,31,32,33], preferably those produced by microbes as natural antagonistic compounds like pantocine [34] or RejuAgro A [2] are amongst the candidates for the control of fire blight. Several AMPs have recently been tested for anti-fire blight potential, including those produced by different *Pantoea* species. Several antimicrobial peptides comprise a group of polyamide (or modified-backbone biopolymer) molecules and their derivatives which can be synthesized using modern chemical peptide synthesis methods [35]. However, AMPs are also produced in all taxa of the living world [26,33], and usually play a role as inherent components of the innate immune system in self-defense mechanisms [26], either directly or through immunomodulatory activity [27]. Consequently, AMPs represent a promising alternative to antibiotics in the face of the threatening post-antibiotic era and currently offer an important option for combating extreme multidrug-resistant pathogens [29].

Bacteria that exhibit resistance to an antibiotic often express another (pleiotropic) phenotype simultaneously, which involves susceptibility to other unrelated drugs (collateral sensitivity [30]). A recent discovery indicates that antibiotic-resistant [30,31] and multiresistant bacteria frequently show collateral sensitivity towards AMPs [31]. When studying the susceptibilities of a comprehensive set of 60 antibiotic-resistant *Escherichia coli* strains towards 24 AMPs, it was found that antibiotic-resistant bacteria frequently show collateral sensitivity to these peptides, whereas cross-resistance was relatively rare [31]. Furthermore, it turned out that AMP resistance genes and antibiotic resistance genes differ in their mobilization patterns and functional compatibilities with new bacterial hosts [32]. Chemical-genetic profiling also shows that only limited cross-resistance is observed against AMPs with different modes of action [33].

The practical goal of this project is to contribute to the international efforts to control fire blight, the most significant global threat to commercial fruits (apples and pears [14] and, in some countries—like Hungary—plum [36]). As part of the ongoing search for novel antimicrobial compounds effective against fire blight, attention has focused on the antibacterial potential of fabclavine and the fabclavine-producing bacteria.

AMPs produced by *Xenorhabdus budapestensis* DSM16342 (EMA) and *X. szentirmaii* DSM338 (EMC) [37,38] have previously been reported to exhibit antibacterial activity against several plant pathogenic bacteria, including *E. amylovora* [39,40]. To specifically evaluate the contribution of fabclavines to the antibacterial activity of EMA and EMC, genetically engineered strains with altered antimicrobial peptide production were generated. In these mutants, the biosynthesis of most antimicrobial secondary metabolites was silenced by the deletion of the *hfq* gene [41], while fabclavine production was selectively restored under the control of an L-arabinose-inducible promoter. Cell-free culture media (CFCMs) obtained from the wild-type and mutant strains were subsequently evaluated against *E. amylovora*.

Given the increasing concern over antibiotic resistance and the restrictions associated with the agricultural use of conventional antibiotics, the antibacterial activity of EMA- and EMC-derived CFCMs against some multidrug-resistant bacteria was evaluated.

## 2. Results

### 2.1. Generation of Inducible Fabclavine-Producing Xenorhabdus Strains by Promoter Exchange

To investigate and compare the antibacterial effects of fabclavines (and fabclavine biosynthetic precursors) produced by the type strains of *X. budapetsneis* (EMA) and *X. szentirmaii* (EMC), mutant strains were generated in which only the fabclavine biosynthetic gene cluster (BGC) responsible for fabclavine production is inducibly active, while the other antimicrobial-producing BGCs are silent. Previously, *hfq*-deleted (Δ*hfq*) mutants were created in both *Xenorhabdus* species [42], in which the *hfq*-regulated BGCs, including *fcl*, were turned off [41]. Using these newly constructed Δ*hfq* mutants, L-arabinose-inducible fabclavine-producing knock-in mutant strains (*fcl* KI strains) were generated by exchanging the native promoter of the *fcl* BGC to the P*ara_BAD_* promoter according to the easyPACId (easy Promoter Activated Compound Identification) technique [41,43] (Figure 1).

Two kanamycin-resistant (Km^R^) promoter exchange vectors, pBZS29 and pBZS31 (Supplementary Table S1), were developed and mobilized from the *E. coli* S17-1 *λpir* strain [44], which is streptomycin-resistant (Sm^R^), into the EMC and EMA Δ*hfq* mutants respectively, which are ampicillin-resistant (Ap^R^), for the promoter exchange by homologous recombination. Several Km^R^Ap^R^Sm^S^ (resistant to kanamycin and ampicillin but sensitive to streptomycin) transconjugant colonies were further analyzed by colony PCRs specific to the Δ*hfq* alleles of both *Xenorhabdus* strains [42], and for the vector–chromosome junction formed at the first gene of the *fcl* BGC via homologous recombination between the homology of the promoter exchange vectors and the chromosome (Figure 1). The amplicons obtained from vector–chromosome junctions, including the fusion of the P*ara_BAD_* promoter and the first gene of the *fcl* BGC, were then sequenced, which confirmed that the *fcl* operon was placed under the L-arabinose-inducible promoter in the resulting knock-in (KI) mutants of both the EMA and EMC Δ*hfq* strains (Supplementary Figure S1 and sequence files in Supplementary Materials S1 and S2). These KI-mutant strains were then used to produce CFCMs under inducing and non-inducing conditions, and these CFCMs were applied in the following LC-MS measurements and bioassays to analyze their antibacterial potential against several selected species.

### 2.2. LC-MS Analysis of Induced and Non-Induced CFCMs of the EMA and EMC fcl KI Double Mutant Xenorhabdus Strains

LC-HRMS-based targeted screening measurements were performed on CFCM samples from induced and non-induced EMA and EMC *fcl* KI cultures. The screening strategy was based on the panel of 32 target compounds previously identified [45] (Table 1). Inspection of the molecular structures revealed that these compounds contain multiple nitrogen atoms, which are readily protonated under LC-MS conditions and therefore exhibit excellent ionization efficiency in positive electrospray ionization mode. Consequently, multiply charged ions were expected to dominate the mass spectra. As anticipated, the detected target compounds exhibited multiple positively charged ions. Accordingly, the targeted screening workflow included all five charge states (+1 to +5) for each of the 32 target compounds.

Accordingly, the targeted screening workflow was designed to include the extraction of all five charge states for each target compound. Extracted ion chromatograms (XICs) corresponding to these ions were generated for every analyte in each sample. A compound was considered positively identified only when at least two charge states were detected at the same retention time, providing strong evidence for the presence of the respective compound.

The same screening procedure was applied to the non-induced samples. As expected, no significant peaks corresponding to the target compounds were detected in these controls. The extracted ion chromatograms of the positively identified target compounds are shown in Figure 2 and Figure 3 for EMA and EMC, respectively. Owing to the high number of basic nitrogen atoms, the target compounds are predominantly protonated under acidic chromatographic conditions, resulting in reduced retention on the reversed-phase stationary phase. Since the high resolving power of the mass spectrometer enables the selective visualization of individual ions by XICs, baseline chromatographic separation of the target compounds was not considered necessary.

For several target compounds, multiple peaks were observed in the XICs. Detailed examination of the corresponding high-resolution mass spectra demonstrated that these peaks were not attributable to chromatographically separated isomers. Instead, they originated from overlapping isotope patterns of compounds with different elemental compositions and closely related molecular masses, typically differing in mass by only 1–2 Da.

Based on the targeted screening results, compounds 1, 2, 3, 4, 6, 7, 8, 17, 18, 19, 22, 23, 25, 27, 29, and 30 were unequivocally identified in the CFCM of induced EMA *fcl* KI mutant (sample #1), with compounds 1–8 showing the highest signal intensities. In contrast, the CFCM of induced EMC *fcl* KI mutant (sample #3) was characterized by the predominant presence of compounds 15–21, whereas the lower-numbered target compounds were not detected. The extracted ion chromatograms and high-resolution mass spectra supporting these identifications are provided in the Supplementary Material S3.

### 2.3. Analyses of Induced and Non-Induced CFCMs Using the Agar Well Diffusion Assay

The antibacterial activity of CFCMs obtained from wild-type, Δ*hfq* mutant, and induced and non-induced Δ*hfq*-*fcl* KI double mutant EMA and EMC strains was assessed against *Erwinia amylovora* Ea1 [4], extended-spectrum β-lactamase-producing *Escherichia coli*, *Pseudomonas aeruginosa*, methicillin-resistant *Staphylococcus pseudintermedius* and *Enterococcus faecium* (ATCC6057) strains using the agar well diffusion assay (Figure 4 and Figure 5, see also Supplementary Figure S2).

The studied *P. aeruginosa* strain was not inhibited by any of the tested CFCMs. In contrast, the CFCMs from the wild-type and induced *fcl* KI strains exhibited antibacterial activity against all other tested bacteria (Table 2). No residual antibacterial activity could be detected in the non-induced (negative control) EMA *fcl* KI CFCM against any of the tested bacteria. In contrast, the non-induced EMC *fcl* KI CFCM formed a faint, barely measurable inhibition zone against *E. amylovora* Ea1, whereas no residual activity was observed against the other bacteria tested. The *Δhfq* mutants of EMA and EMC showed reduced antibacterial activity compared to the corresponding wild-type and induced *fcl* KI strains. The EMA *Δhfq* CFCM retained weak residual activity against the Gram-negative (*E. coli)* and the Gram-positive (*S. pseudintermedius)* control strains as well as Ea1, producing inhibition zones that were considerably smaller than those of the corresponding wild-type and induced *fcl* KI strains, whereas no antibacterial activity was detected for the EMC *Δhfq* mutant strain. These findings suggest that antibacterial activity of the two *Xenorhabdus* species considerably depends on their fabclavine production.

### 2.4. Results of in Planta Bioassay (Flower Tests)

To assess the potential plant protection application of CFCMs obtained from the EMA and EMC *fcl* KI strains against fire blight, an in planta bioassay was performed on Idared apple flowers. Induced and non-induced EMA and EMC *fcl* KI CFCMs were applied as a preventive treatment at various dilutions, while two commercial agricultural antibiotic formulations, kasugamycin-based Kasumin 2 L (Hokko Chemical, Tokyo, Japan) and streptomycin-based Streptomycin 20 WP (Meiji Seika Kaisha, Tokyo, Japan), were used as positive controls. Seven days after inoculation with *E. amylovora* Ea1, disease severity was assessed in all flowers (n = 30 per treatment) using the rating scale described by [46] (Figure 6 and Supplementary Table S2). Based on this rating scale, infection frequency (Figure 7), severity of infection (Figure 8), and the infection index (Figure 9) were determined using the following formulas:

$$Frequency\;of\;infection\left(I_{fq}\right)=\frac{Number\;of\;infected\;flowers}{Total\;number\;of\;flowers}\times 100$$

$$Severity\;of\;infection\;(S_{i})=\frac{Number\;of\;infected\;flowers\;x\;scale\;value}{Total\;number\;of\;flowers\;x\;maximal\;scale\;value}\times 100$$

$$Infection\;index=\frac{Total\;number\;of\;flowers\;x\;scale\;value}{Total\;number\;of\;flowers}$$

Although it was not systematically studied, no sign of any spectacular damage in the CFCM-treated flowers was observed during the experiments.

As expected, all of the untreated flowers inoculated with *E. amylovora* Ea1 (control) became infected and exhibited symptoms of fire blight (Figure 7). CFCMs derived from the induced *fcl* KI mutants of EMA and EMC provided efficient protection: up to 80% of the flowers remained uninfected and showed no symptoms of fire blight. In contrast, the CFCM from the uninduced *fcl* KI mutant EMC strain did not protect the flowers from *E. amylovora* infection. Unexpectedly, the pretreatment of the flowers with CFCM derived from the non-induced *fcl* KI mutant EMA strain also reduced the frequency of infection significantly in a dose-dependent manner, although to a smaller extent. This indicates the presence of other components in the CFCM of EMA*,* with bactericidal or bacteriostatic activity, whose biosynthesis is not *hfq*-controlled. Among the positive controls, Streptomycin 20 WP provided the highest level of protection, while Kasumin 2 L also markedly reduced the incidence of infection compared to the untreated control.

A similar trend was observed for infection severity (Figure 8). The highest infection severity values were recorded for the untreated control and the non-induced *fcl* KI mutant EMC strain, whereas the induction substantially reduced symptom development in case of treatment with both *fcl* KI *Xenorhabdus* strains. Similar to the infection frequency results, the non-induced EMA CFCM also reduced disease severity in a dose-dependent manner compared to the untreated control, although the induced EMA CFCM provided better results. Streptomycin 20 WP resulted in the lowest infection severity values, while Kasumin 2 L also considerably reduced symptom development. Treatment with the induced EMA and EMC CFCMs resulted in symptom severity comparable to that observed after Streptomycin 20 WP treatment.

The combined effects on infection frequency and disease severity are reflected by the infection index (Figure 9). Consistent with the previous parameters, the untreated control and the treatment with CFCM of the non-induced *fcl* KI EMC strain exhibited the highest infection index values at all concentrations tested, while treatment with induced *fcl* KI CFCMs of both *Xenorhabdus* strains markedly reduced the infection index. The CFCM of the non-induced *fcl* KI EMA lowered the infection index compared to the untreated control in a concentration-dependent manner, while induction further improved its efficacy. Among the positive controls, Streptomycin 20 WP showed the strongest protective effect, whereas Kasumin 2 L also substantially reduced the infection index.

In summary: the highest concentrations of CFCMs derived from induced cultures of EMA and EMC *fcl* KI mutants resulted in a significant reduction in the incidence of infection and the severity of symptoms, comparable to the effect observed after Streptomycin 20 WP treatment, demonstrating a strong protective effect against fire blight.

## 3. Discussion

Entomopathogenic nematode-symbiotic bacterium (EPB) species are rich sources of potent antibiotic compounds with a broad target spectrum [47,48,49,50]. The symbiotic associations of EPBs and their entomopathogenic nematode hosts (EPNs) are spectacular examples of coevolution. Every EPN/EPB symbiotic complex is a co-evolved ecosystem, where the EPB provides numerous different molecules that protect the insect’s carcass from competitive microorganisms. This is the explanation of the availability of so many valuable drug candidate molecules. The majority of them, like fabclavines, are of peptide nature. The production of this diverse array of bioactive compounds generally relies on the sophisticated regulatory system mediated by *hfq* [49,50].

This study aims to utilize the fabclavine [51] producing potential of two EPB strains, the type strains of *X. budapestensis* (EMA) and *X. szentirmaii* (EMC) species [37,38], which were previously found to be efficient producers of antimicrobials in controlling fire blight [39]. These globally available EPB species comprise a heterogeneous and diverse group of strains for which it cannot be guaranteed that experimental results regarding their antimicrobial potential would be representative of each individual strains; therefore, from a scientific standpoint, it is much more appropriate to discuss individual strains like EMA, and EMC, which also exert strong antibacterial activity on *E. amylovora* cells in vitro [40].

Previous CFCM data indicated that one of the most efficient antimicrobial ingredients produced by these *Xenorhabdus* strains are fabclavines [51,52]. Fabclavines are non-ribosomal antimicrobial peptides synthesized by an enzyme complex encoded by the fabclavine biosynthetic gene cluster (*fcl* BGC), both in the EMA and EMC strains [45,53]. The gene expression of the *fcl* BGC and other BGCs, which are responsible for the biosynthesis of secondary metabolites with antimicrobial potential during the so-called primary (or Phase-1) stage of these bacteria is Hfq-regulated. Phase 1 is the physiological state of EPB (*Xenorabdus* and *Photorhabdus*) species, when they exist as nemathode symbionts [47,50].

The aim of this work was to test the hypothesis that fabclavines are the most important antimicrobial components among AMPs produced by the EMA and EMC strains, which also exert an anti-fire blight effect. Neither of the inducible fabclavine-producing *fcl* KI mutants was expected to produce any antimicrobial compounds in the absence of arabinose induction. Consequently, if the hypothesis to be tested were correct, uninduced cultures should be inactive in the production of antimicrobial substances, while the respective induced cultures should exert antibacterial activity. As expected, CFCMs of both induced double mutant strains (Δ*hfq*; *fcl* KI) exerted a strong antibacterial effect on each of the fabclavine-sensitive test bacteria (including *E. amylovora* Ea1) in agar well diffusion experiments. In contrast, the CFCM from the uninduced *fcl* KI mutant EMC strain exhibited no antibacterial activity, while that of EMA showed a weak inhibitory effect. The LC-HRMS analysis also confirmed the presence of fabclavines in the induced cultures of both EMA and EMC *fcl* KI mutants. As expected, neither fabclavine nor its precursor molecules were found in the CFCMs of the non-induced *fcl* KI mutants. It could be seen that the antibacterial activities of CFCMs from the wild-type strains and those from the corresponding induced *fcl* KI mutant strains were very similar. Likewise, the bactericidal activity of CFCMs derived from the Δ*hfq* and the non-induced *fcl* KI mutant strains were also very similar. In fact, the CFCM of the EMA Δ*hfq* mutant shows a hardly detectable but not ignorable antibacterial activity that might be independent of the *hfq*-regulated BGCs.

As far as the in planta experiments, data appear to support the argument that antimicrobial peptides with strong bactericidal activity, such as fabclavines, should play a primary role in the preventive or curative treatment of fire blight. This is supported by the fact that the induced *fcl* KI mutants protected the flowers from infection by *E. amylovora*, providing up to 80% protection. These encouraging results can be compared with recently published data indicating the effects of AMPs examined in plants (primarily those derived from *Pantoea* bacteria) [34,54,55,56,57,58], as well as the effects of synthetic antimicrobial peptides such as BP100 [59] and its derivatives.

Considering that the bactericidal activity of fabclavines appears to be comparable to that of commercially available antibiotics, such as Kasumin 2 L and Streptomycin 20 WP, it is recommended that the possibility of practical agricultural applications of fabclavines against fire blight be seriously considered, particularly in light of the risk of the spread of antibiotic resistance genes [60] and the importance of the mobility-induced breakdown of the resistome structure [61]. Data support that the type strains of two entomopathogenic nematode symbiont species, *X. budapestensis* DSM16342 (EMA) and *X. szentirmaii* DSM16338 (EMC) [37,38], might be considered as important sources of fabclavines [51,52]. However, it is a practical question whether the application of purified fabclavine or fabclavine derivatives, or simply the whole or partially purified/fractionated CFCM preparations of some appropriate mutants (like our EMC *fcl* KI mutant), should be recommended for further studies. It remains necessary to explore the antibacterial effects of fabclavines and their practical applicability in the prevention of fire blight and even other bacterial infections [25].

## 4. Materials and Methods

### 4.1. Microbial Strains and Techniques

*X. budapestensis* DSM16342 (EMA) and *X. szenttirmaii* DSM16338 (EMC) wild-type strains were deposited at the German Collection of Microorganisms and Cell Cultures (DSMZ) [38] (Supplementary Table S3). Δ*hfq* mutants of both strains were generated to switch off the antimicrobial secondary metabolite production [41,42], and fabclavine promoter knock-in mutant strains (*fcl* KI) were subsequently generated from the Δ*hfq* mutants, which produce only fabclavines from the antimicrobial metabolite repertoire. During this process the *E. coli* S17-1 *λpir* strain was used to mobilize the KI vector into the EMA and EMC Δ*hfq* strains. S17-1 *λpir* was cultured at 37 °C in LB broth (5 g yeast extract, 10 g triptone, 10 g NaCl in 1 L distilled water) supplemented with 50 μg/mL streptomycin. For the cultivation of *Xenorhabdus* bacteria LB liquid medium, LB agar and LBTA agar plates were used. LBTA indicator plates are modified LB agar plates supplemented with 5 μg/mL bromothymol blue (BTB, Sigma-Aldrich, St. Louis, MO, USA) and 8 μg/mL 2,3,5-triphenyltetrazolium chloride (TTC, Sigma-Aldrich, St. Louis, MO, USA) and used to distinguish between secondary metabolite producing (phase 1) and non-producing (phase 2) variants [47] 1996; Leclerct & Boemare, 1991 [62]). *Fcl* KI mutants were cultured in media supplemented with kanamycin (Km) in final concentration of 50 μg/mL. In liquid cultures, all *Xenorhabdus* strains were grown at 30 °C.

For the in planta bioassay, *Erwinia amylovora* strain Ea1 was used [4]. The strain, which had been stored at −196 °C, was propagated on King-B medium (Merck, Darmstadt, Germany). Ea1 was also used in the agar well diffusion assay, for which it was cultured in LB medium at 30 °C in a rotary shaker at 180 rpm.

In the agar well diffusion assay, besides *E. amylovora* Ea1, three antibiotic-multiresistant bacterial strains were used. Extended spectrum beta-lactamase-producing *Es. coli*, *P. aeruginosa* and methicillin-resistant *S. pseudintermedius* were provided by P. Mag and Á. Jerzsele from the Department of Pharmacology and Toxicology of the University of Veterinary Medicine, Budapest and *E. faecium* (ATCC6057) was provided by Cs. Pál from the Hun-Ren Biological Research Centre, Szeged, Hungary. These bacterial strains were cultured in LB medium at 37 °C in a rotary shaker at 180 rpm.

### 4.2. Generation of Inducible Fabclavine-Producing Knock-In Mutant (fcl KI) Xenorhabdus budapestensis (EMA)and X. szentirmaii (EMC)

L-arabinose-inducible fabclavine-producing mutants (*fcl* KI) was generated from the Δ*hfq* mutants of EMA and EMC [42] by exchanging the native promoter of the *fcl* operon to the P*ara_BAD_* promoter according to the easyPACId technique [41,43].

#### 4.2.1. Exchange of the Promoter of the *fcl* Operon

Standard molecular biology procedures were carried out according to [63]. For the promoter exchange, a basic plasmid vector pBZS20 (Supplementary Table S1) containing the conditional replication origin R6Kγ of plasmid R6K, the *oriT* of plasmid RK2, the P*ara_BAD_* promoter and *araC* gene under the control of Para_C_ promoter and the APH(3′)-II family aminoglycoside O-phosphotransferase gene (a Km^R^ cassette from Tn*5*) was constructed. First, the 1.6 kb *Cla*I-*Pst*I fragment of pJKI625 [64] containing the P*ara_BAD_* promoter along with the *araC* gene was inserted into the *Cla*I-*Pst*I digested pSG76-K [65] resulting in pBZS19. Then, the 150 bp transfer origin (*oriT*) region of IncP plasmid RK2 (positions 51,115–51,276 bp) was cut out from pJKI664 [66] with *Acc*I and ligated into the *Cla*I site of pBZS19 resulting in pBZS20. Finally, the first 575 bp of the first gene of the *fcl* operon of EMA (Xbud_02634 see Supplementary Figure S1) was amplified using the primers fabcl_EMA_Ndfor and fabcl_EMA_Prev, while the first 675 bp of the homologous gene of EMC (Xsze_RS18325 see Supplementary Figure S1) was amplified with the primers fabcl_EMC_Ndfor and fabcl_EMC_Prev (Supplementary Table S4 and Supplementary Figure S1).

PCR amplifications were carried out using 0.2 μM primers, 0.2 mM dNTP, 1.5 units of Phusion polymerase (Thermo Scientific, Waltham, MA, USA) in 1× HF buffer in a final volume of 25 μL. As template 0.5 μL of EMA and EMC overnight cultures were applied. Cycling was the following: denaturation at 98 °C for 2 min, 30× (98 °C for 10 s, 55 °C for 30 s, 72 °C for 60 s), finalized by 72 °C for 5 min.

The amplicons were EtOH-precipitated, dried under vacuum for 15 min and dissolved in 20 μL of TE buffer (10 mM Tris 1 mM EDTA, pH 8.0), then were digested with *Nde*I-*Pst*I and ligated into pBZS20 linearized with *Nde*I-*Pst*I. For ligation reactions ~100 ng of pBZS20 DNA and 300–500 ng of digested amplicon DNA was used with 2.5 units of T4 ligase (Thermo Scientific, Waltham, MA, USA) in 1× ligase buffer in a final volume of 10 μL. The reaction mixes were overnight incubated at 16 °C and *Xba*I-*Xho*I-digested for 1 h before transformation to eliminate uncut pBZS20 plasmids.

An amount of 5 μL of ligation mixes were transformed into *E. coli* S17-1 *λpir* strain, allowing replication of the R6Kγ-based plasmids. Transformants were selected on LB plates supplemented with 50 μg/mL kanamycin. (Duchefa Biochemie, Haarlem, The Netherlands) by overnight incubation at 37 °C. Plasmid DNA was purified from 5 mL LB. + Km cultures grown overnight at 37 °C using QIAprep Spin Miniprep Kit (QIAgen, Hilden, Germany), and after verification by restriction analysis, appropriate clones were Sanger sequenced (Eurofins BIOMI Ltd. Gödöllő, Hungary) using primer ParaBfor (Supplementary Table S4) on ABI 3500xL Genetic Analyzer (Life Technologies, Carlsbad, CA, USA).

#### 4.2.2. Generating the *fcl* Knock-In Mutants

The resulting knock-in (KI) vectors pBZS29 and pBZS31 were mobilized into EMC and EMA Δ*hfq* mutants, respectively, from *E. coli* S17-1 *λpir*. Donor cultures were grown overnight at 37 °C in LB broth supplemented with streptomycin (Sm) and kanamycin (Km), while recipients were grown overnight at 30 °C in LB broth supplemented with ampicillin (Ap). The concentrations of the antibiotics were 50 μg/mL for Sm and Km and 150 μg/mL for Ap unless otherwise specified.

##### Construction of Fabclavine Producing Strains by Conjugation

For conjugation into the EMA Δ*hfq* strain, 100 μL of overnight (ON) S17-1 λ*pir*/pBZS31 donor culture was mixed with 900 μL of ON recipient culture, cells were spinned down at 3000 rpm in a benchtop centrifuge, washed twice in 0.9% NaCl solution and spread onto LB plates, which were incubated for 4 h at 30 °C, then bacterial lawn was resuspended with 3 mL of 0.9% NaCl solution. The mixture was plated on LB + Km + Sm (donor) and LB + Ap (recipient), while transconjugant recombinants were selected on LB + Km + 5 × Ap plates (containing 750 μg/mL Ap) incubated 48–72 h at 30 °C. Higher Ap concentration was necessary to inhibit the growth of the donor strain.

For conjugation into the EMC Δ*hfq* strain, ON S17-1 λ*pir*/pBZS29 donor culture was 5× diluted in 5 mL fresh LB + Sm + Km broth and grown to approx. 0.6–0.7 OD_600_. The recipient EMC Δ*hfq* strain grown overnight in LB + Ap was 4× diluted in 9 mL fresh LB + Ap and grown for 2 h at 30 °C to approx. 1.5 OD_600_. The 1 mL donor and 2 mL recipient cultures were mixed, centrifuged for 1 min at 3000 rpm and washed with 0.9% NaCl solution, then spread onto LB agar plates and incubated for 4 h at 37 °C, then overnight at 30 °C. After incubation, bacterial lawn from the LB plates was resuspended in 3 mL 0.9% NaCl solution. The mixture was titered on LB + Km + Sm (donor) and LB + Ap (recipient). Cells were centrifuged and spread onto LB + 2 × Km + 5 × Ap + X-gal agar plates (containing 100 μg/mL kanamycin, 750 μg/mL ampicillin and 0.004% X-gal (Thermo Scientific, Waltham, MA, USA)). Transconjugant recombinants were detected after 2 days of incubation at 30 °C as white colonies among the blue background colonies of the donor.

##### Verification of KI Recombinants

The Km^R^Ap^R^Sm^S^ colonies were tested with colony PCR using primers hfqseqfor and hfqseqrev indicative of both *Xenorhabdus* Δ*hfq* strains, and primers ParaBfor and fabcl_EMA_test or fabcl_EMC_test (Supplementary Table S4), indicative of the correct recombination. Colony PCRs were carried out using 0.2 μM primers, 0.2 mM dNTP, 2.5 mM MgCl_2_, 1 unit of Dream Taq polymerase and 1 μL of overnight culture in 1× Taq buffer (Thermo Scientific, Waltham, MA, USA) in 25 μL final volume. Cycling was the following: denaturation at 94 °C for 2 min, 35× (94 °C for 20 s, 55 °C for 30 s, 72 °C for 45 s), finalized by 72 °C for 5 min. From the clones proved positive for both PCRs, broader junction region of the insert was amplified with primers araC_5out and fabcl_EMA_test or fabcl_EMC_test using Phusion polymerase as described above. The obtained amplicons were purified using a QIAQuick PCR Purification Kit (QIAgen, Hilden, Germany) and Sanger sequenced with primer ParaBfor (Eurofins BIOMI Ltd. Gödöllő, Hungary) (Supplementary Figure S1 and sequence files in Supplementary Materials S1 and S2).

### 4.3. Preparation of Cell-Free Conditioned Media (CFCM)

For the preparation of CFCM, one loopful of bacteria was inoculated into 5 mL LB (with the addition of 50 μg/mL Km in case of *fcl* KI mutants) and incubated overnight at 30 °C on a rotary shaker. From this overnight culture, 2.5 mL was transferred into 50 mL sterile LB or LB + Km and incubated again overnight at 30 °C with shaking. From these cultures, 25 mL was transferred into 1 L LB and incubated at 30 °C and at a speed of 150 rpm for 5 days. This has been a several-year-long practice. No optimization experiments were conducted. During this 5-day period, the cultures have grown to mid-stationary phase, (for stable about another 6 days), the pH went up to about 8.6–9.6, and the cell density up to about OD600 = 1.0 ≈ 1 × 3 × 10^8^ cell/mL.

*Fcl* KI mutant cultures were prepared without induction or with the addition of 0.2% L-arabinose to induce fabclavine production. To obtain cell-free supernatant, cultures were centrifuged at 6000 rpm for 20 min at 4 °C and filtered through 0.22 μm Millipore filter. CFCMs were stored at 4 °C until use. On the basis of the several-year experience, it was known that fabclavin-containing *Xenorhabdus* CFCMs keep antibacterial activity for years at this temperature [42].

### 4.4. LC-MS Analysis of Induced and Non-Induced CFCMs of EMA and EMC fcl KI Strains

Induced and non-induced CFCMs obtained from the EMA and EMC *fcl* KI strains were subjected to targeted accurate-mass screening using a TripleTOF 5600+ hybrid quadrupole time-of-flight LC–MS/MS system (SCIEX, MA, USA) equipped with a DuoSpray ion source and coupled to a Shimadzu Prominence LC-20 UFLC system (Shimadzu, Kyoto, Japan) consisting of a binary pump, an autosampler, and a thermostated column compartment. Data acquisition and processing were performed using Analyst TF software version 1.7.1 (SCIEX Instruments, Framingham, MA, USA).

Chromatographic separation was achieved on a Waters XSelect CSH C18 column (150 mm × 4.6 mm, 3.5 µm). Gradient elution was performed using 0.1% formic acid in water as mobile phase A and 0.1% formic acid in acetonitrile (ACN) as mobile phase B. The initial mobile phase composition of 10% B was maintained for 1 min, followed by a linear increase to 95% B at 9 min. The mobile phase composition was maintained at 95% B from 9 to 12 min and then returned to the initial conditions (10% B) between 12 and 12.5 min. The initial conditions were maintained until 15 min. The flow rate was set to 1.0 mL/min. The column temperature was maintained at 40 °C, and the injection volume was 5 µL.

Nitrogen was used as the nebulizer gas (GS1), heater gas (GS2), and curtain gas (CUR), with the corresponding pressures set to 45, 45, and 40 psi, respectively. Data were acquired in positive electrospray ionization mode over an m/z range of 100–2000, with an accumulation time of 1 s. The source temperature was set to 450 °C, and the ion spray voltage was 5000 V. The declustering potential was set to 80 V. The mass resolution of the instrument was at least 25,000.

PeakView software version 2.2 (SCIEX, Redwood City, CA, USA) was used for targeted screening based on the elemental compositions of the expected compounds. For peptide-like compounds, the corresponding singly and multiply charged ions, including doubly and triply charged species, were considered. Putative matches were manually evaluated, and the co-elution of ions corresponding to different charge states of the same compound was verified.

### 4.5. Agar Well Diffusion Assay

The antibacterial activity of CFCMs derived from wild-type (wt), Δ*hfq* mutant, and induced and non-induced *fcl* KI mutant strains of EMA and EMC was evaluated using an agar well diffusion assay against *E. amylovora* Ea1, *E. coli*, *P. aeruginosa*, *S. pseudintermedius*, and *E. faecium* following the method of [67] with minor modifications. Experiments were carried out on LB agar plates in 90 mm Petri dishes containing 20 mL LB agar. Before the experiment, a loopful of each test bacteria from deep frozen stock cultures was inoculated into 5 mL LB medium and incubated overnight. From the ON cultures, 300 µL was mixed with 2.7 mL 0.75% soft agar maintained at approximately 45 °C and immediately overlaid onto the LB agar plate. After this layer solidified, wells with 8 mm diameter were made and filled with 100 µL CFCM. Plates with Ea1 were incubated at 30 °C; other plates were incubated at 37 °C ON corresponding to the optimal growth temperatures of the respective test organisms. After incubation, diameters of the inhibition zones were measured. Each test was performed in triplicate.

### 4.6. In Planta Bioassay: Flower Test

Flowers of the Idared apple variety susceptible to fire blight disease were collected at the balloon stage right before blooming to prevent any potential contamination in the field and placed individually in test tubes containing 10% (*w*/*v*) aqueous solution of sucrose and kept in transparent plastic boxes at 24 ± 0.5 °C and 85% relative humidity during the experiment, as described by [4] (Figure 10). The humidity was maintained by a 42% (*v*/*v*) glycerol solution (422 mL glycerol (Acidum-2 Kft., Debrecen, Hungary) + 578 mL water) at the bottom of the boxes. Under these microclimatic conditions, the flowers bloom simultaneously after 24 h.

Freshly bloomed flowers were treated preventatively with induced and non-induced CFCMs obtained from the *fcl* KI EMA and EMC mutant strains and with Kasumin 2 L and Streptomycin 20 WP (Table 3). Flowers were sprayed with various concentrations of the treatment agents (dilutions concerning CFCMs of EMA and EMC, and ppm concerning Kasumin 2 L and Streptomycin 20 WP formulations). For each treatment, a final volume of 50 mL of spray solution was used. The applied volume was 266.6 µL per 10 flowers, which corresponds to an application rate of approximately 800 L/ha.

Twenty-four hours after treatment, flower pistils were inoculated with 19 μL of 5 × 10^7^ CFU/mL suspension of Ea1 suspended in physiological saline using a 1 × 100 mm microcapillary (AA Labor Kft., Kunfehértó, Hungary). Thirty flowers per treatment were inoculated, whereas uninoculated flowers served as water-treated controls. On the 7th day after inoculation, all the flowers were sectioned longitudinally and scored for disease severity using the rating scale reported by [46], where 0 = no necrosis; 1 = detectable necrosis; 2 = up to half of the ovary was necrotic; 3 = necrosis affecting half to all the ovary; 4 = necrosis extending beyond ovary and into peduncle; and 5 = like 4, but bacterial ooze visible (Figure 6). Using the infection scale, infection frequencies and the severity of infection were estimated, and the infection index was calculated.

## 5. Conclusions

The CFCMs derived from the *fcl* KI mutant strains of both EMA and EMC exhibited a strong bactericidal effect against *E. amylovora* Ea1, which causes fire blight. This effect was also observed against multidrug-resistant strains of Gram-negative bacteria, such as *E. coli*, as well as Gram-positive bacteria, including *E. faecium* and S. *pseudointermedius* in vitro. The in planta tests proved that the prophylactic efficacy of the CFCMs of both induced *fcl* KI strains is unambiguous and comparable to that of the commercially available Kasumin 2 L and Streptomycin 20 WP formulations. Based on these results, we recommend further studies on potential strategies for controlling fire blight, whether using the whole CFCM from appropriately selected Δ*hfq fcl* KI *Xenorhabdus* mutants as biopreparations, or the selected end-products (fabclavines) or biosynthetic intermediates, as new antimicrobial agents. As the CFCM analysis indicates, different mutants may produce different intermediates with similar or even different cellular effects.

Before deciding to carry on this line of research, both CFCMs must undergo a comprehensive methodological evaluation to explore the antibacterial activities and their practical utility in controlling anti-fire blight, in accordance with the methodology developed within the framework of the international (Moroccan–Italian) research collaboration sponsored by the National Research Council of Italy (CNR) [10,25].

## Figures and Tables

**Figure 1:**
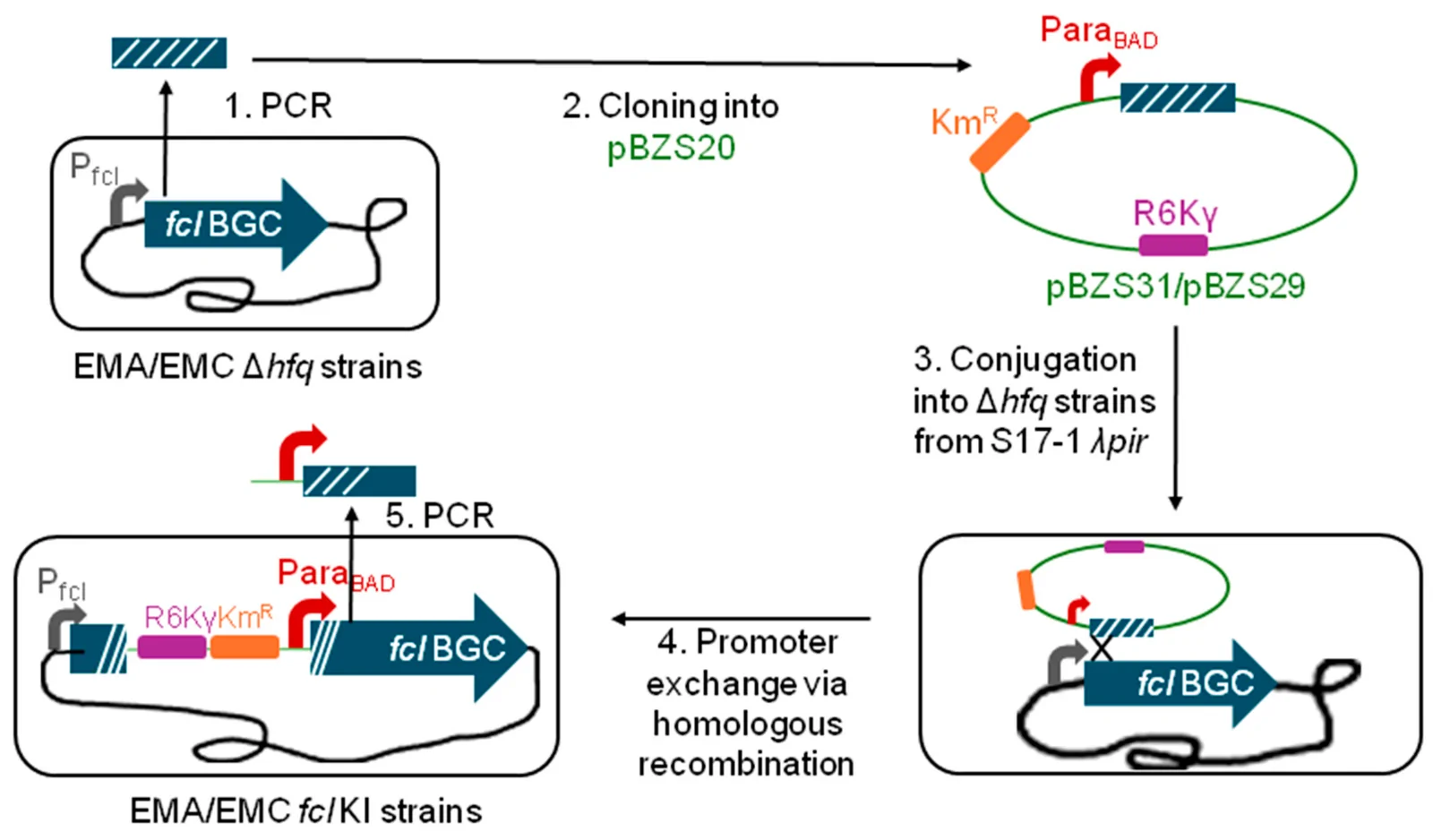
Schematic overview of the generation of L-arabinose-inducible fabclavine-producing knock-in (*fcl* KI) strains. Plasmid pBZS31 and pBZS29, were used for generating *fcl* KI double mutants in the Δ*hfq* mutant derivatives of *X. budapestensis* (EMA)- and *X. szentirmaii* (EMC)-type strains, respectively. ***1***. PCR amplification of the 0.6 kb fragments starting from the ATG start codon of the first gene of the respective *fcl* operon (striped blue box), providing homology for recombinational integration. ***2***. Cloning of the 0.6 kb amplicons downstream of the P*ara_BAD_* promoter and its Shine–Dalgarno box, thereby placing the *fcl* gene fragments under the control of the P*ara_BAD_* promoter, in a conditionally replicating (R6Kγ-based), mobilizable (via *oriT* of plasmid RK2), and selectable (Km^R^) basic plasmid vector pBZS20 (Supplementary Table S1). ***3***. Conjugal transfer of the resulting promoter exchange vectors pBZS29 and pBZS31 (Km^R^) from *E. coli* S17-1 *λpir* strain (Sm^R^), allowing their R6Kγ-based replication, into the two respective Δ*hfq* mutant *Xenorhabdus* strains (Ap^R^). ***4***. Chromosomal integration of the promoter exchange vectors via homologous recombination, which placed the entire *fcl* BGC under the control of the P*ara_BAD_* promoter in both *fcl* KI strains, making them double mutants (Δ*hfq* and *fcl* KI). ***5***. Amplification and sequencing of the vector–chromosome junctions, including the fusion of the P*ara_BAD_* promoter and the first gene of the *fcl* operon, to confirm the appropriate integration.

**Figure 2:**
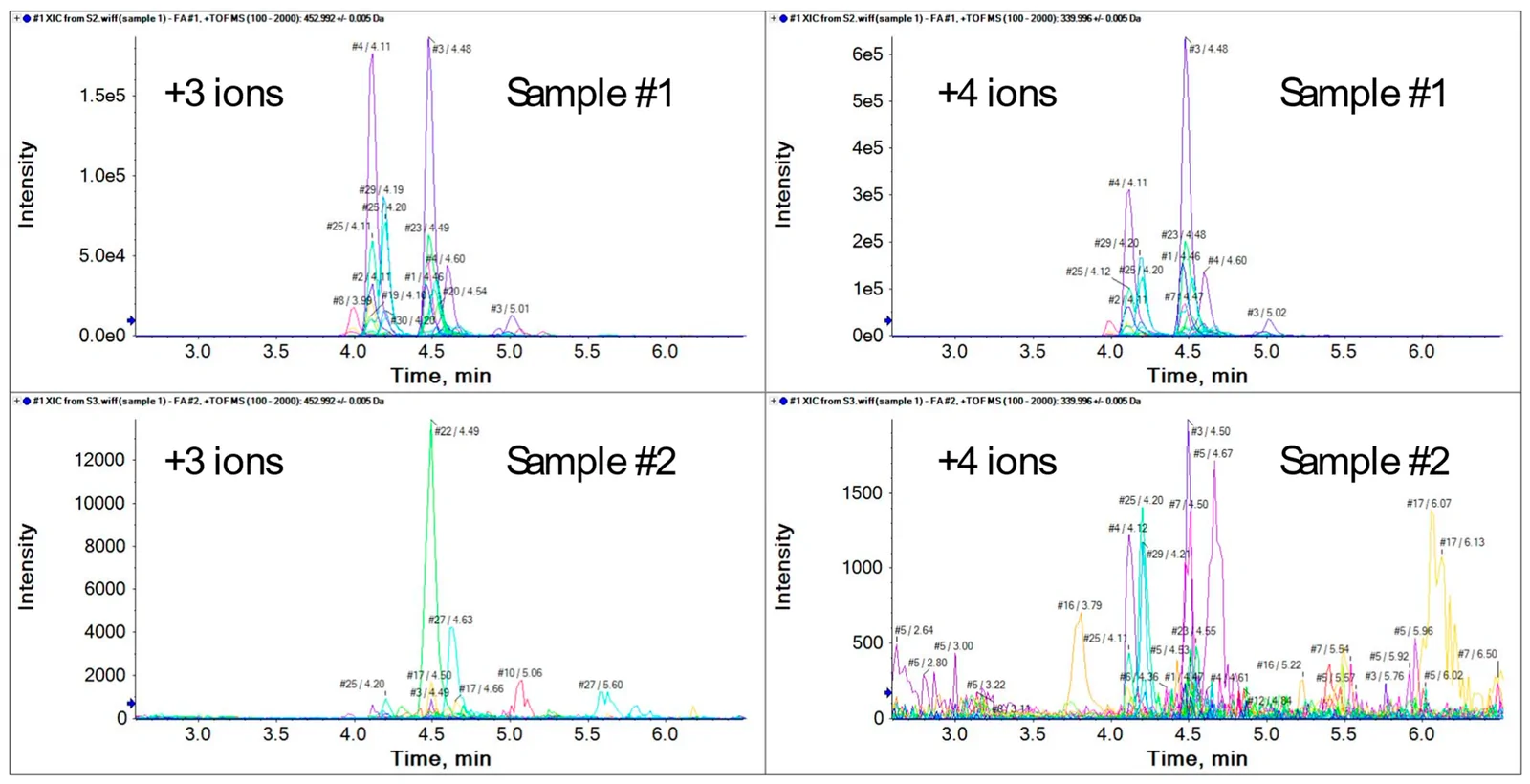
Extracted ion chromatograms (XICs) of the [M+3H]^3+^ and [M+4H]^4+^ ions of the positively identified target compounds in induced EMA *fcl* KI CFCM (sample #1) and the corresponding non-induced control sample (sample #2).

**Figure 3:**
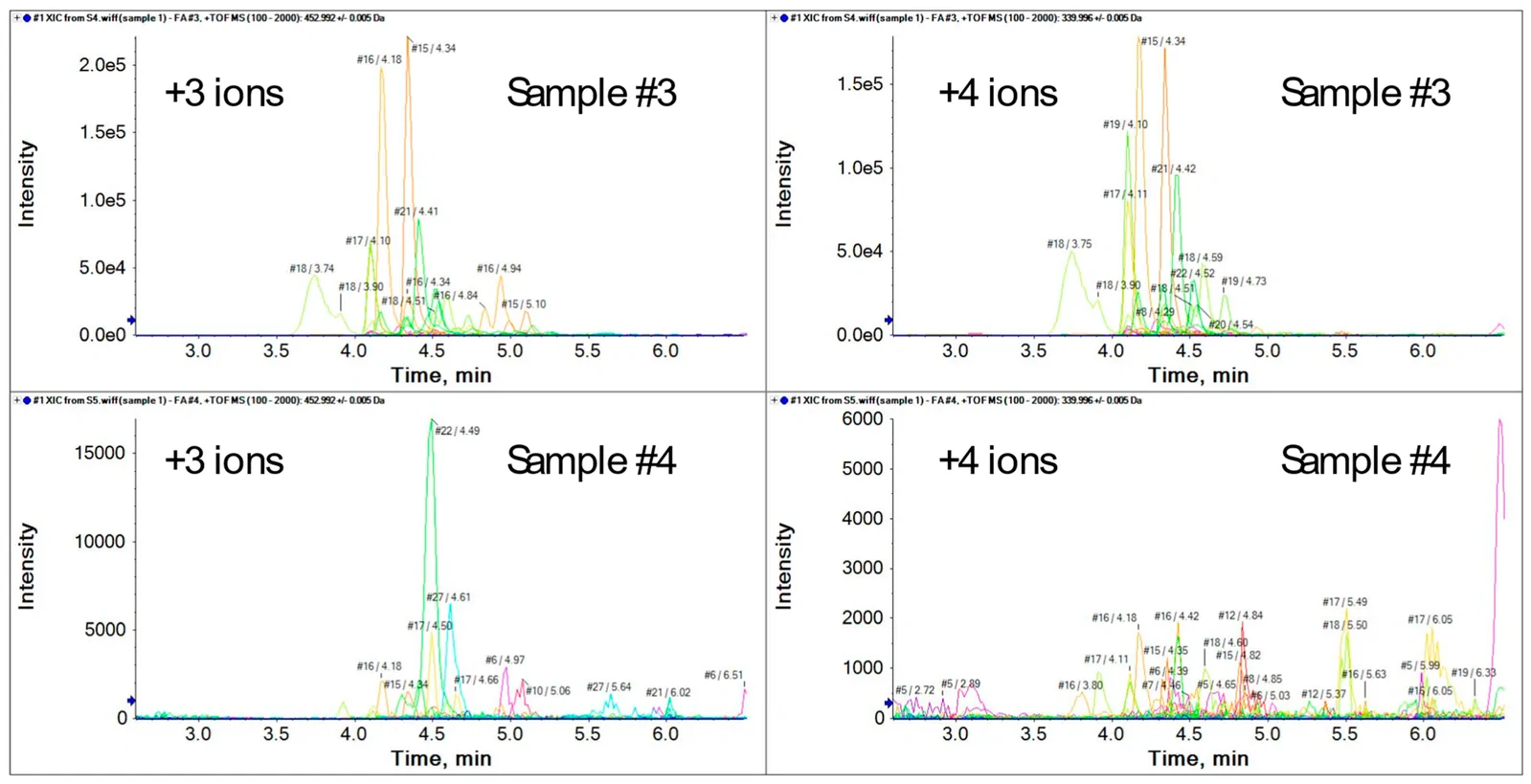
Extracted ion chromatograms (XICs) of the [M+3H]^3+^ and [M+4H]^4+^ ions of the positively identified target compounds in induced EMC *fcl* KI CFCM (sample #3) and the corresponding non-induced control sample (sample #4).

**Figure 4:**
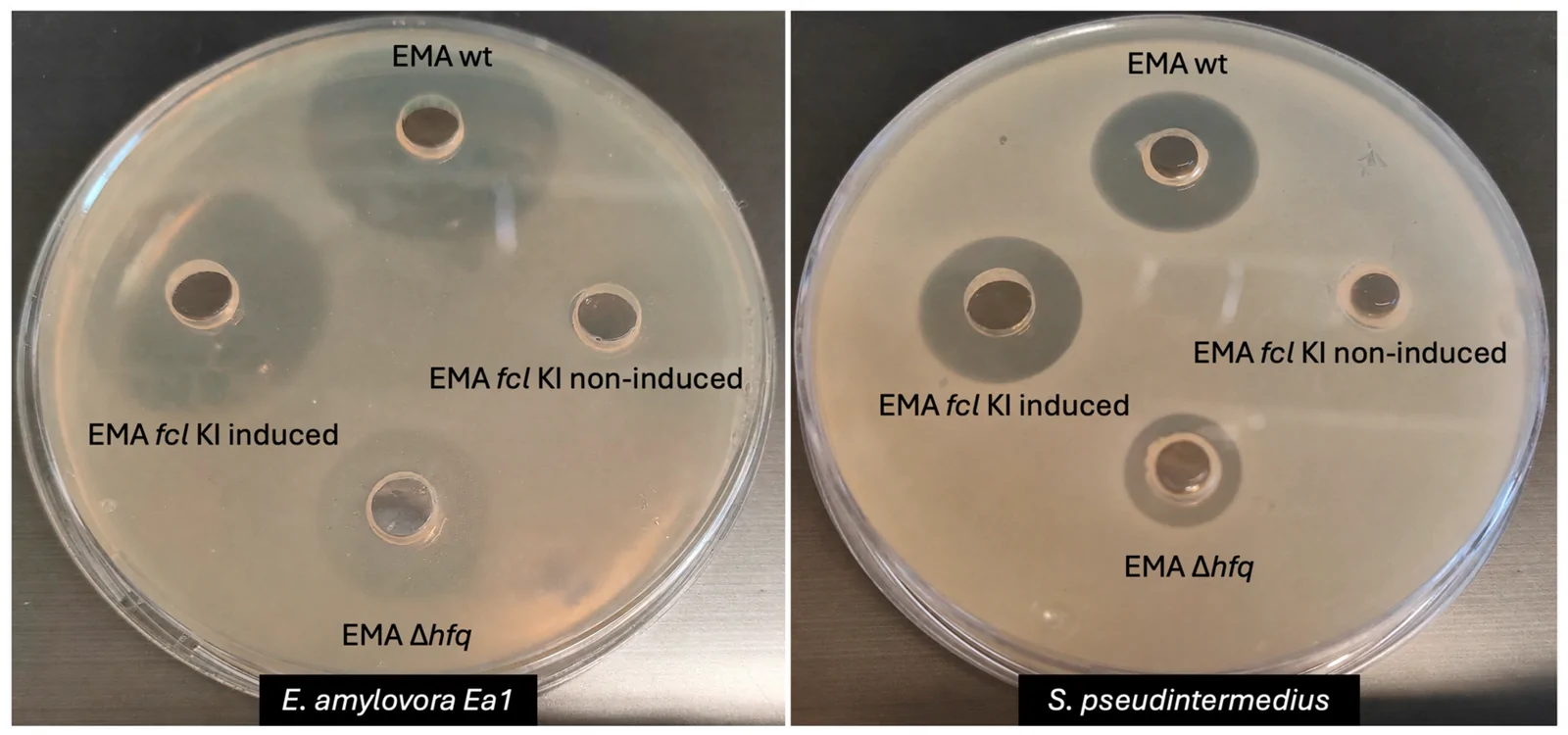
Agar well diffusion assay showing the antibacterial activity of CFCM from wild-type (wt), Δ*hfq* mutant and *fcl* KI mutant (induced and non-induced) strains of *X. budapestensis* (EMA) against *E. amylovora* Ea1 (**left panel**), and methicillin-resistant *S. pseudintermedius* (**right panel**). The upper and lower wells contain CFCMs from wt and Δ*hfq* mutant EMA, respectively, whereas the left and right wells contain CFCMs of induced and non-induced *fcl* KI mutant, respectively.

**Figure 5:**
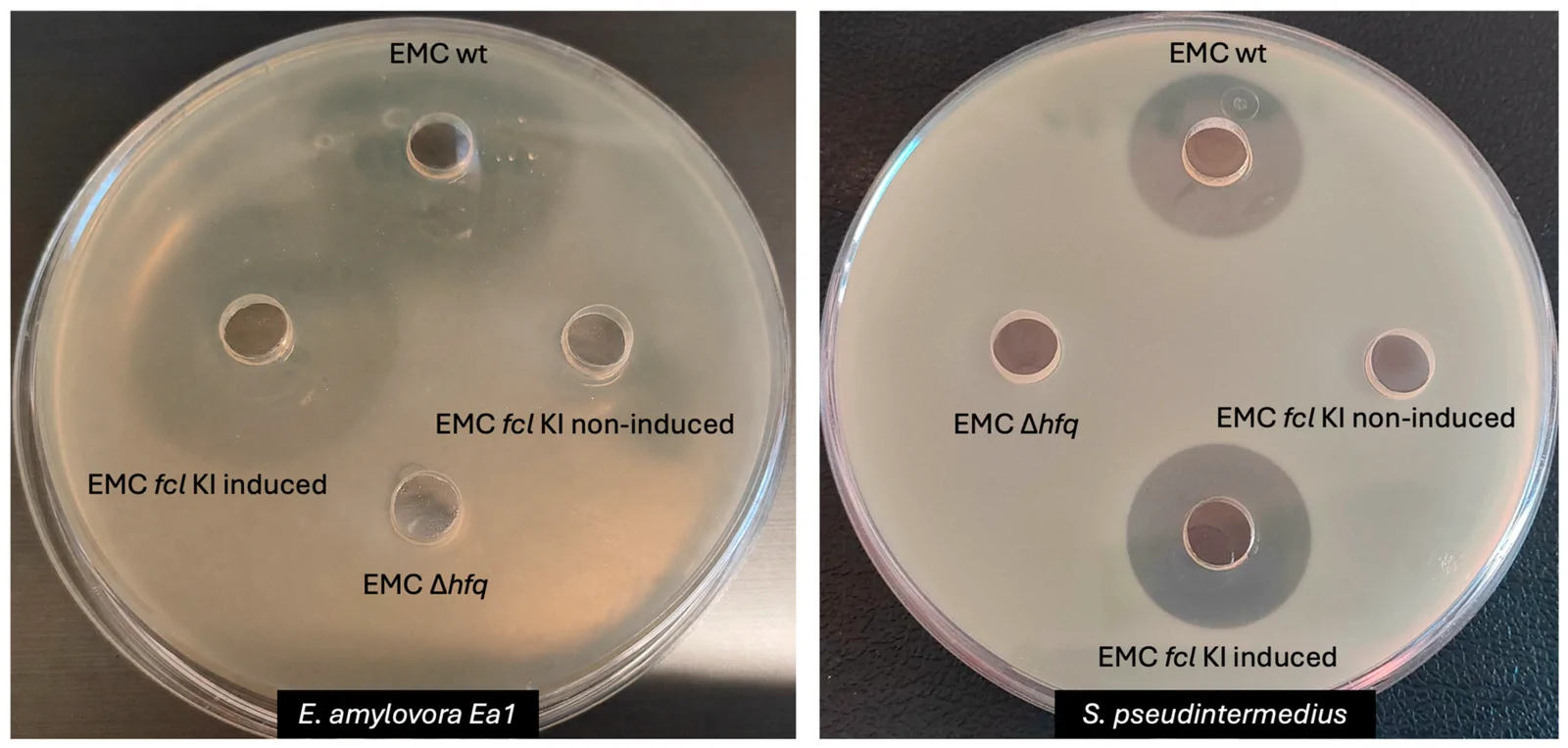
Agar well diffusion assay showing the antibacterial activity of CFCMs from wild-type (wt), Δ*hfq* mutant and *fcl* KI mutant (induced and non-induced) strains of *Xenorhabdus szentirmaii* (EMC) against *Erwinia amylovora* Ea1 *(***left panel**) and methicillin-resistant *S. pseudintermedius* (**right panel**).

**Figure 6:**
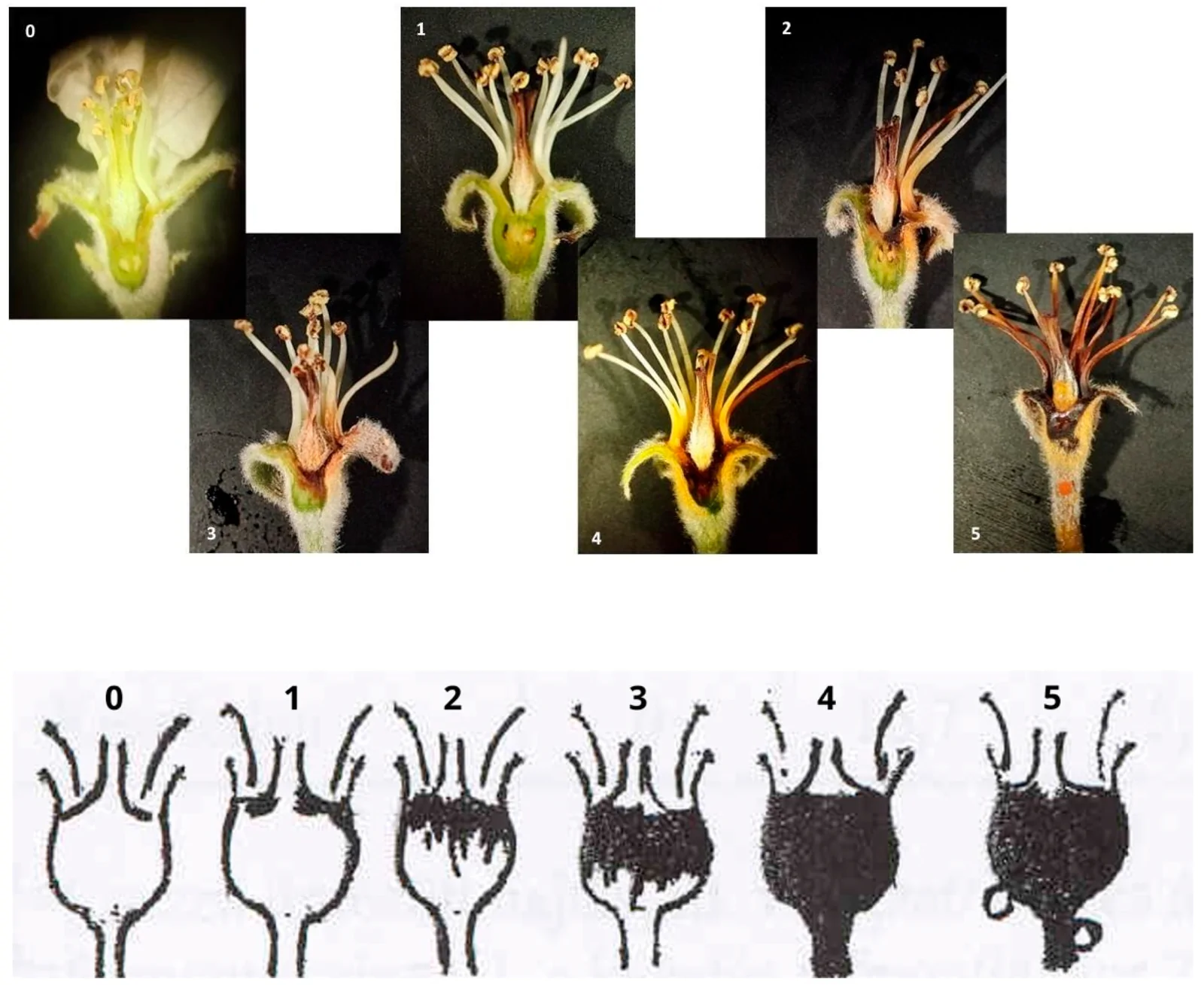
Infection severity rating scale according to [46] and representative fire blight symptoms observed in the present study (Photo: L. Sz. Földes): 0 = no necrosis; 1 = detectable necrosis; 2 = up to half of the ovary was necrotic; 3 = necrosis affecting half to all the ovary; 4 = necrosis extending beyond the ovary and into the peduncle; and 5 = like 4, but bacterial ooze visible.

**Figure 7:**
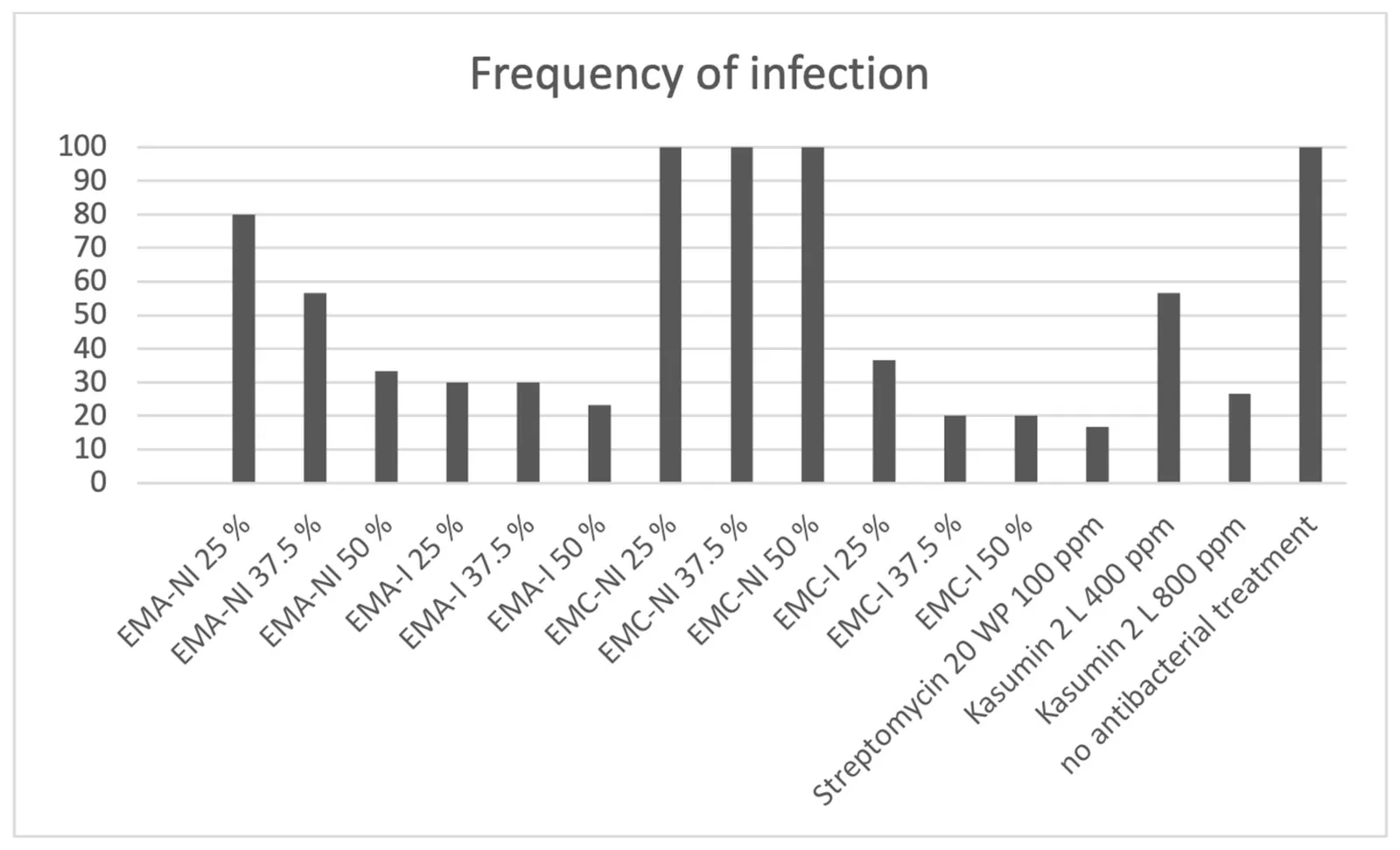
Frequency of fire blight infection in apple flowers seven days after inoculation with *E. amylovora* strain Ea1 following preventive treatment with cell-free culture media (CFCM) obtained from induced (I) and non-induced (NI) *fcl* KI mutants of *X. budapestensis* (EMA), and *X. szentirmaii* (EMC) at different concentrations. Streptomycin 20 WP and Kasumin 2 L served as positive controls, while untreated flowers served as the negative control.

**Figure 8:**
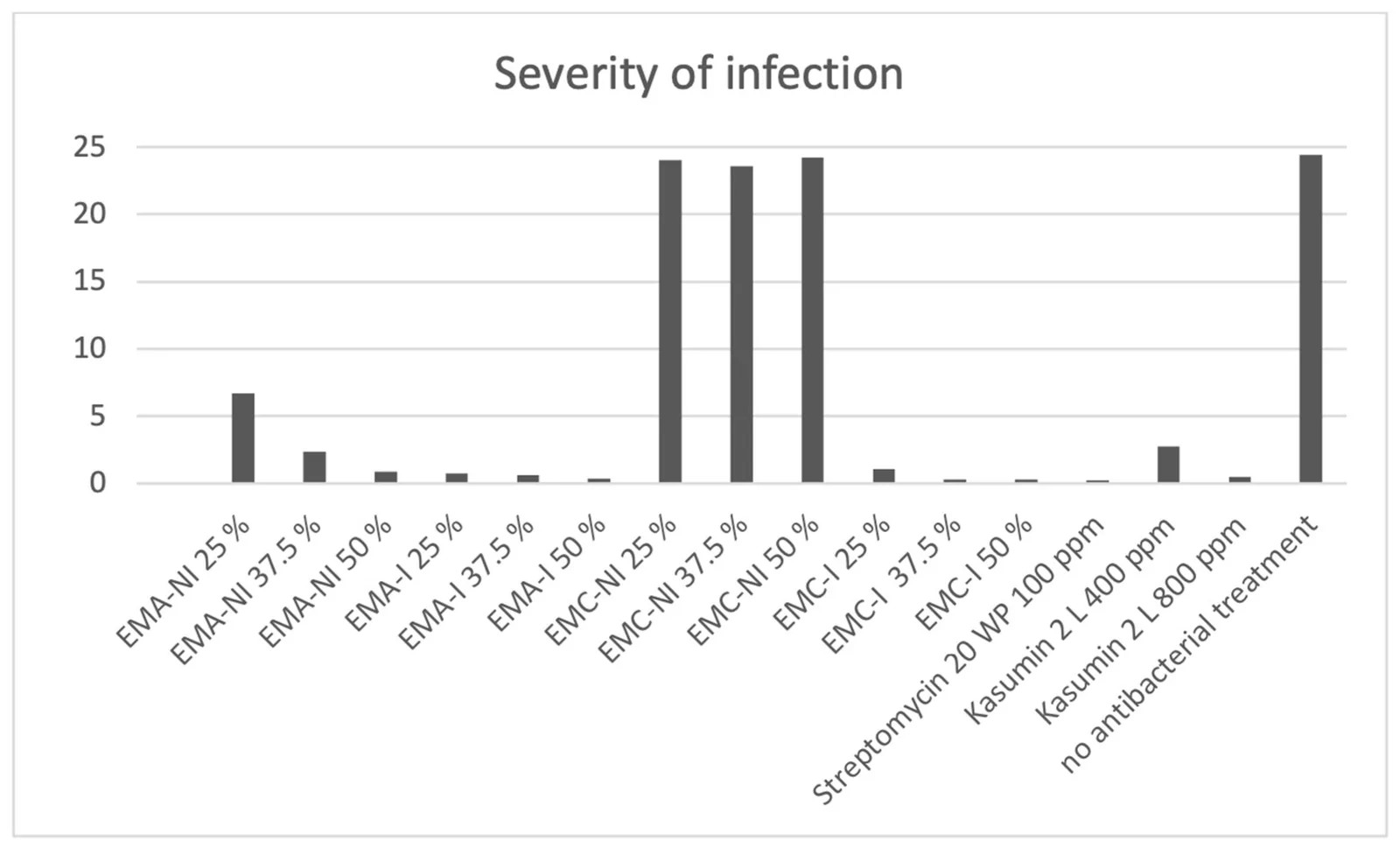
Severity of fire blight symptoms in apple flowers seven days after inoculation with *E. amylovora* strain Ea1 following preventive treatment with cell-free culture media (CFCM) obtained from induced (I) and non-induced (NI) *fcl* KI mutants of *X. budapestensis* (EMA), and *X. szentirmaii* (EMC) at different concentrations. Streptomycin 20 WP and Kasumin 2 L served as positive controls, while untreated flowers served as the negative control.

**Figure 9:**
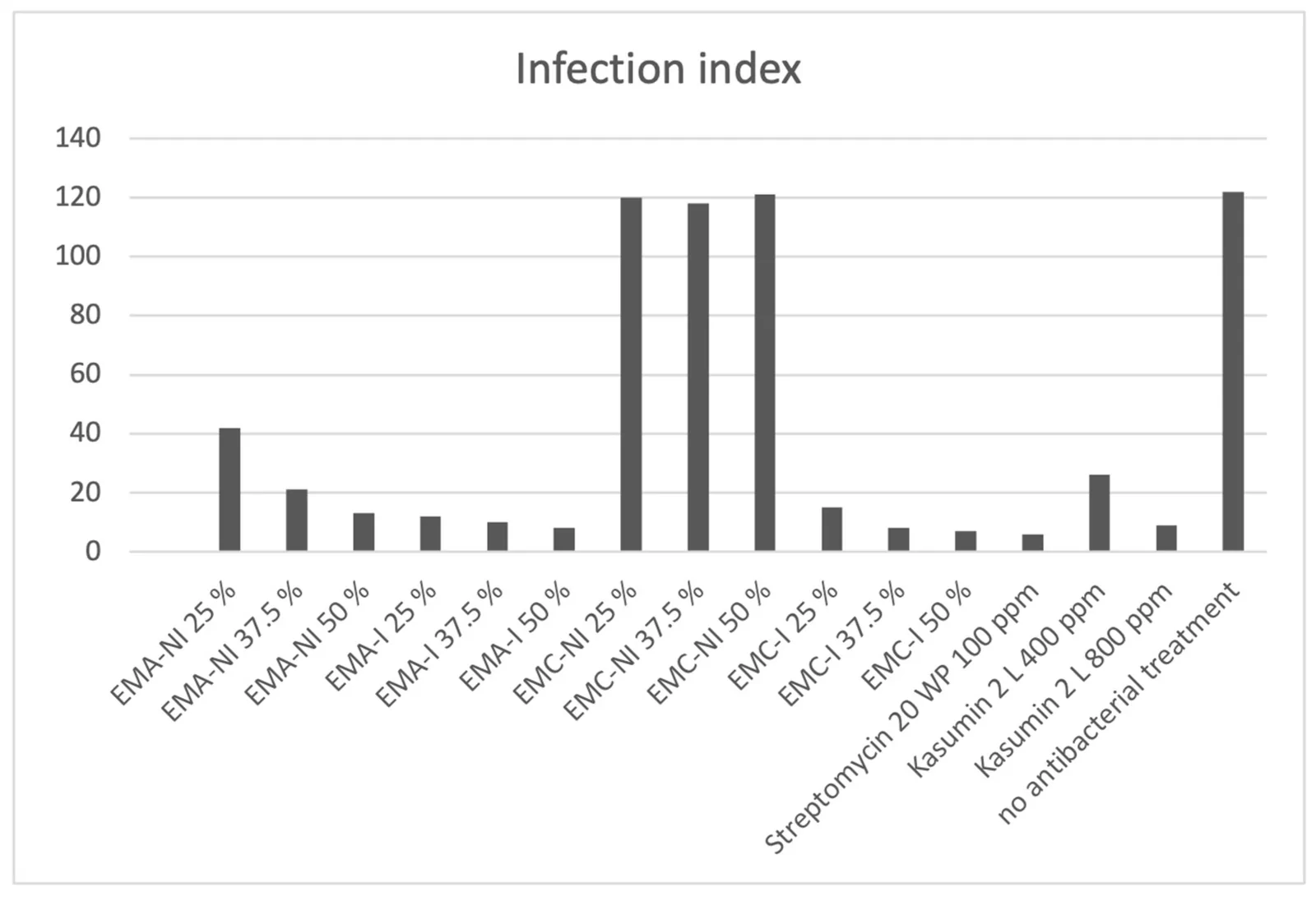
Infection index of fire blight in apple flowers seven days after inoculation with *E. amylovora* strain Ea1 following preventive treatment with cell-free culture media (CFCM) obtained from induced (I) and non-induced (NI) *fcl* KI mutants of *X. budapestensis* (EMA) and *X. szentirmaii* (EMC) at different concentrations. Streptomycin 20 WP and Kasumin 2 L served as positive controls, while untreated flowers served as the negative control.

**Figure 10:**
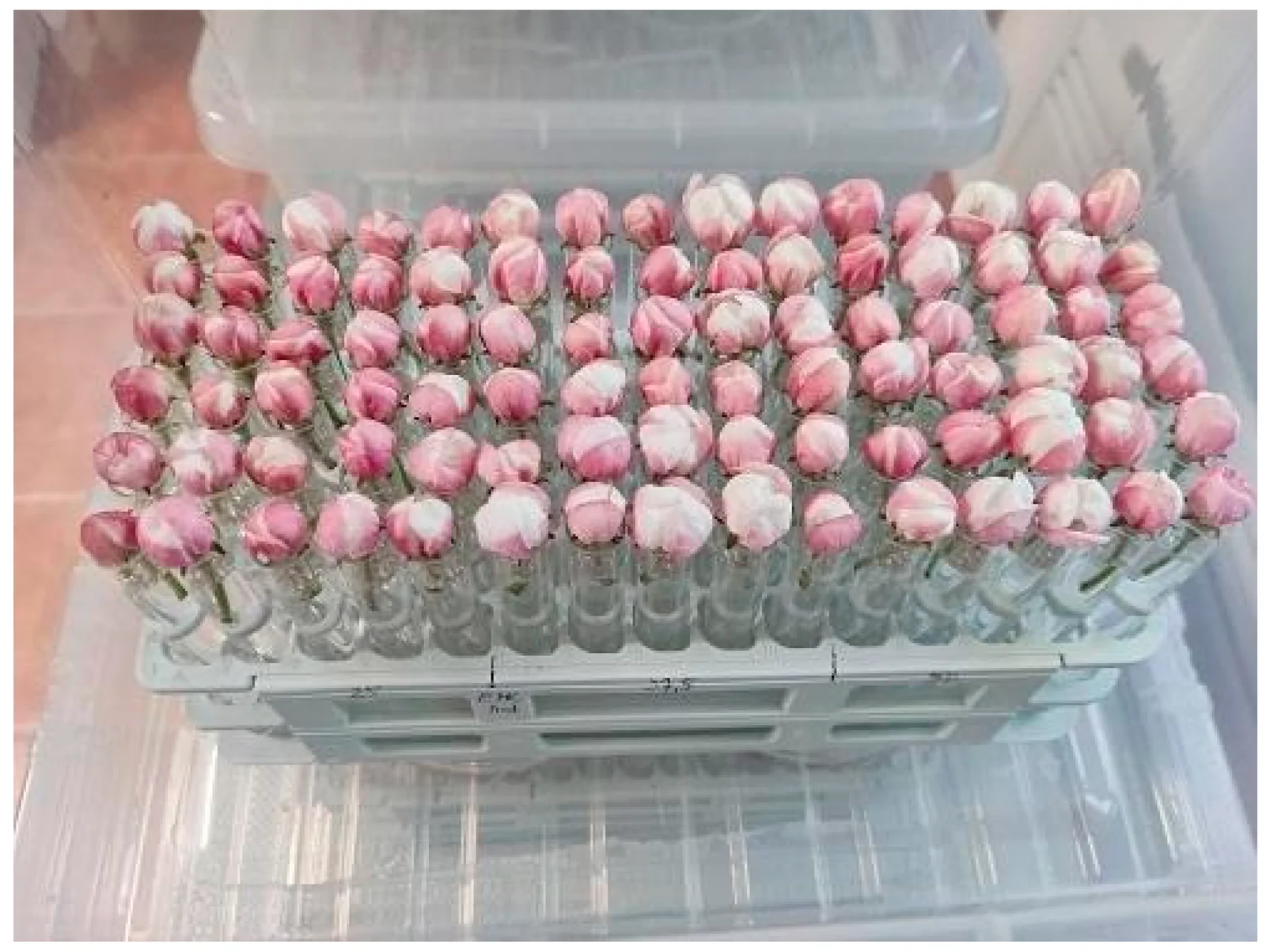
Incubation chambers set up to provide necessary microclimatic conditions for the flowers (Photo: L. Sz. Földes).

**Table 1:** Compound list of fabclavine derivatives produced by *Xenorhabdus* bacteria identified by [45].

Compound	Formula	[M+H]^+^	[M+2H]^2+^	[M+3H]^3+^	[M+4H]^4+^	[M+5H]^5+^
#1	C_70_H_125_N_13_O_13_	1356.959	678.983	452.992	339.996	272.198
#2	C_67_H_123_N_15_O_13_	1346.949	673.978	449.655	337.493	270.196
#3	C_68_H_121_N_13_O_12_	1312.933	656.970	438.316	328.989	263.393
#4	C_65_H_119_N_15_O_12_	1302.924	651.966	434.980	326.487	261.391
#5	C_62_H_108_N_12_O_13_	1229.823	615.415	410.613	308.212	246.771
#6	C_59_H_106_N_14_O_13_	1219.814	610.411	407.277	305.709	244.769
#7	C_60_H_104_N_12_O_12_	1185.797	593.402	395.938	297.205	237.966
#8	C_57_H_102_N_14_O_12_	1175.787	588.397	392.601	294.703	235.964
#9	C_78_H_142_N_14_O_13_	1484.095	742.551	495.370	371.780	297.625
#10	C_76_H_138_N_14_O_12_	1440.069	720.538	480.695	360.773	288.820
#11	C_75_H_140_N_16_O_13_	1474.086	737.547	492.034	369.277	295.623
#12	C_73_H_136_N_16_O_12_	1430.060	715.534	477.359	358.271	286.818
#13	C_73_H_138_N_16_O_12_	1432.075	716.541	478.030	358.775	287.221
#14	C_75_H_142_N_16_O_13_	1476.102	738.555	492.706	369.781	296.027
#15	C_54_H_102_N_12_O_12_	1111.781	556.394	371.266	278.701	223.162
#16	C_53_H_100_N_12_O_13_	1113.761	557.384	371.926	279.196	223.558
#17	C_57_H_104_N_14_O_12_	1177.803	589.405	393.273	295.207	236.367
#18	C_56_H_102_N_14_O_13_	1179.782	590.395	393.933	295.701	236.763
#19	C_59_H_108_N_14_O_13_	1221.829	611.418	407.948	306.213	245.172
#20	C_60_H_106_N_12_O_12_	1187.813	594.410	396.610	297.709	238.369
#21	C_59_H_104_N_12_O_13_	1189.792	595.400	397.269	298.204	238.765
#22	C_62_H_110_N_12_O_13_	1231.839	616.423	411.285	308.716	247.174
#23	C_68_H_123_N_13_O_12_	1314.948	657.978	438.988	329.493	263.796
#24	C_67_H_121_N_13_O_13_	1316.928	658.968	439.648	329.988	264.192
#25	C_65_H_121_N_15_O_12_	1304.939	652.973	435.652	326.991	261.794
#26	C_64_H_119_N_15_O_13_	1306.918	653.963	436.311	327.485	262.190
#27	C_70_H_127_N_13_O_13_	1358.975	679.991	453.664	340.500	272.601
#28	C_69_H_125_N_13_O_14_	1360.954	680.981	454.323	340.994	272.997
#29	C_67_H_125_N_15_O_13_	1348.965	674.986	450.327	337.997	270.599
#30	C_66_H_123_N_15_O_14_	1350.945	675.976	450.987	338.492	270.995
#31	C_69_H_129_N_15_O_14_	1392.992	697.000	465.003	349.004	279.405
#32	C_72_H_131_N_13_O_14_	1403.001	702.004	468.339	351.506	281.406

**Table 2:** Inhibition zones of CFCMs of the wild-type (wt), Δ*hfq* mutant and *fcl* KI mutant (induced and non-induced) strains of *X. budapestensis* (EMA), and *X. szentirmaii* (EMC) in mm (average ± standard error, *n* = 3) against extended spectrum beta-lactamase producing *Escherichia coli*, *P. aeruginosa*, methicillin resistant *S. pseudintermedius*, *E. faecium* (ATCC6057) and *E. amylovora* Ea1. 0 = no detectable inhibition zone. The color scale represents inhibition zone diameter, ranging from green (no detectable inhibition) to dark red (largest inhibition zone).

CFCM	Test Bacteria				
	*E. coli*	*P. aeruginosa*	*S. pseudintermedius*	*E. faecium*	*E. amylovora* Ea1
EMA wt	20.0 ± 0	0	22.0 ± 0	15.7 ± 0.3	32.3 ± 0.3
EMA Δ*hfq*	12.3 ± 0.7	0	13.7 ± 0.3	0	20.3 ± 0.3
EMA *fcl* KI non-induced	0	0	0	0	0
EMA *fcl* KI induced	17.7 ± 0.3	0	19.7 ± 0.3	15.7 ± 0.3	30.7 ± 0.7
EMC wt	17.3 ± 0.3	0	22.0 ± 0.6	11.0 ± 0	32.3 ± 0.3
EMC Δ*hfq*	0	0	0	0	0
EMC *fcl* KI non-induced	0	0	0	0	13.3 ± 0.3
EMC *fcl* KI induced	21.0 ± 0.6	0	22.7 ± 0.7	13.3 ± 0.3	33.7 ± 1.2

**Table 3:** Treatments tested to prevent fire blight on apple flowers.

Treatment with Cell-Free Conditioned Culture Media (CFCM)				DOSES
No.	Name	Cultured Bacteria	Induced (I)/ Non-Induced (NI)	CFCM
				*v*/*v*%
1	EMA-NI	Δ*hfq fcl* KI double mutant	NI	25%
2	EMA-NI	Δ*hfq fcl* KI double mutant	NI	37.5%
3	EMA-NI	Δ*hfq fcl* KI double mutant	NI	50%
4	EMA-I	Δ*hfq fcl* KI double mutant	I	25%
5	EMA-I	Δ*hfq fcl* KI double mutant	I	37.5%
6	EMA-I	Δ*hfq fcl* KI double mutant	I	50%
7	EMC-NI	Δ*hfq fcl* KI double mutant	NI	25%
8	EMC-NI	Δ*hfq fcl* KI double mutant	NI	37.5%
9	EMC-NI	Δ*hfq fcl* KI double mutant	NI	50%
10	EMC-I	Δ*hfq fcl* KI double mutant	I	25%
11	EMC-I	Δ*hfq fcl* KI double mutant	I	37.5%
12	EMC-I	Δ*hfq fcl* KI double mutant	I	50%
				ppm antibiotics
13	Streptomycin 20 WP			100 ppm (0.5 kg/ha)
14	Kasumin 2 L			400 ppm (20 L/ha) *
15	Kasumin 2 L			800 ppm (40 L/ha) *
16	Positive (*E. amylovora* Ea1) control			5 × 10^7^ cell/mL
17	Negative ((*E. amylovora* Ea1-free) control			Tap water

* Ten and twenty times the approved dose for apples.

## Data Availability

The original contributions presented in this study are included in the article/Supplementary Materials. Further inquiries can be directed to the corresponding author.

## Supplementary Materials

The following supporting information can be downloaded at: https://www.mdpi.com/article/10.3390/antibiotics15100961/s1, Figure S1: Generation and verification of the *fcl* KI mutant *X. budapestensis* and *X. szentirmaii*; Figure S2: Agar well diffusion assays; Table S1: List and relevant features of plasmids used; Table S2: In planta bioassay evaluation; Table S3: List of bacterial strains used; Table S4: List of oligonucleotide primers used; Supplementary Material S1: Sequence file of the verification of the *fcl* KI in EMA; Supplementary Material S2: Sequence file of the verification of the *fcl* KI in EMC; Supplementary Material S3: LC-MS analysis XICs.

## Abbreviations

The following abbreviations are used in this manuscript:

AMP	Antimicrobial peptide
Ap^R^	Ampicillin resistance
BGC	Biosynthetic gene cluster
BTB	Bromothymol blue
CFCM	Cell-free conditioned media
Δ*hfq* mutants	Mutant strains generated from EMA and EMC by the deletion of the *hfq* gene
DSMZ	German Collection of Microorganisms and Cell Cultures
Ea1	*Erwinia amylovora* strain Ea1
easyPACId	Easy Promoter Activated Compound Identification
EMA	*Xenorhabdus budapestensis* (DSM16342).
EMC	*Xenorhabdus szentirmaii* (DSM16338).
EPB	Entomopathogenic bacteria
EPN	Entomopathogenic nematode
*fcl* KI mutants	L-arabinose-inducible fabclavine-producing knock-in mutant strains of EMA and EMC
Km^R^	Kanamycin resistance
LC-HRMS	Liquid chromatography-high resolution mass spectrometry
ON	Overnight culture
Sm^R^	Streptomycin resistance
TTC	2,3,5-triphenyltetrazolium chloride
wt	Wild-type
XIC	extracted ion chromatogram
