# Supplementary material for: Anti-Fireblight Potential of Fabclavines, Synthesized by the Type Strains of Xenorhabdus szentirmaii and Xenorhabdus budapestensis
Source: Antibiotics (Basel). 2026 Sep 28;15(10):961. doi: 10.3390/antibiotics15100961 (PMC13700878; doi:10.3390/antibiotics15100961)
Supplement: Supplementary file 1 [file antibiotics-15-00961-s001.zip › Supplementary Figure S1.pdf]

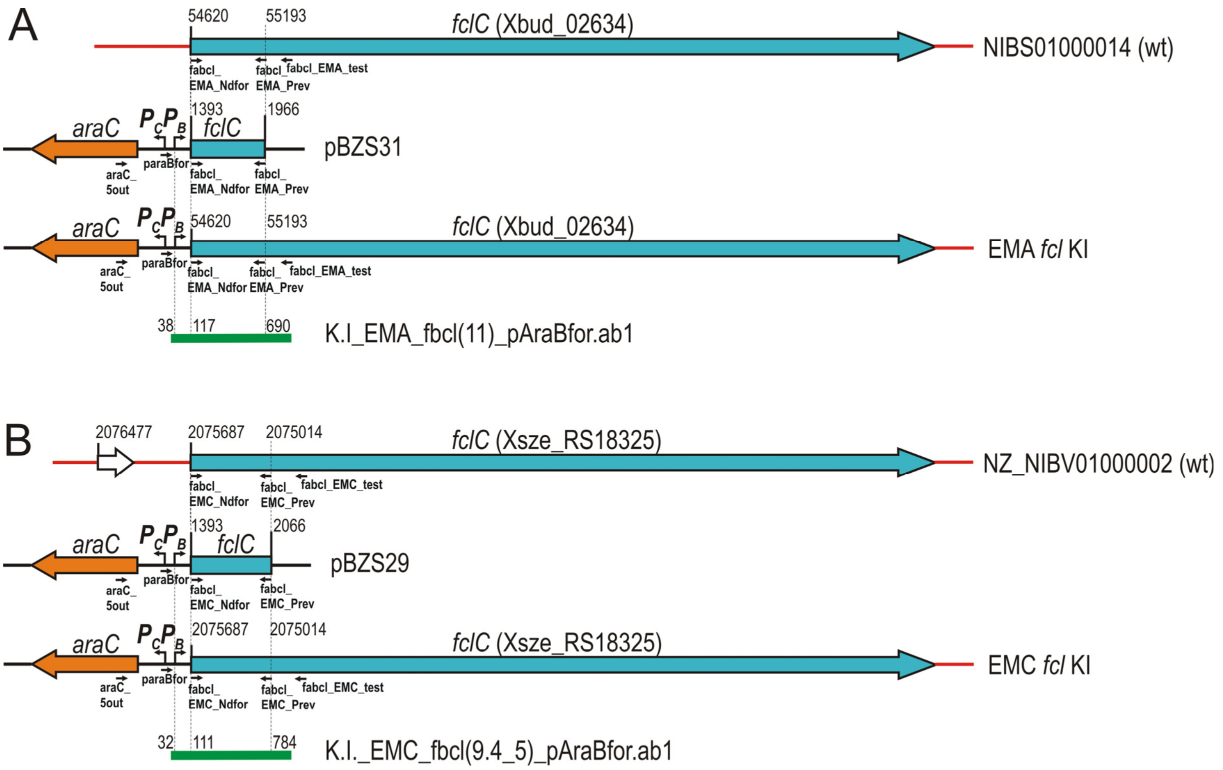

Figure S1. Generation and verification of the *fcl* KI mutant *X. budapestensis* (A) and *X. szentirmaii* (B). The wild-type (wt) genetic constitution and the coordinates are indicated according to the contigs available in public databases under the designation NIBS01000014 (EMA) and NZ_NIBV01000002 (EMC). The first gene of the *fcl* operon (*fclC*; numbering follows the original annotation of the individual contigs) and its 0.6-kb amplified regions are marked in blue. The chromosome is represented by a red line, whereas the plasmid DNA is shown in black. P_C and P_B denote the $Para_C$ and $Para_{BAD}$ promoters, respectively. Small horizontal arrows indicate primers used for amplification and/or sequencing (Supplementary Table S4.). Vertical dashed lines connect the corresponding positions. The base positions are indicated above the graphs. The green horizontal lines below the maps of *fcl* KI genomic constitution represent the chromatograms (provided as Supplementary Materiel 1 and 2 files) obtained from Sanger sequencing of PCR amplicons from *fcl* KI mutants using the araC_5out and fabcl_EMA/EMC_test primers for amplification of the integration region. The amplicons were sequenced with the paraBfor primer.
