# Supplementary material for: Anti-Fireblight Potential of Fabclavines, Synthesized by the Type Strains of Xenorhabdus szentirmaii and Xenorhabdus budapestensis
Source: Antibiotics (Basel). 2026 Sep 28;15(10):961. doi: 10.3390/antibiotics15100961 (PMC13700878; doi:10.3390/antibiotics15100961)
Supplement: Supplementary file 1 [file antibiotics-15-00961-s001.zip › Supplementary Figure S2.pdf]

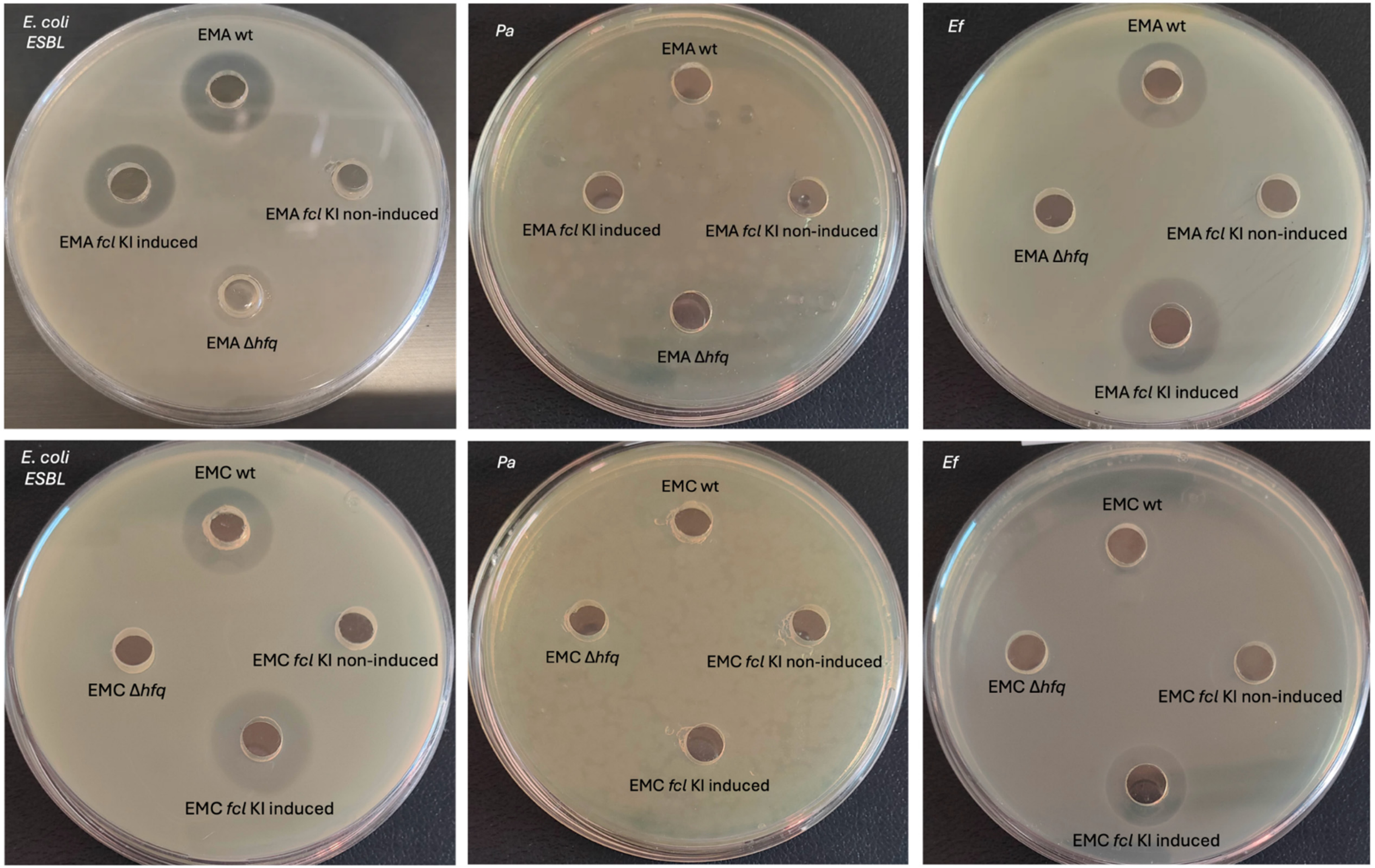


**Figure S2.** Agar well diffusion assay showing the antibacterial activity of cell-free culture media (CFCM) from wild-type (wt), Δ*hfq* mutant and *fcl* KI mutant (induced and non-induced) strains of *Xenorhabdus budapestensis* (EMA) *Xenorhabdus szentirmaii* (EMC) against extended spectrum β-lactamase producing *Escherichia coli (E. coli ESBL), Pseudomonas aeruginosa (Pa)* and *Enterococcus faecium (Ef).*
