# Supplementary material for: Anti-Fireblight Potential of Fabclavines, Synthesized by the Type Strains of Xenorhabdus szentirmaii and Xenorhabdus budapestensis
Source: Antibiotics (Basel). 2026 Sep 28;15(10):961. doi: 10.3390/antibiotics15100961 (PMC13700878; doi:10.3390/antibiotics15100961)
Supplement: Supplementary file 1 [file antibiotics-15-00961-s001.zip › Supplementary Material S3_LC-MS.pdf]

Table S3/1. Summary of the positively identified compounds. Sample #1: induced EMA *fcl* KI CFCM, sample #3: induced EMC *fcl* KI CFCM. For the list of compounds see Table 1.

Sample	Compound	Confirming charge states
#1	#1 ($C_{70}H_{125}N_{13}O_{13}$)	+2,+3,+4+5
#1	#2($C_{67}H_{123}N_{15}O_{13}$)	+2,+3,+4+5
#1	#3 ($C_{68}H_{121}N_{13}O_{12}$)	+2,+3,+4+5
#1	#4 ($C_{65}H_{119}N_{15}O_{12}$)	+1,+2,+3,+4+5
#1	#6 ($C_{59}H_{106}N_{14}O_{13}$)	+2,+3,+4+5
#1	#7 ($C_{60}H_{104}N_{12}O_{12}$)	+3,+4+5
#1	#8 ($C_{57}H_{102}N_{14}O_{12}$)	+2,+3,+4+5
#1	#17 ($C_{57}H_{104}N_{14}O_{12}$)	+2,+3,+4+5
#1	#18 ($C_{56}H_{102}N_{14}O_{13}$)	+2,+3,+4+5
#1	#19 ($C_{59}H_{108}N_{14}O_{13}$)	+2,+3,+4+5
#1	#22 ($C_{62}H_{110}N_{12}O_{13}$)	+2,+3,+4+5
#1	#23 ($C_{68}H_{123}N_{13}O_{12}$)	+2,+3,+4+5
#1	#25 ($C_{65}H_{121}N_{15}O_{12}$)	+2,+3,+4+5
#1	#27 ($C_{70}H_{127}N_{13}O_{13}$)	+2,+3,+4+5
#1	#29 ($C_{67}H_{125}N_{15}O_{13}$)	+2,+3,+4+5
#1	#30 ($C_{66}H_{123}N_{15}O_{14}$)	+2,+3,+4+5
#3	#15 ($C_{54}H_{102}N_{12}O_{12}$)	+2,+3,+4
#3	#16 ($C_{53}H_{100}N_{12}O_{13}$)	+2,+3,+4
#3	#17 ($C_{57}H_{104}N_{14}O_{12}$)	+2,+3,+4+5
#3	#18 ($C_{56}H_{102}N_{14}O_{13}$)	+2,+3,+4+5
#3	#19 ($C_{59}H_{108}N_{14}O_{13}$)	+2,+3,+4+5
#3	#20 ($C_{60}H_{106}N_{12}O_{12}$)	+2,+3,+4
#3	#21 ($C_{59}H_{104}N_{12}O_{13}$)	+2,+3,+4

Compound #1 ($C_{70}H_{125}N_{13}O_{13}$) in induced EMA *fcl* KI CFCM (Sample #1)

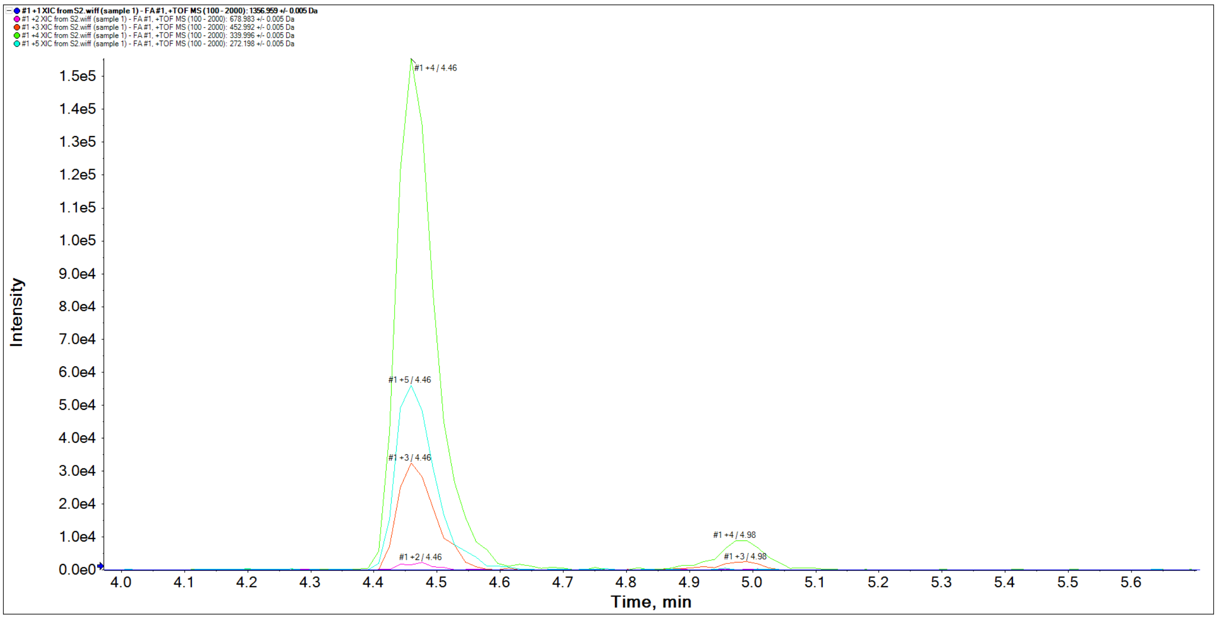

Figure S3/1. Extracted ion chromatograms (XICs) of all detected charge states of Compound #1.

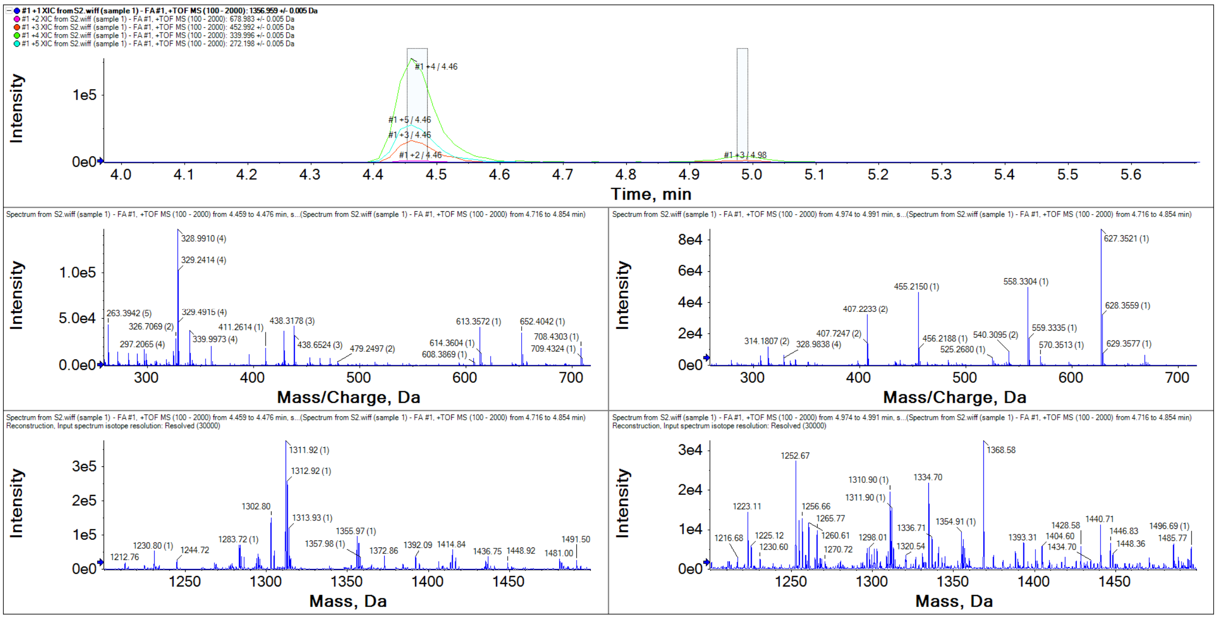

Figure S3/2. Extracted ion chromatograms (top), ESI mass spectra (middle), and reconstructed mass spectra (bottom) supporting the identification of Compound #1.

Compound #2 ($C_{67}H_{123}N_{15}O_{13}$) in induced EMA *fcl* KI CFCM (Sample #1)

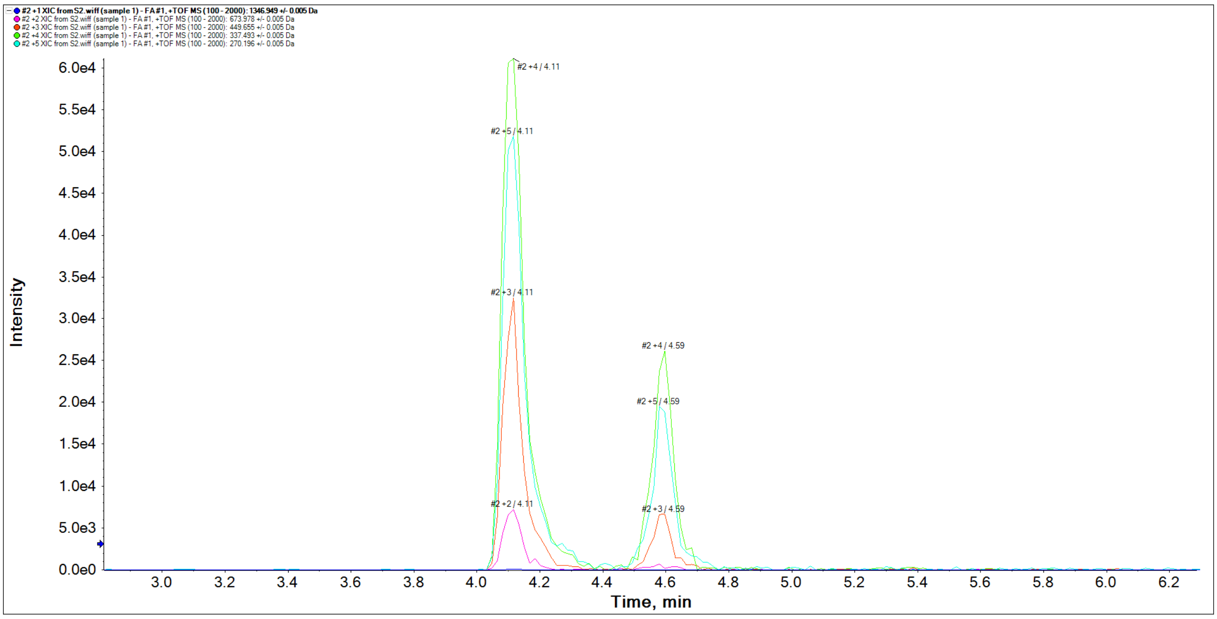

Figure S3/3. Extracted ion chromatograms (XICs) of all detected charge states of Compound #2.

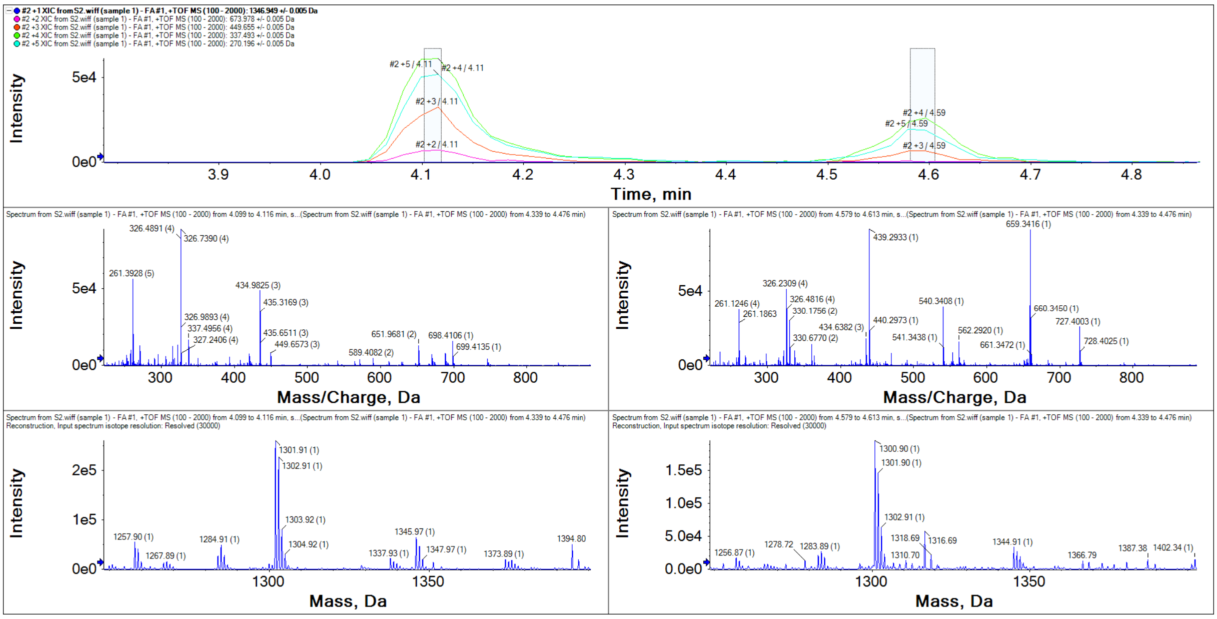

Figure S3/4. Extracted ion chromatograms (top), ESI mass spectra (middle), and reconstructed mass spectra (bottom) supporting the identification of Compound #2.

Compound #3 ($C_{68}H_{121}N_{13}O_{12}$) in induced EMA *fcl* KI CFCM (Sample #1)

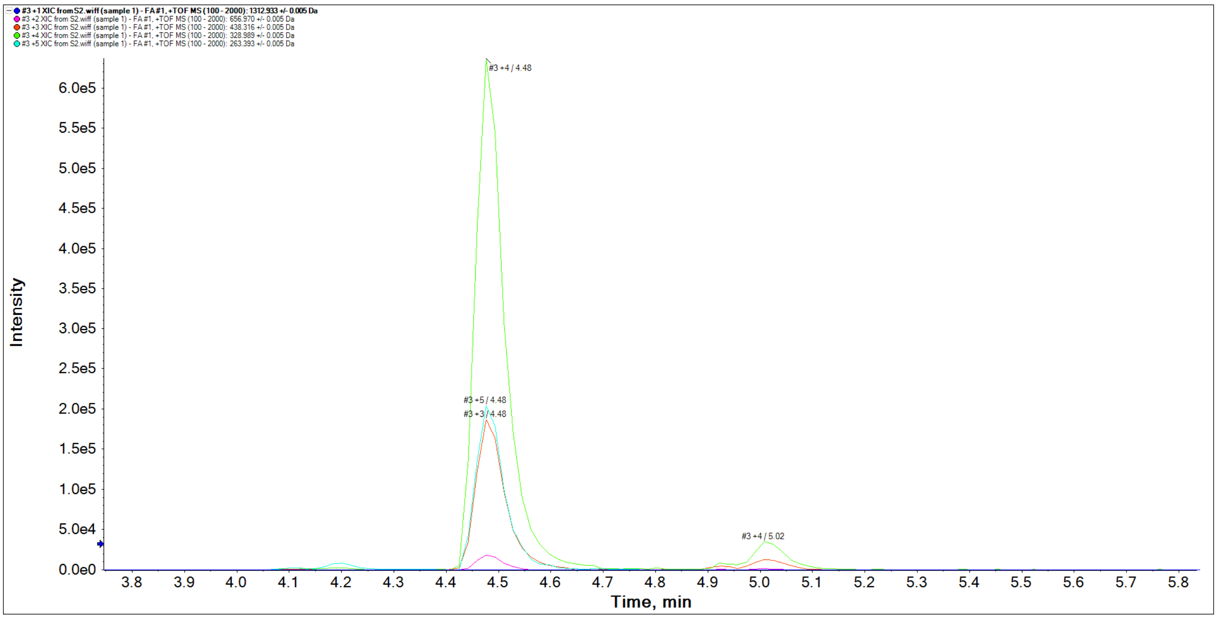

Figure S3/5. Extracted ion chromatograms (XICs) of all detected charge states of Compound #3.

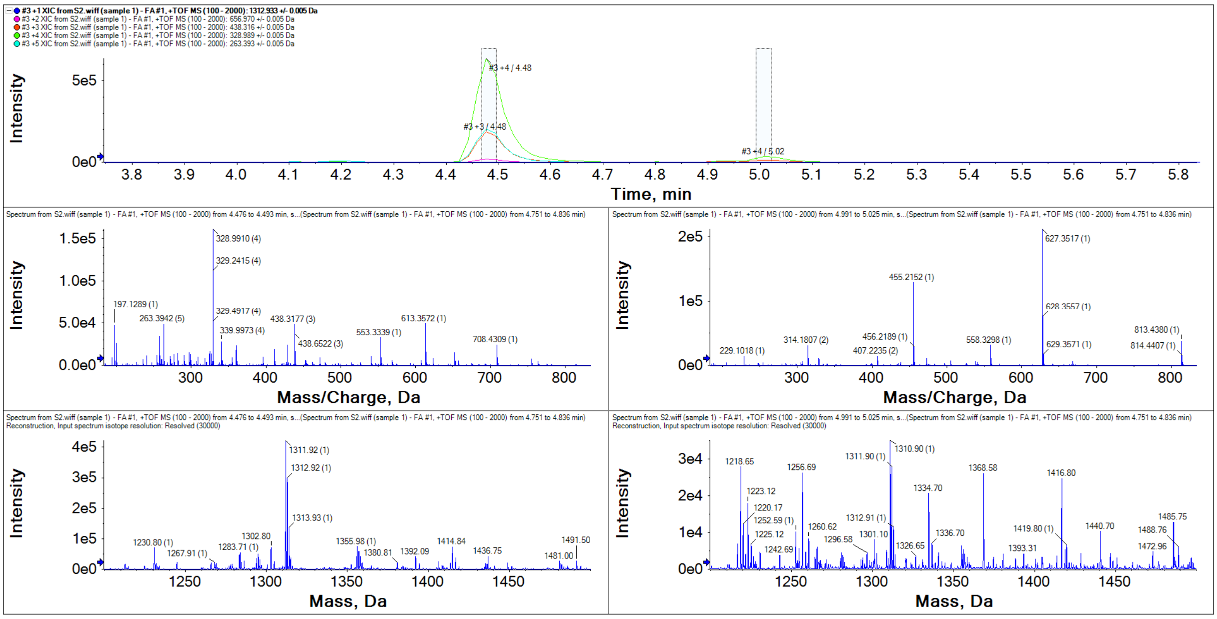

Figure S3/6. Extracted ion chromatograms (top), ESI mass spectra (middle), and reconstructed mass spectra (bottom) supporting the identification of Compound #3.

Compound #4 ($C_{65}H_{119}N_{15}O_{12}$) in induced EMA *fcl* KI CFCM (Sample #1)

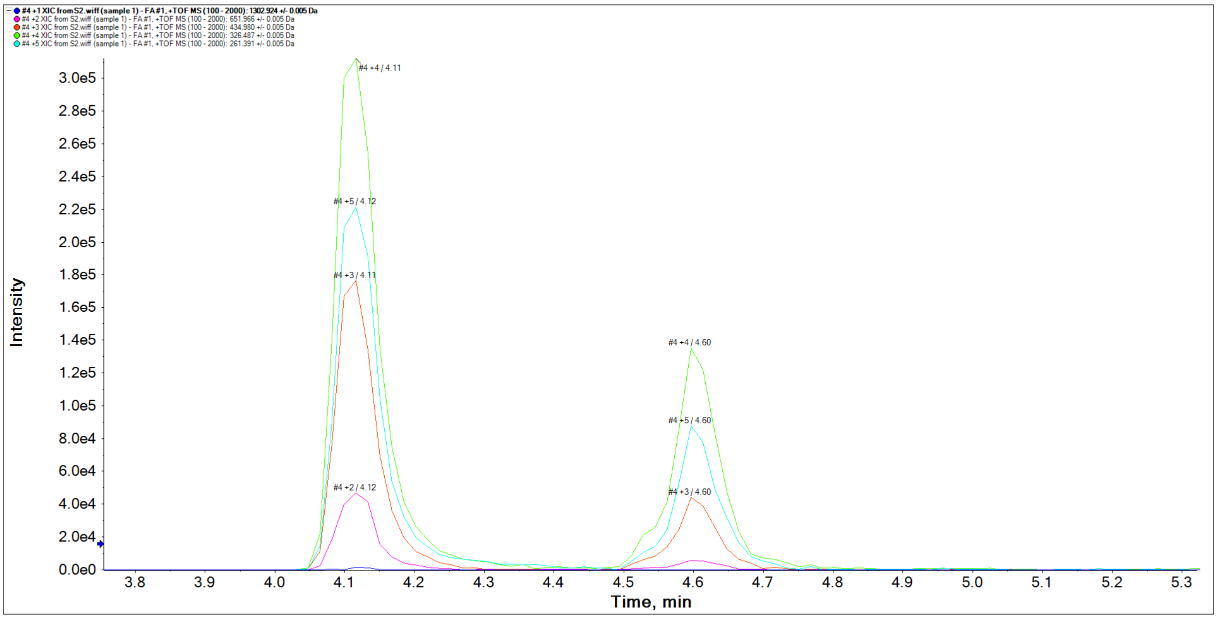

Figure S3/7. Extracted ion chromatograms (XICs) of all detected charge states of Compound #4.

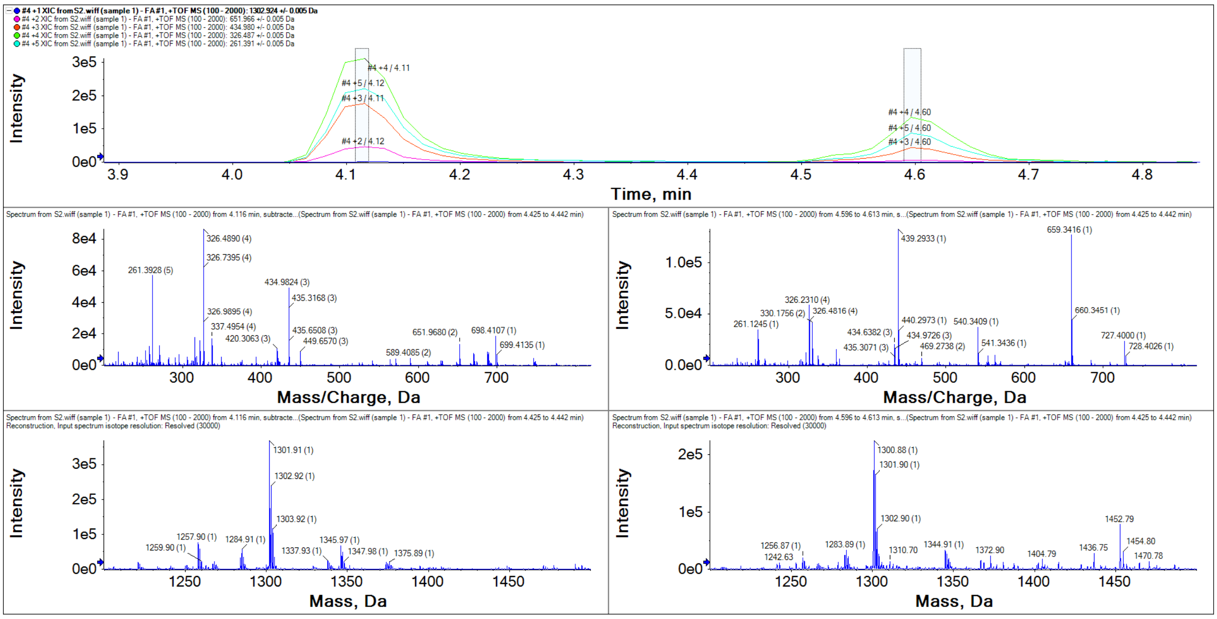

Figure S3/8. Extracted ion chromatograms (top), ESI mass spectra (middle), and reconstructed mass spectra (bottom) supporting the identification of Compound #4.

Compound #6 ($C_{59}H_{106}N_{14}O_{13}$) in induced EMA *fcl* KI CFCM (Sample #1)

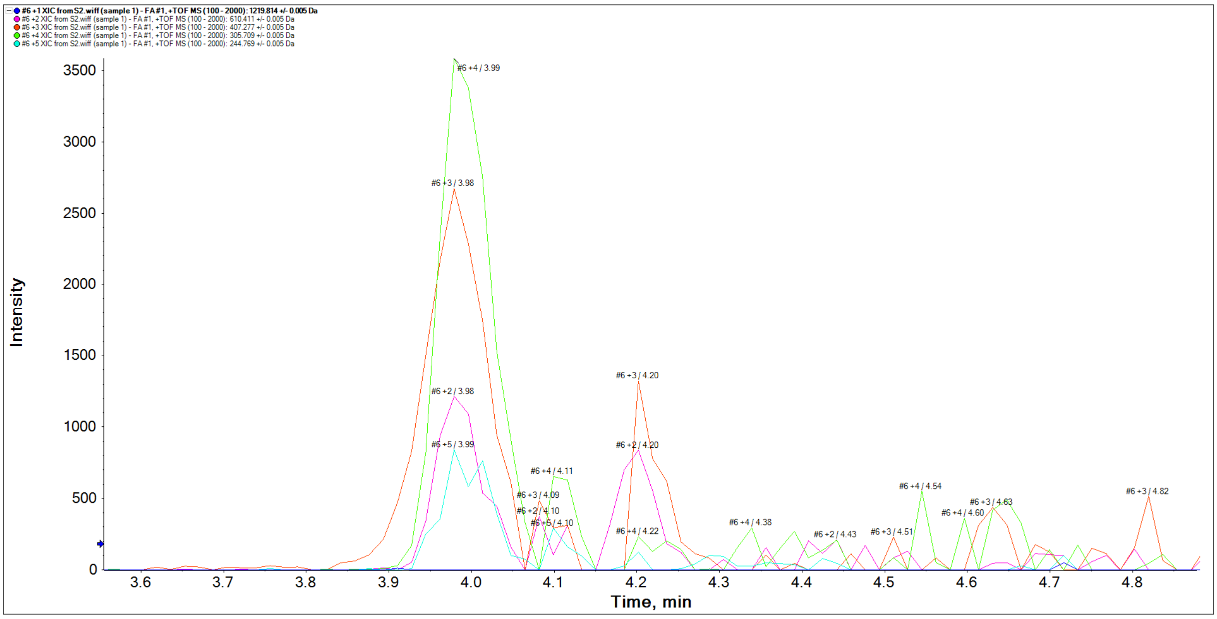

Figure S3/9. Extracted ion chromatograms (XICs) of all detected charge states Compound #6.

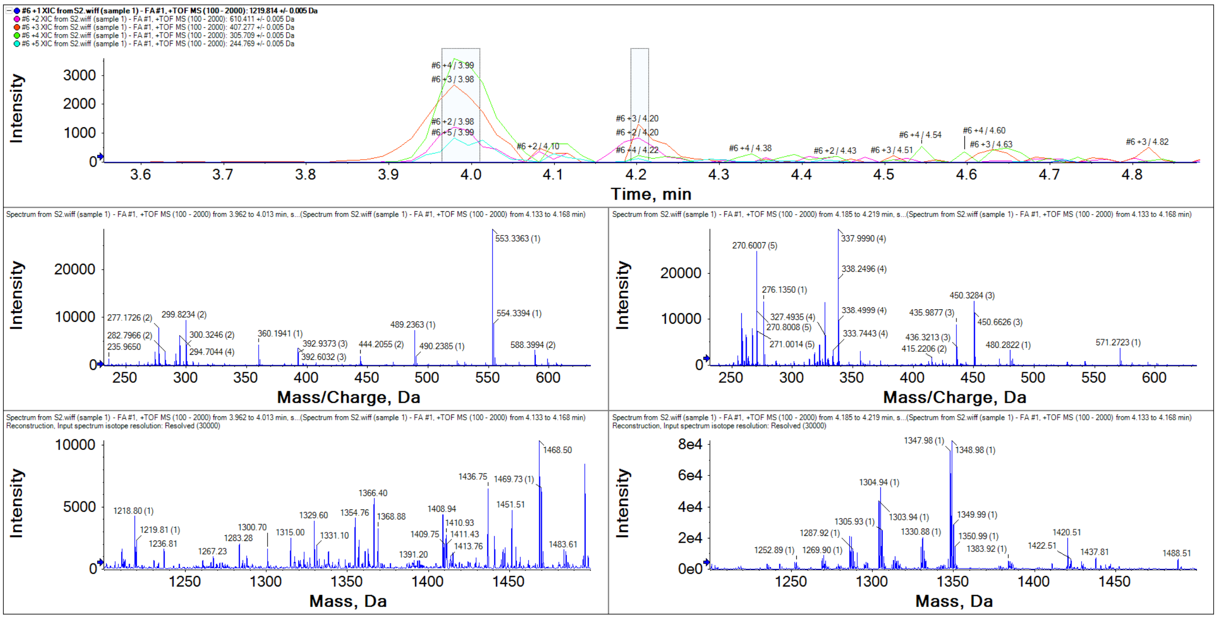

Figure S3/10. Extracted ion chromatograms (top), ESI mass spectra (middle), and reconstructed mass spectra (bottom) supporting the identification of Compound #6.

Compound #7 ($C_{60}H_{104}N_{12}O_{12}$) in induced EMA *fcl* KI CFCM (Sample #1)

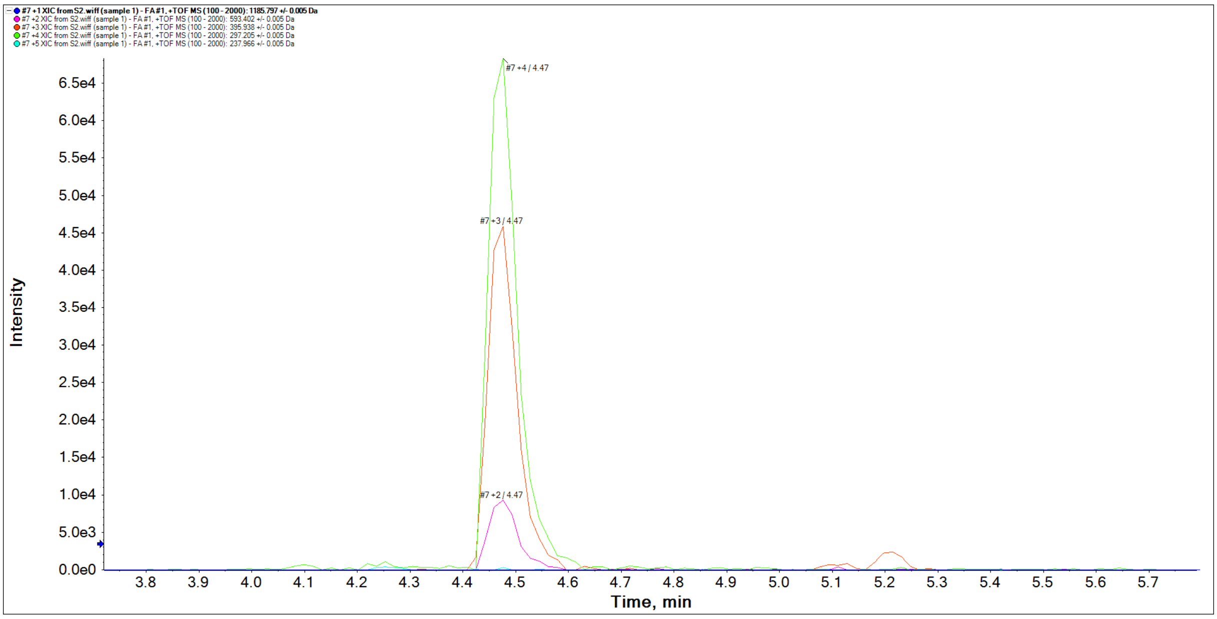

Figure S3/11. Extracted ion chromatograms (XICs) of all detected charge states Compound #7.

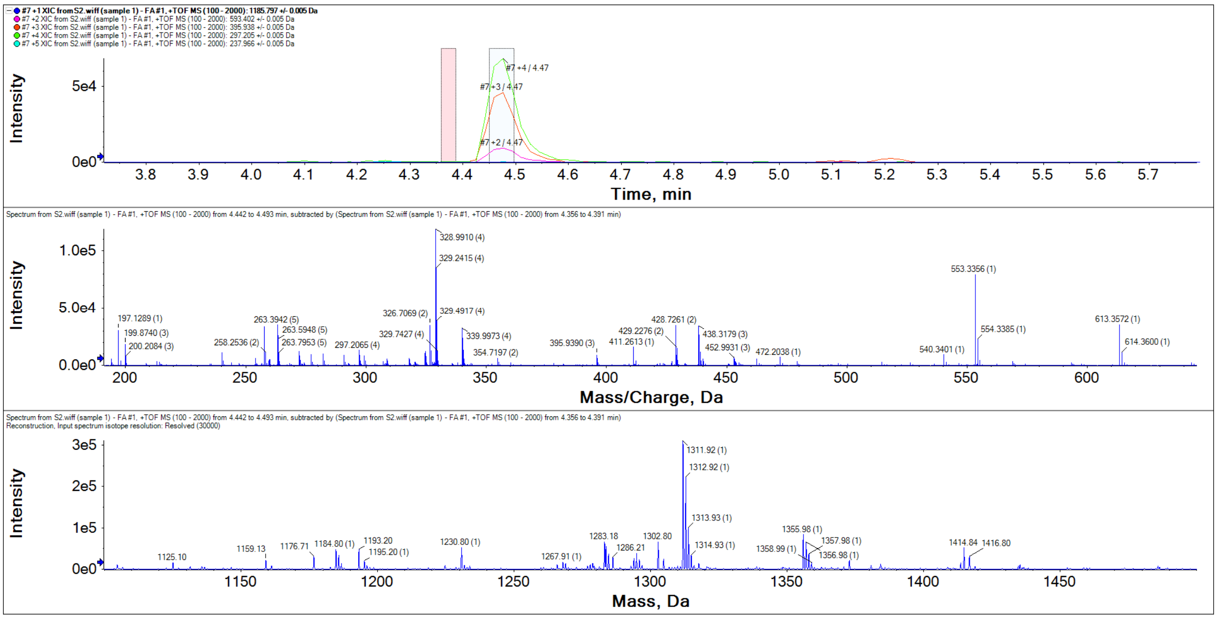

Figure S3/12. Extracted ion chromatograms (top), ESI mass spectra (middle), and reconstructed mass spectra (bottom) supporting the identification of Compound #7.

Compound #8 ($C_{57}H_{102}N_{14}O_{12}$) in induced EMA *fcl* KI CFCM (Sample #1)

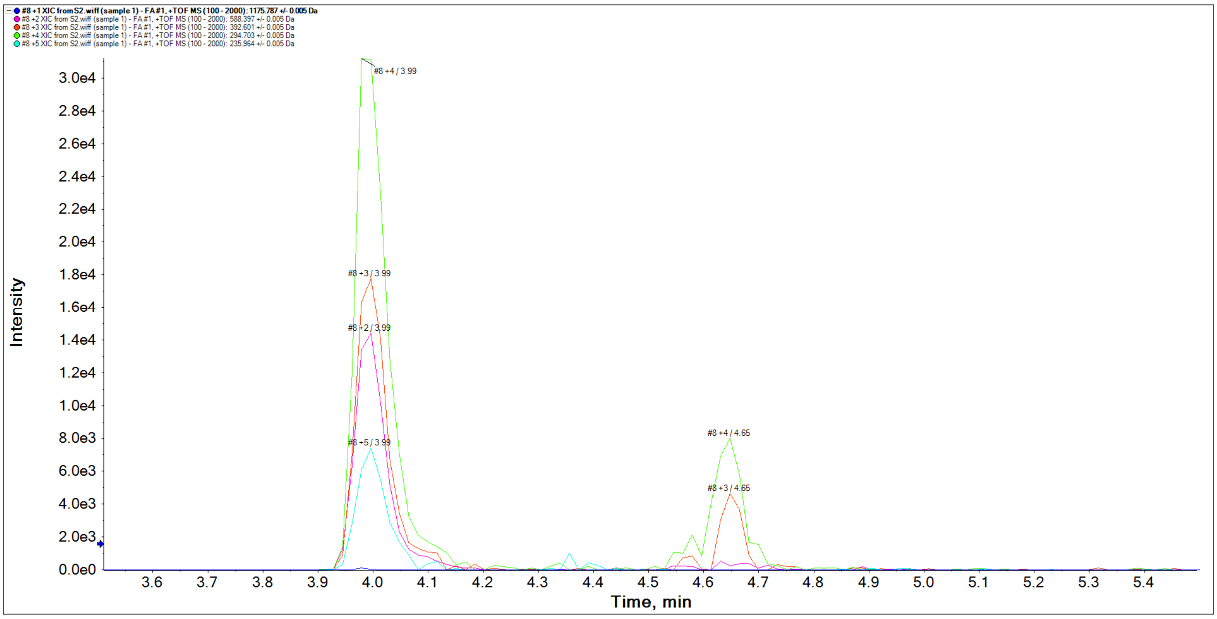

Figure S3/13. Extracted ion chromatograms (XICs) of all detected charge states of Compound #8.

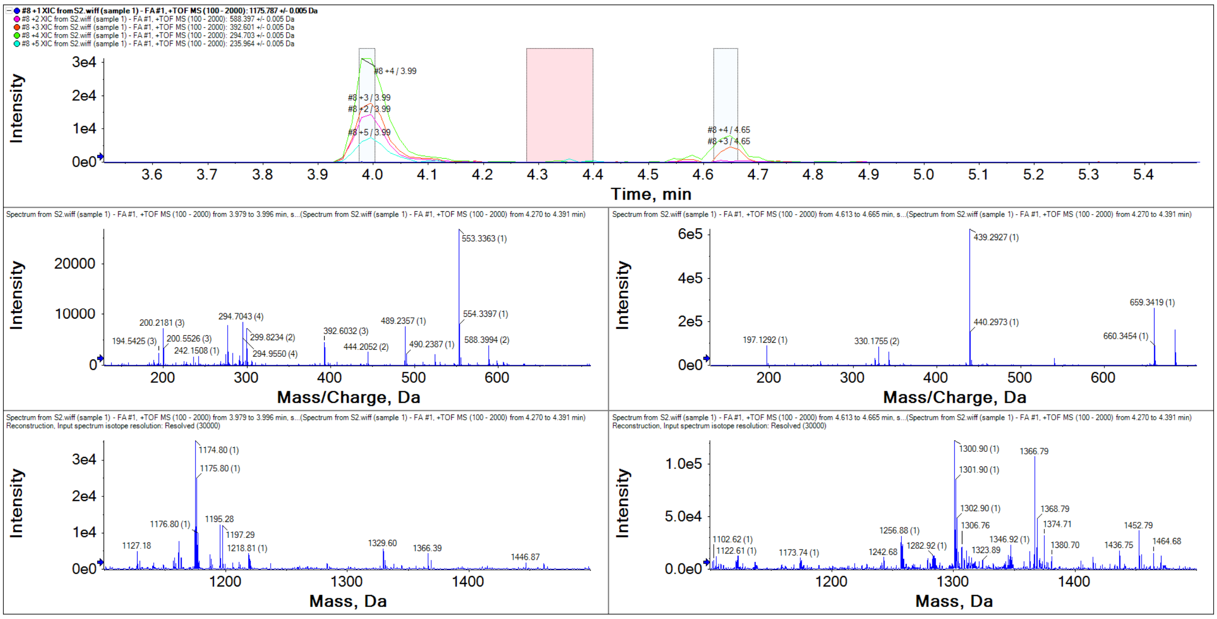

Figure 4/14. Extracted ion chromatograms (top), ESI mass spectra (middle), and reconstructed mass spectra (bottom) supporting the identification of Compound #8.

Compound #17 ($C_{57}H_{104}N_{14}O_{12}$) in induced EMA *fcl* KI CFCM (Sample #1)

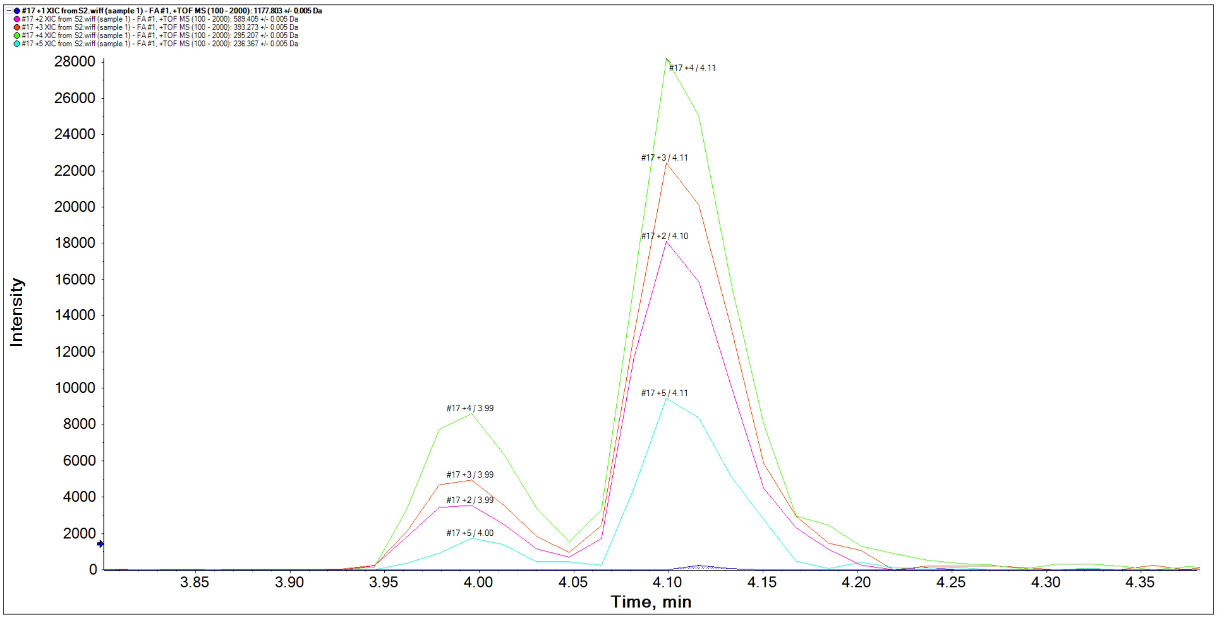

Figure S3/15. Extracted ion chromatograms (XICs) of all detected charge states of Compound #17.

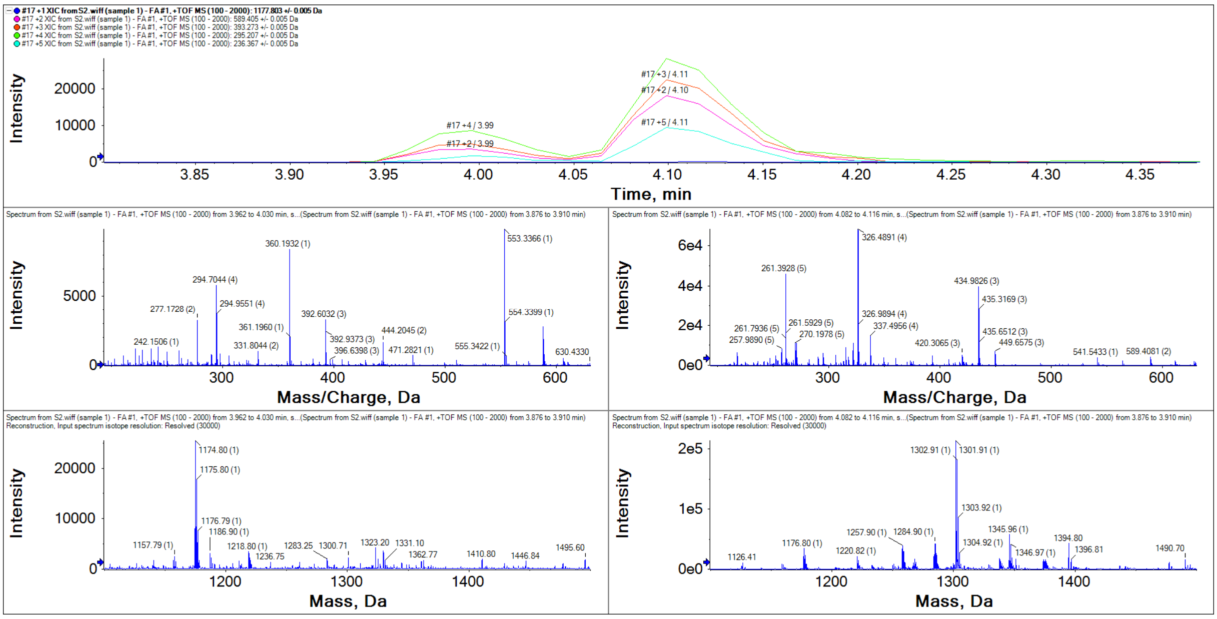

Figure S3/16. Extracted ion chromatograms (top), ESI mass spectra (middle), and reconstructed mass spectra (bottom) supporting the identification of Compound #17.

Compound #18 ($C_{56}H_{102}N_{14}O_{13}$) in induced EMA *fcl* KI CFCM (Sample #1)

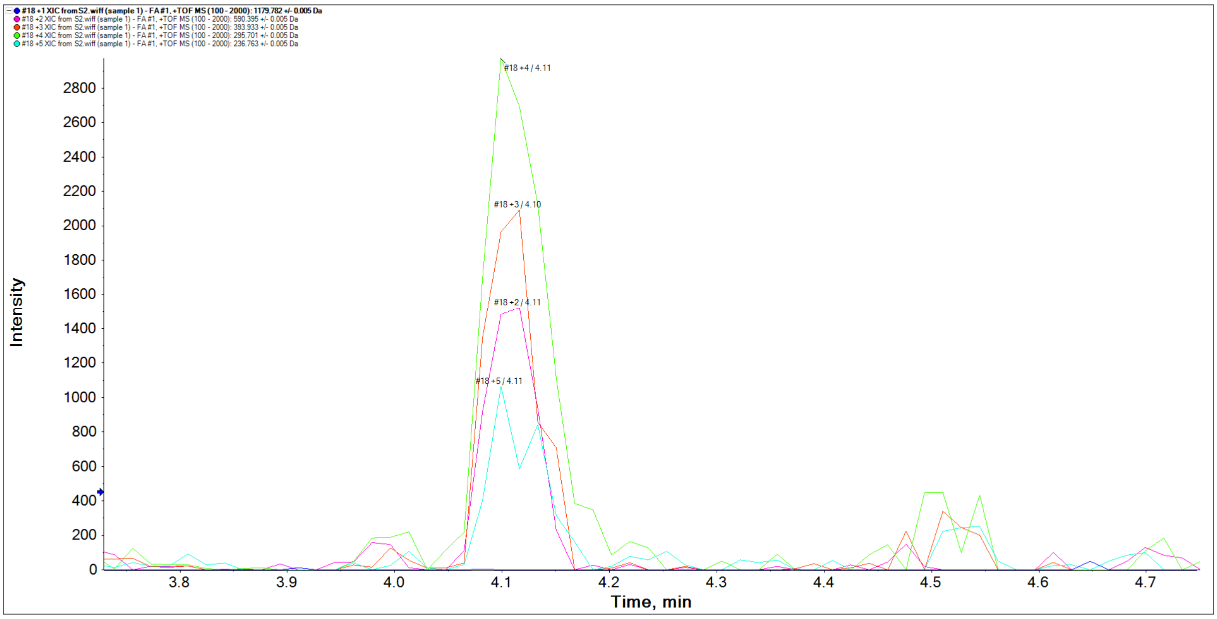

Figure S3/17. Extracted ion chromatograms (XICs) of all detected charge states of Compound #18.

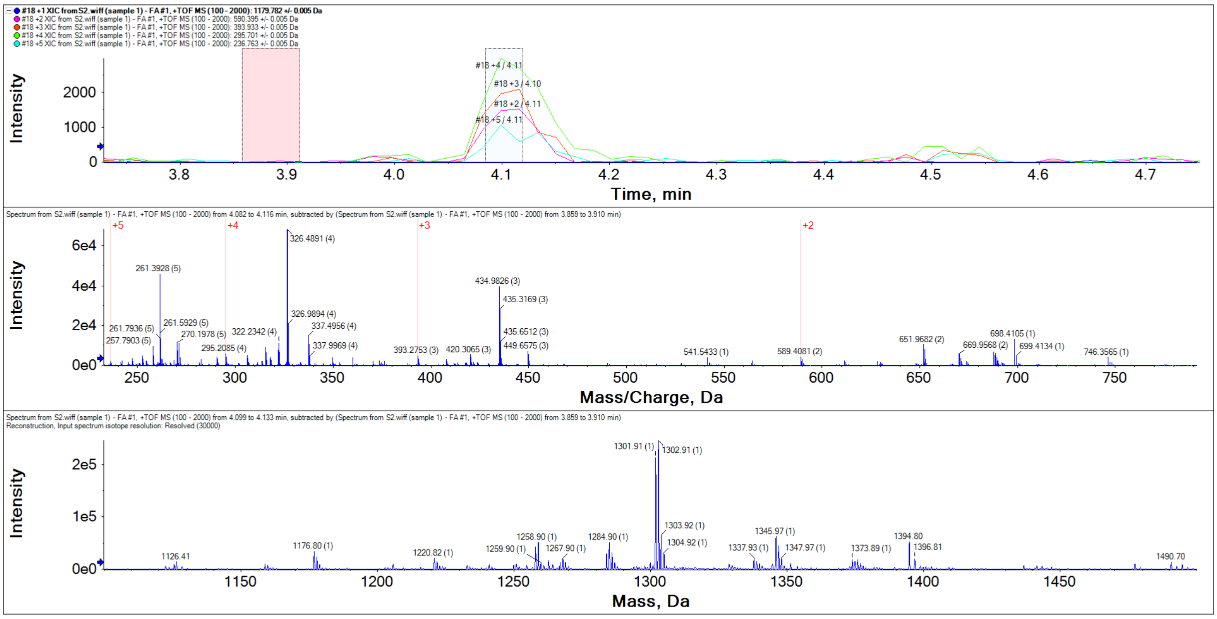

Figure S3/18. Extracted ion chromatograms (top), ESI mass spectra (middle), and reconstructed mass spectra (bottom) supporting the identification of Compound #18.

Compound #19 ($C_{59}H_{108}N_{14}O_{13}$) in induced EMA *fcl* KI CFCM (Sample #1)

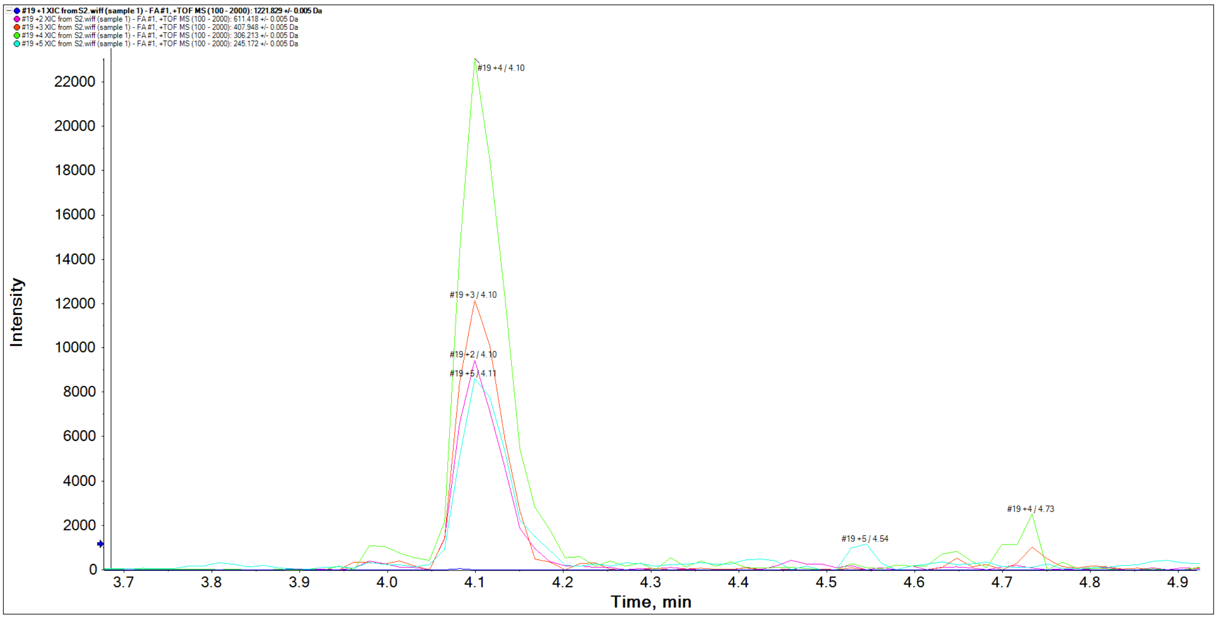

Figure S3/19. Extracted ion chromatograms (XICs) of all detected charge states of Compound #19.

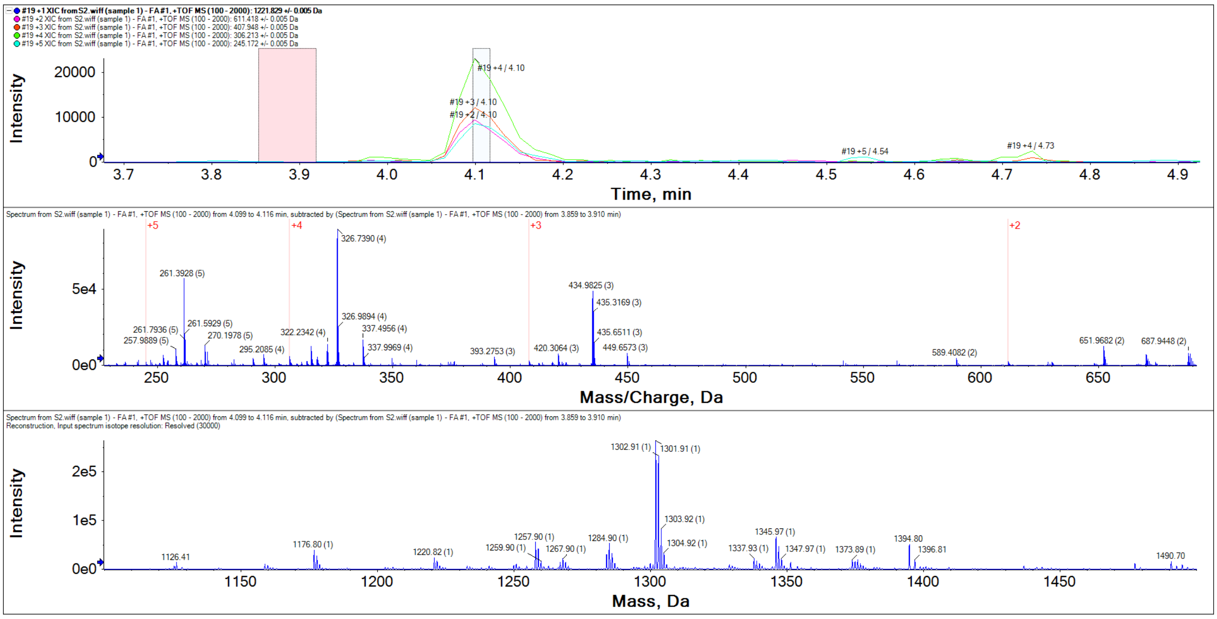

Figure S3/20. Extracted ion chromatograms (top), ESI mass spectra (middle), and reconstructed mass spectra (bottom) supporting the identification of Compound #19.

Compound #22 ($C_{62}H_{110}N_{12}O_{13}$) in induced EMA *fcl* KI CFCM (Sample #1)

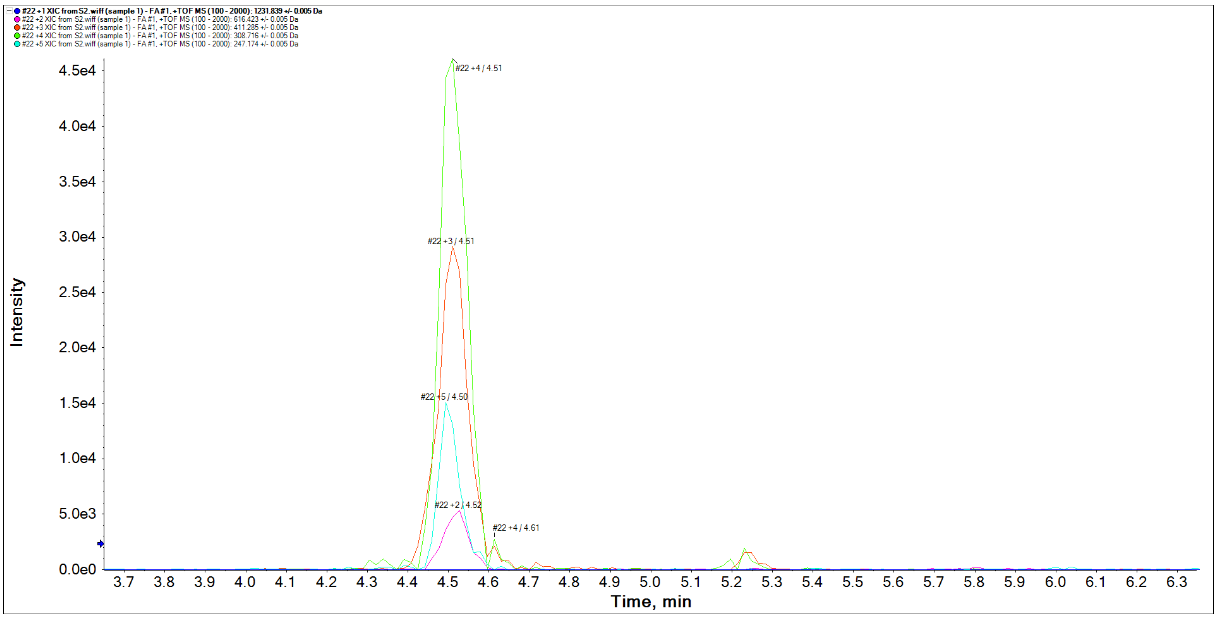

Figure S3/21. Extracted ion chromatograms (XICs) of all detected charge states of Compound #22.

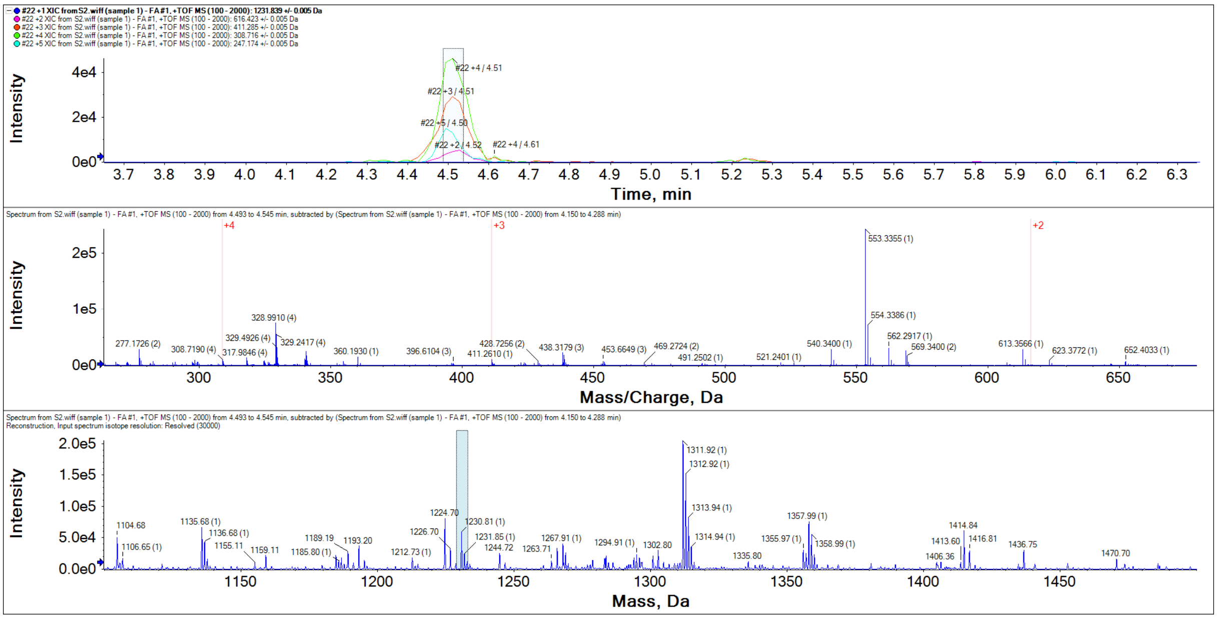

Figure S3/22. Extracted ion chromatograms (top), ESI mass spectra (middle), and reconstructed mass spectra (bottom) supporting the identification of Compound #22.

Compound #23 ($C_{68}H_{123}N_{13}O_{12}$) in induced EMA *fcl* KI CFCM (Sample #1)

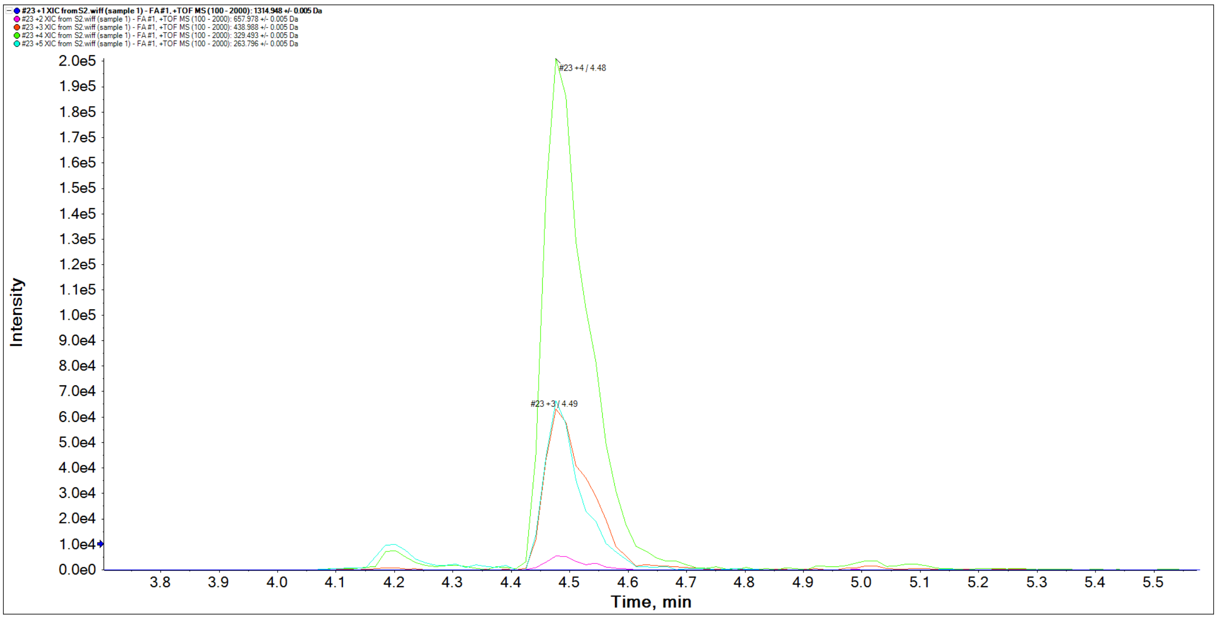

Figure S3/23. Extracted ion chromatograms (XICs) of all detected charge states of Compound #23.

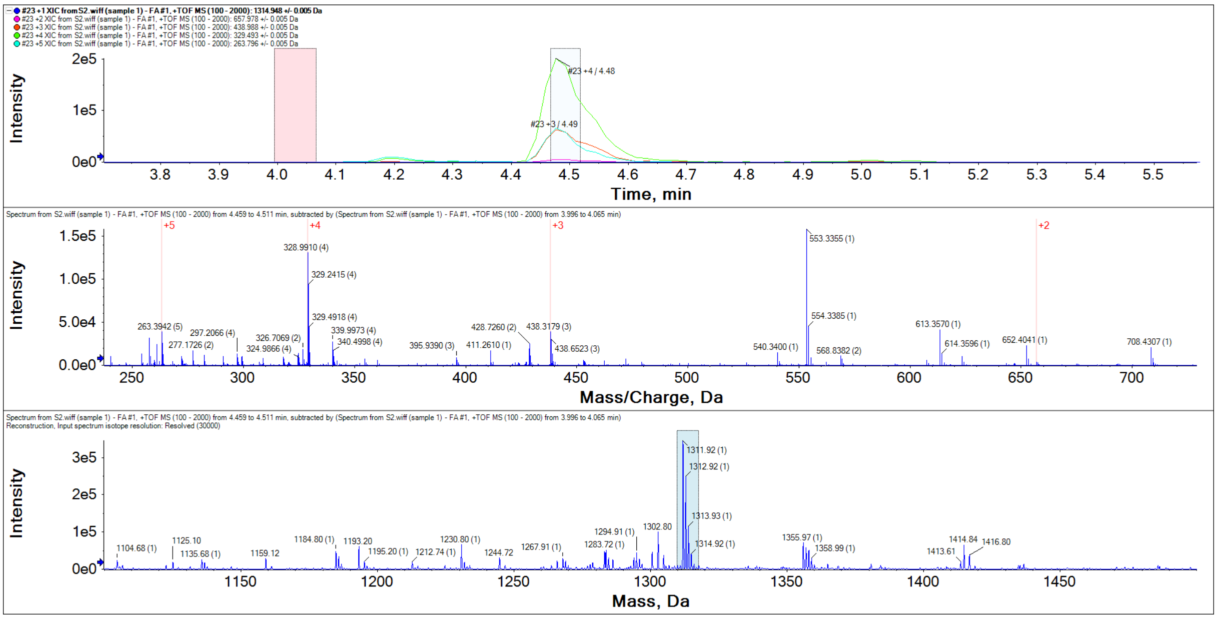

Figure S3/24. Extracted ion chromatograms (top), ESI mass spectra (middle), and reconstructed mass spectra (bottom) supporting the identification of Compound #23.

Compound #25 ($C_{65}H_{121}N_{15}O_{12}$) in induced EMA *fcl* KI CFCM (Sample #1)

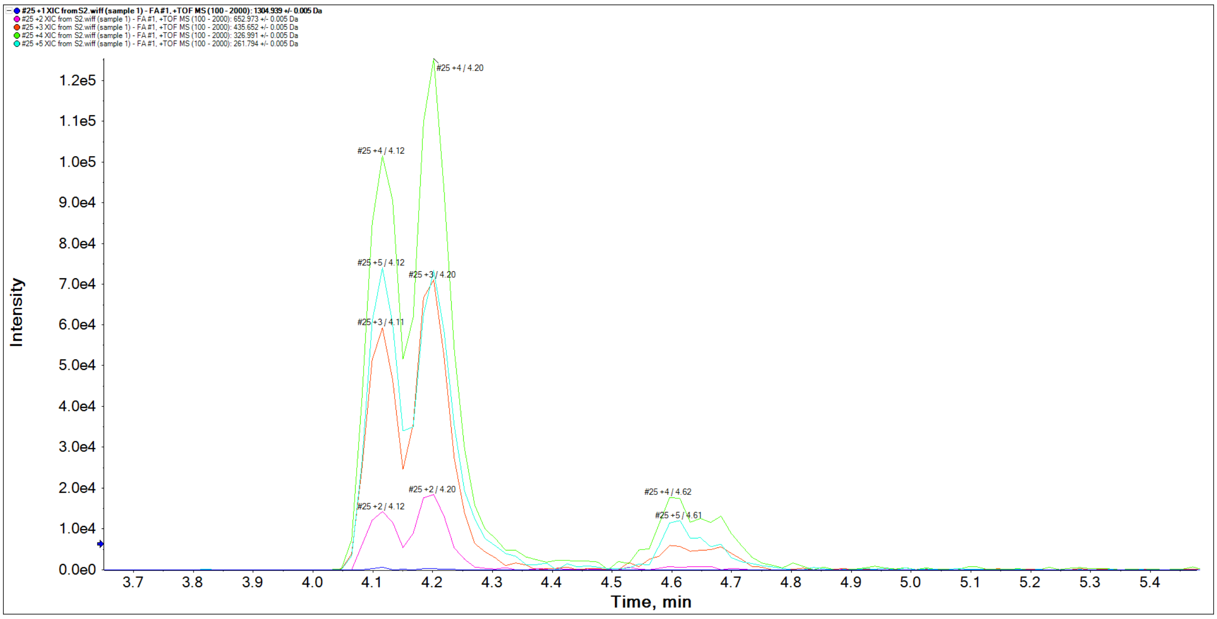

Figure S3/25. Extracted ion chromatograms (XICs) of all detected charge states of Compound #25.

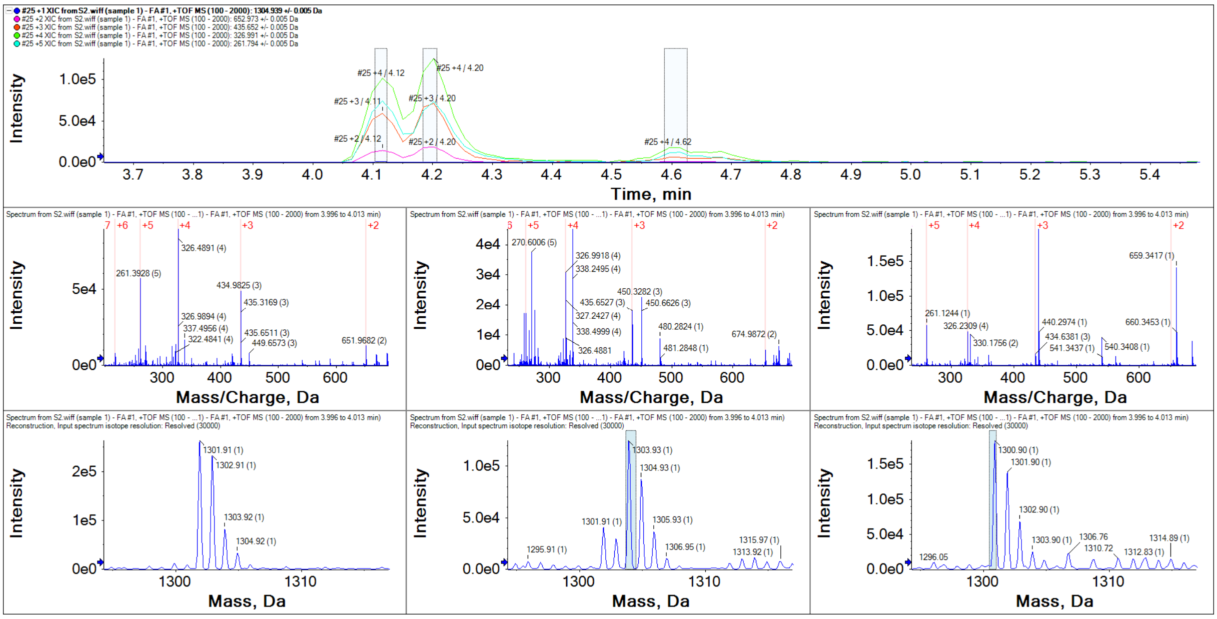

Figure S3/26. Extracted ion chromatograms (top), ESI mass spectra (middle), and reconstructed mass spectra (bottom) supporting the identification of Compound #25.

Compound #27 ($C_{70}H_{127}N_{13}O_{13}$) in induced EMA *fcl* KI CFCM (Sample #1)

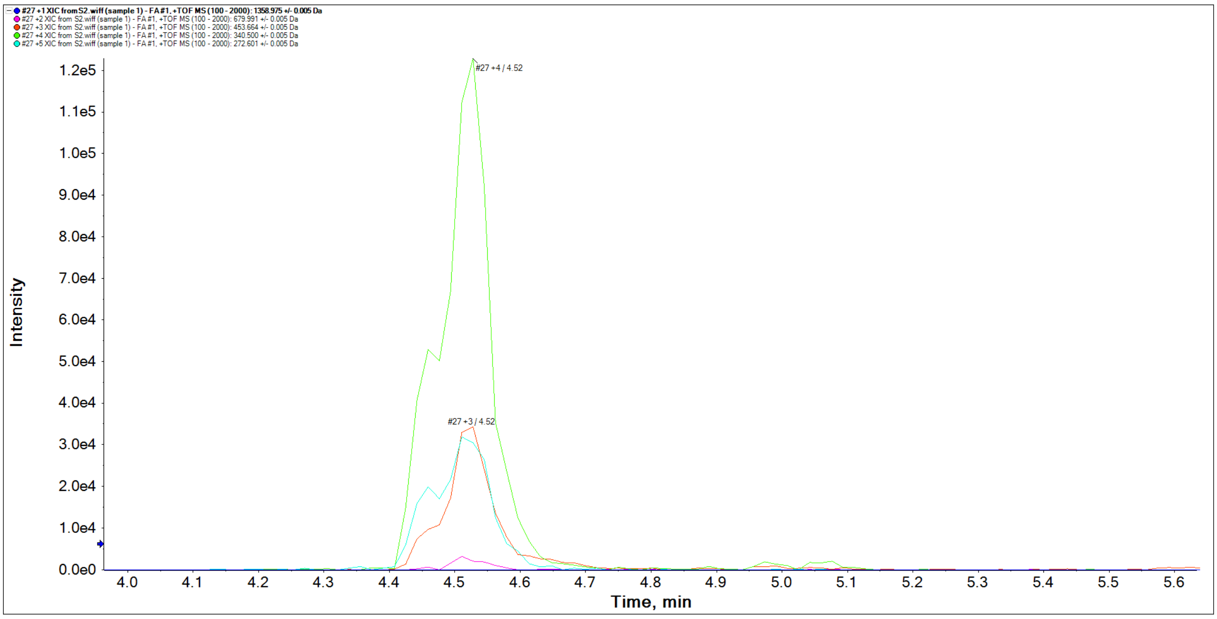

Figure S3/27. Extracted ion chromatograms (XICs) of all detected charge states of Compound #27.

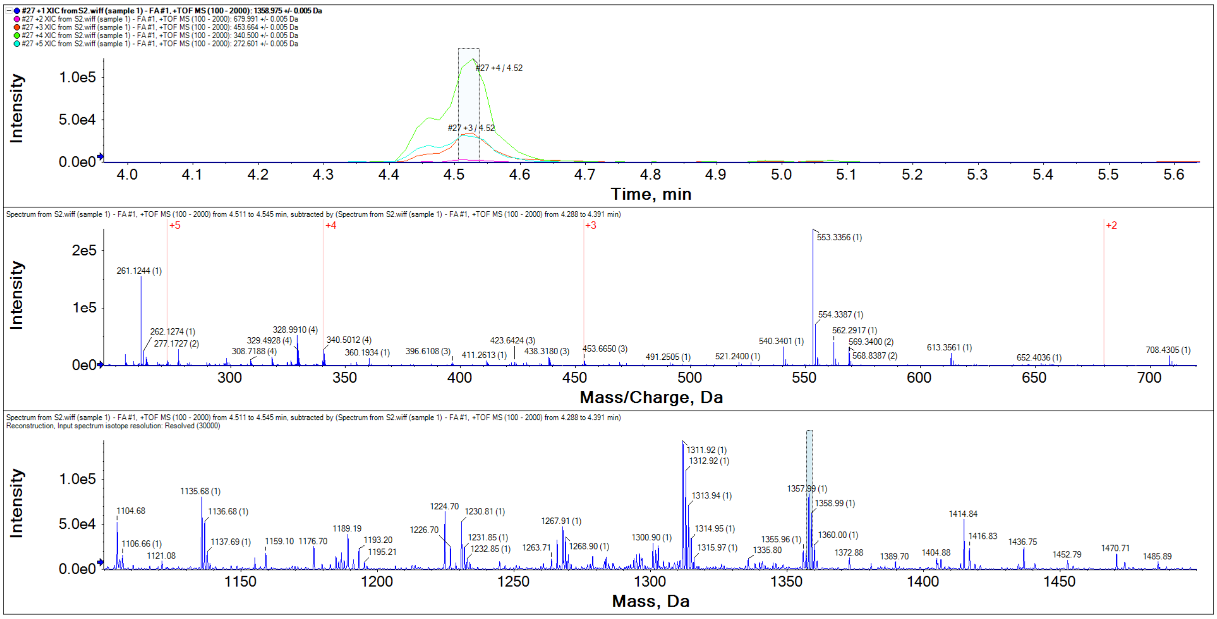

Figure S3/28. Extracted ion chromatograms (top), ESI mass spectra (middle), and reconstructed mass spectra (bottom) supporting the identification of Compound #27.

Compound #29 ($C_{67}H_{125}N_{15}O_{13}$) in induced EMA *fcl* KI CFCM (Sample #1)

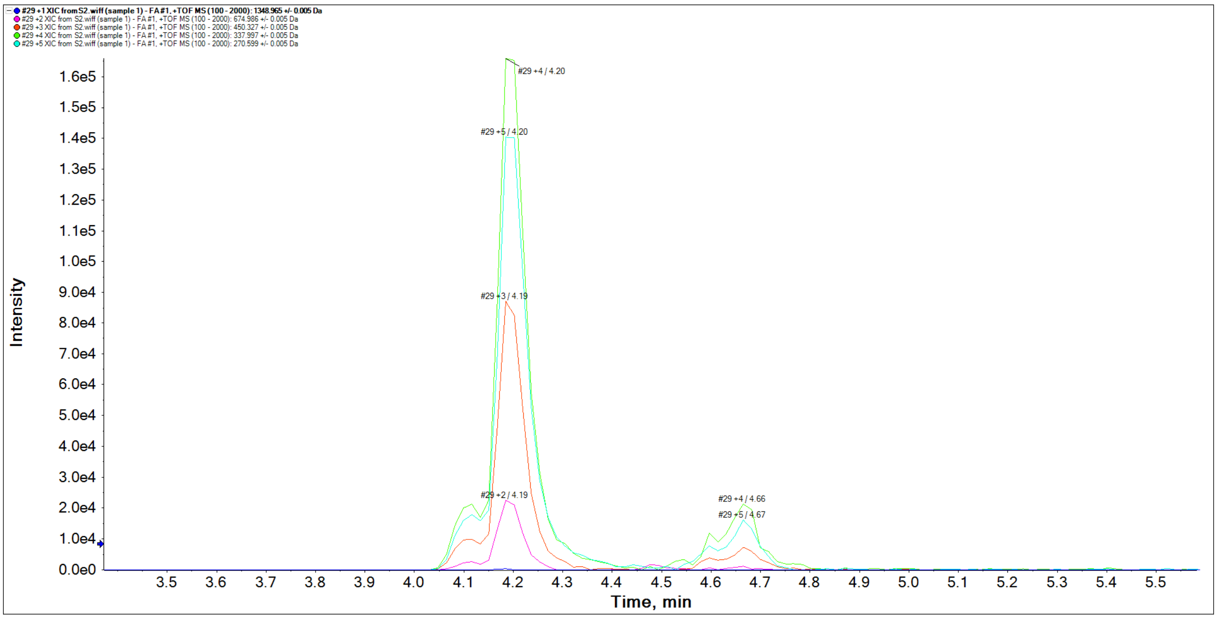

Figure S3/29. Extracted ion chromatograms (XICs) of all detected charge states of Compound #29.

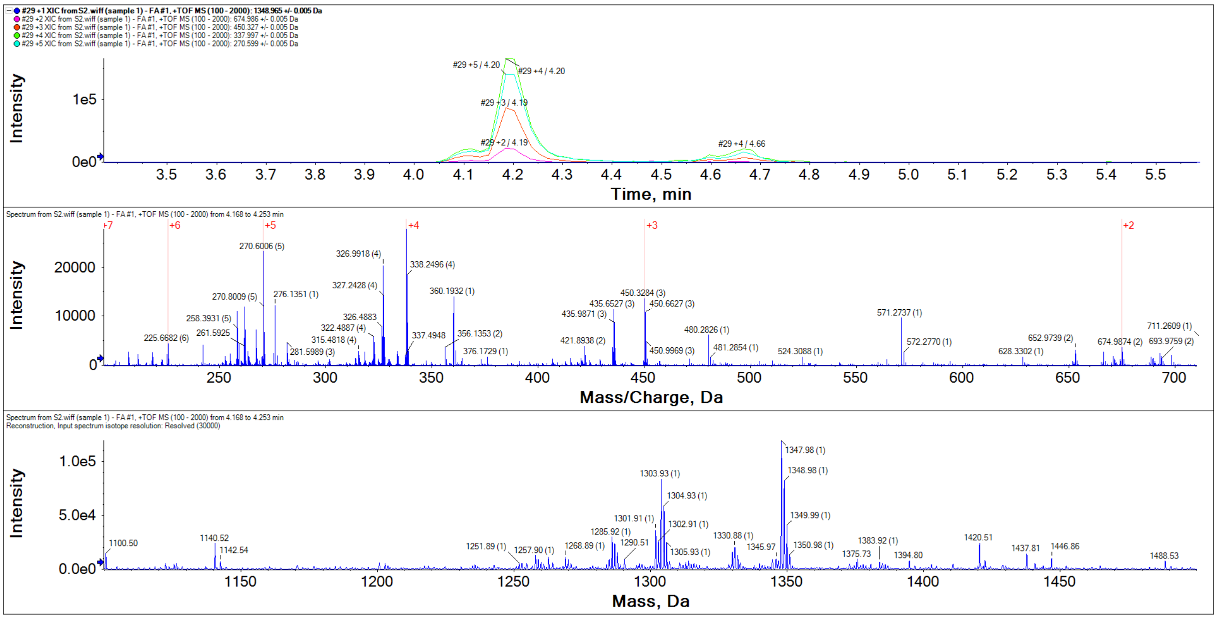

Figure S3/30. Extracted ion chromatograms (top), ESI mass spectra (middle), and reconstructed mass spectra (bottom) supporting the identification of Compound #29.

Compound #30 ($C_{66}H_{123}N_{15}O_{14}$) in induced EMA *fcl* KI CFCM (Sample #1)

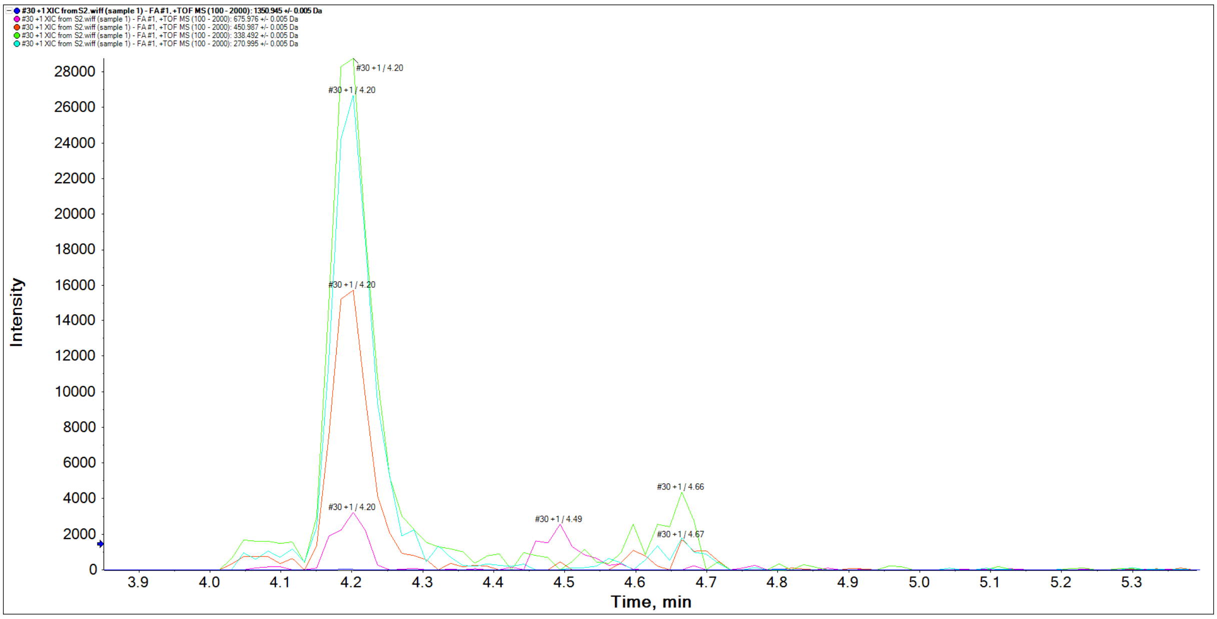

Figure S3/31. Extracted ion chromatograms (XICs) of all detected charge states of Compound #30.

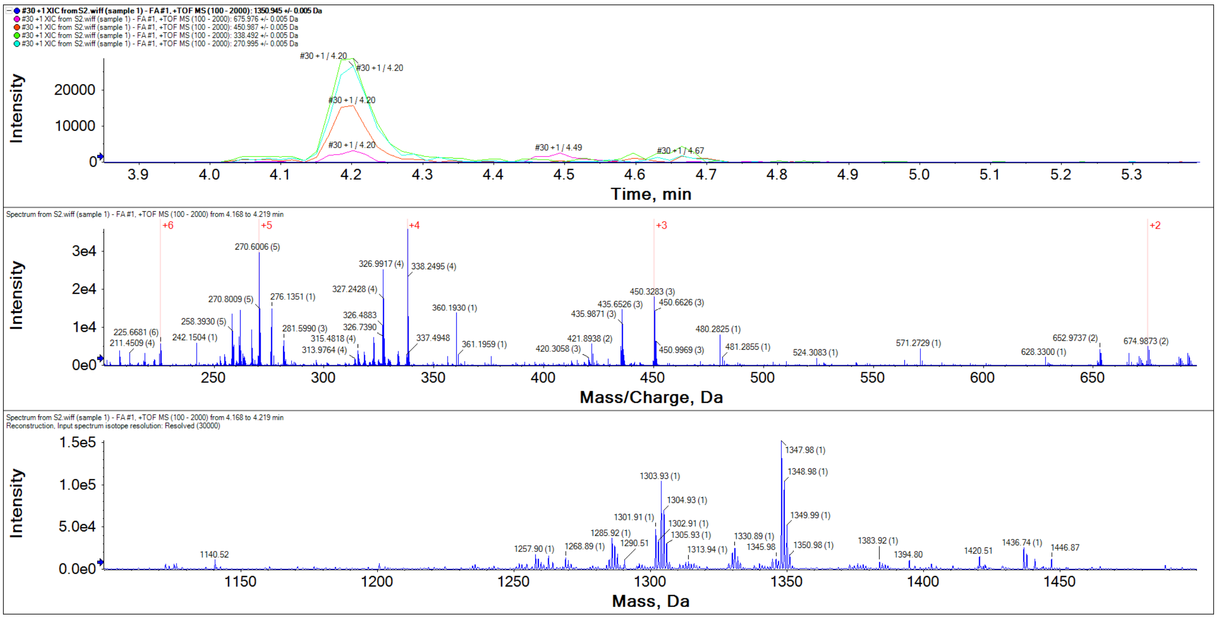

Figure S3/32. Extracted ion chromatograms (top), ESI mass spectra (middle), and reconstructed mass spectra (bottom) supporting the identification of Compound #30.

Compound #15 ($C_{54}H_{102}N_{12}O_{12}$) in induced EMC *fcl* KI CFCM (Sample #3)

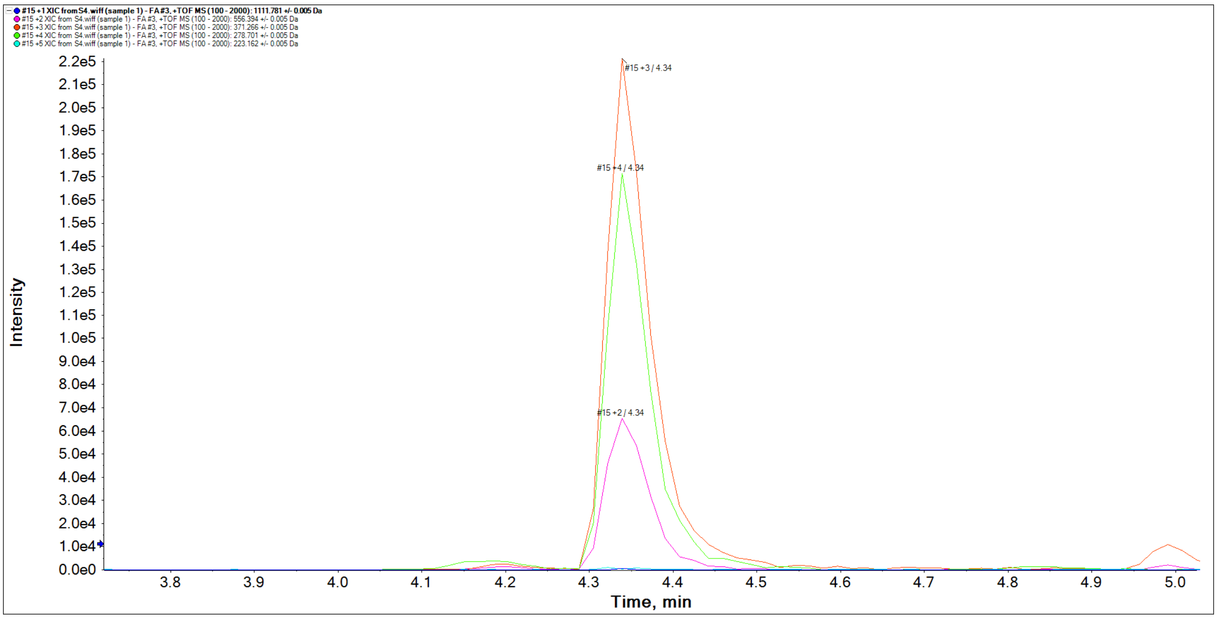

Figure 4/33. Extracted ion chromatograms (XICs) of all detected charge states of Compound #15.

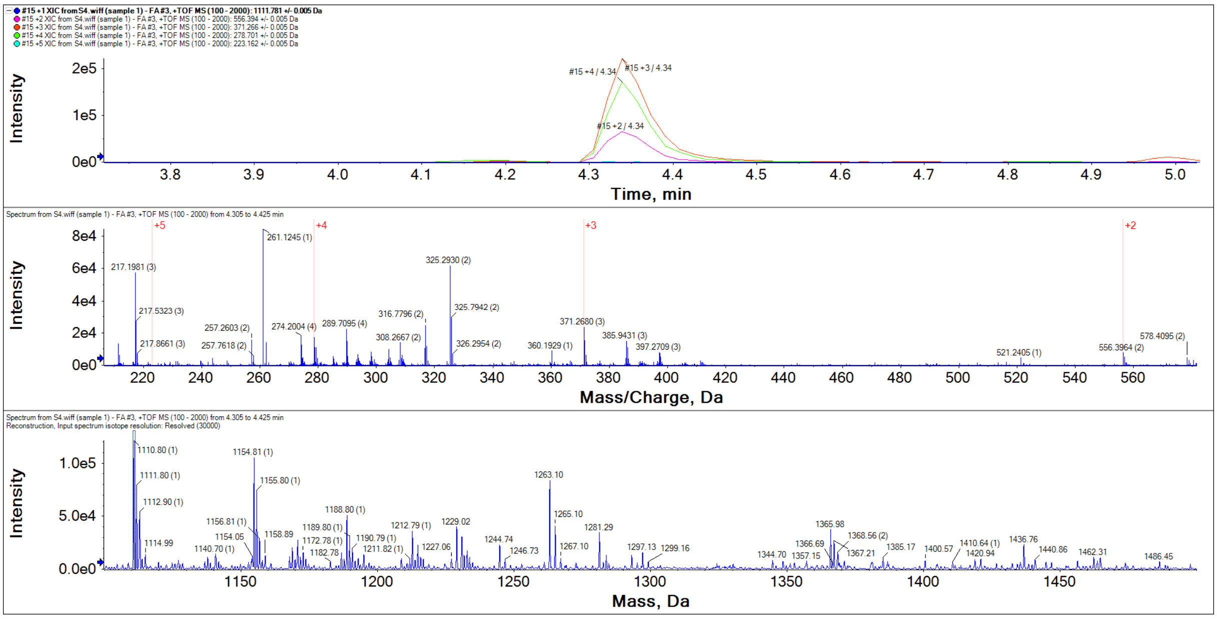

Figure S3/34. Extracted ion chromatograms (top), ESI mass spectra (middle), and reconstructed mass spectra (bottom) supporting the identification of Compound #15.

Compound #16 ($C_{53}H_{100}N_{12}O_{13}$) in induced EMC *fcl* KI CFCM (Sample #3)

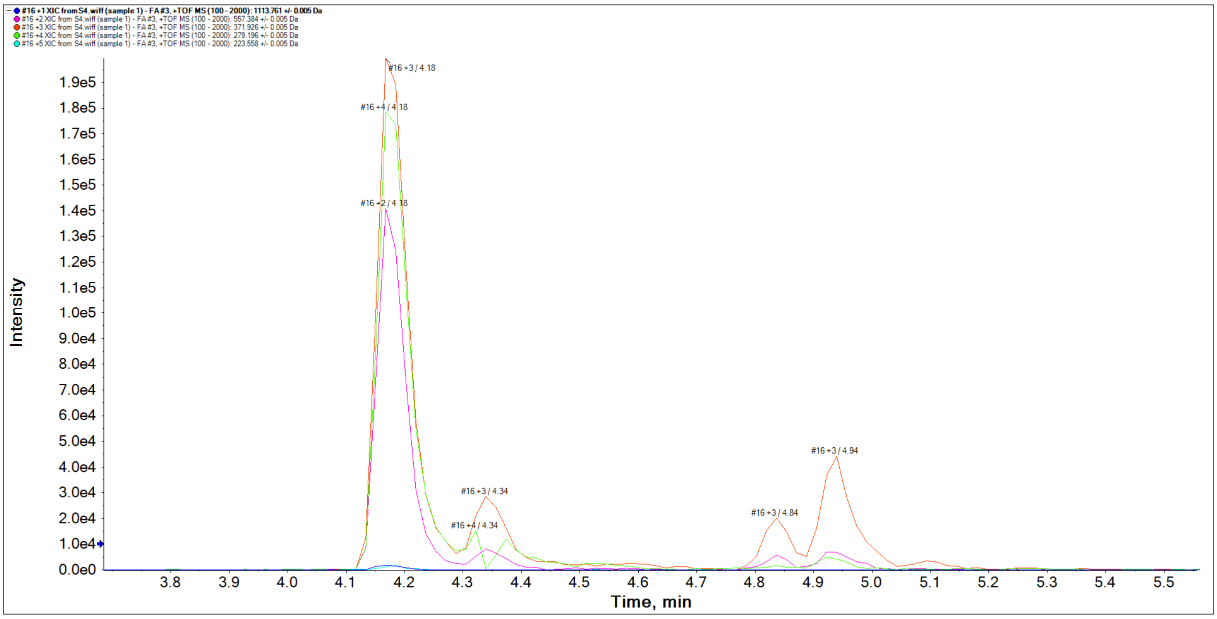

Figure S3/35. Extracted ion chromatograms (XICs) of all detected charge states of Compound #16.

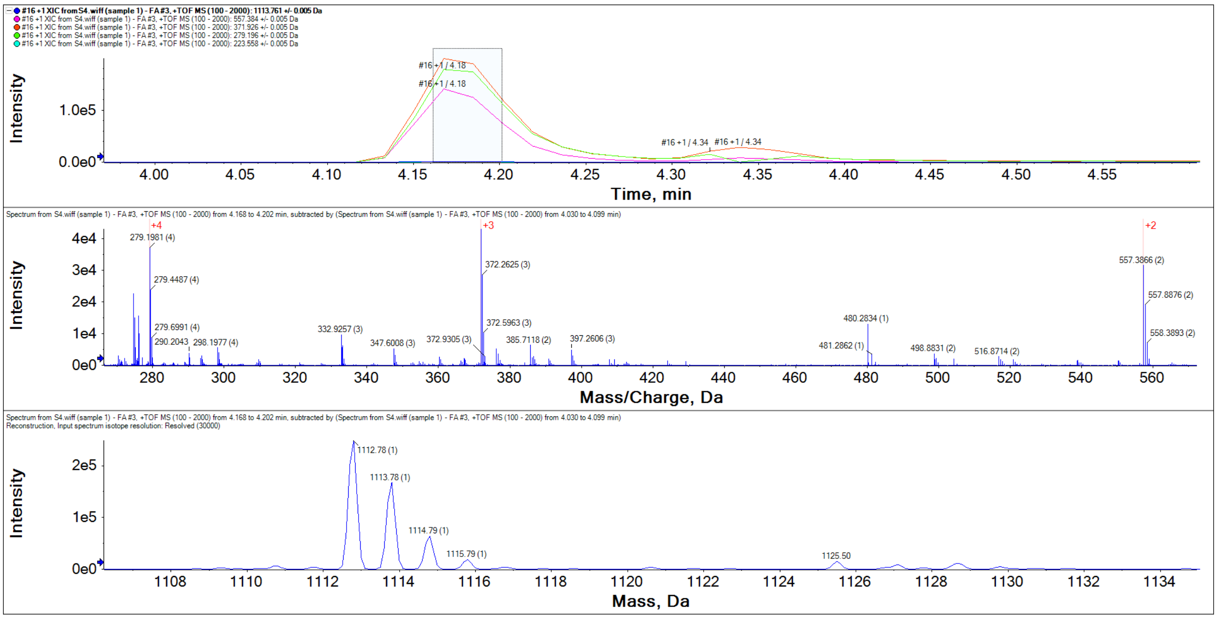

Figure S3/36. Extracted ion chromatograms (top), ESI mass spectra (middle), and reconstructed mass spectra (bottom) supporting the identification of Compound #16.

Compound #17 ($C_{57}H_{104}N_{14}O_{12}$) in induced EMC *fcl* KI CFCM (Sample #3)

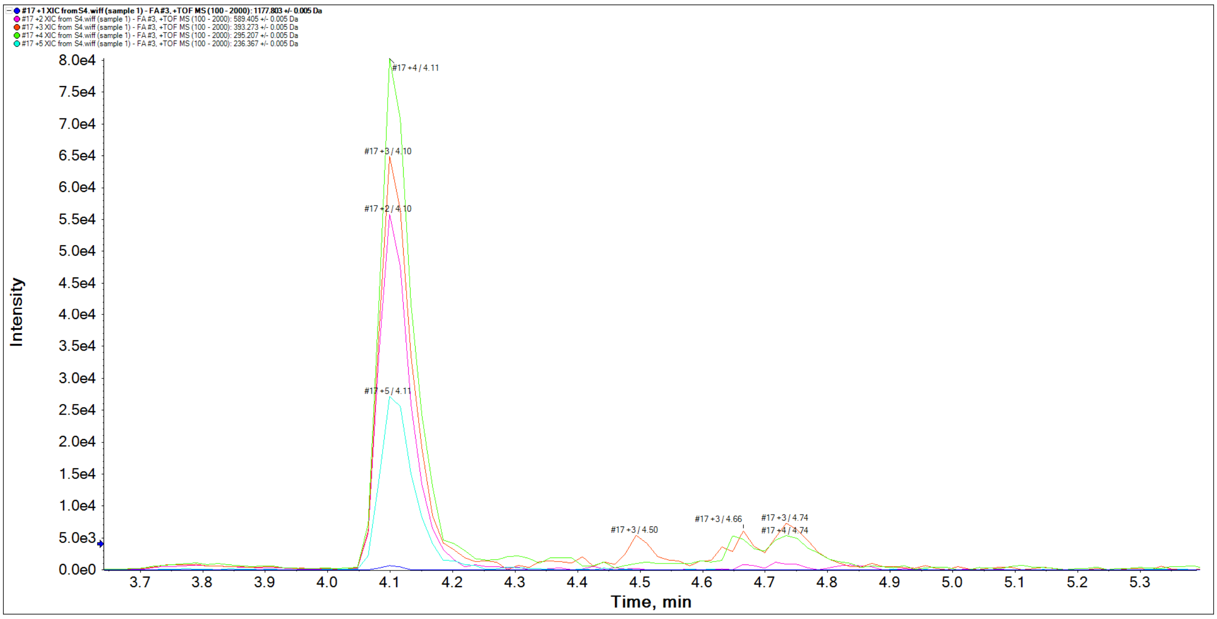

Figure S3/37. Extracted ion chromatograms (XICs) of all detected charge states of Compound #17.

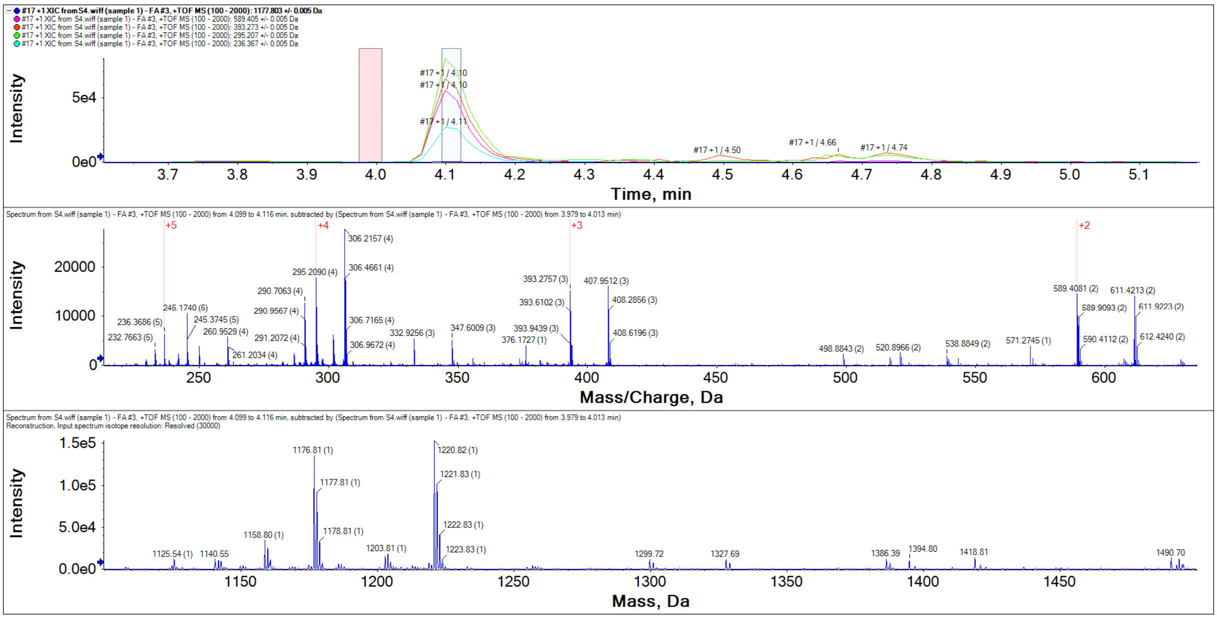

Figure S3/38. Extracted ion chromatograms (top), ESI mass spectra (middle), and reconstructed mass spectra (bottom) supporting the identification of Compound #17.

Compound #18 ($C_{56}H_{102}N_{14}O_{13}$) in induced EMC *fcl* KI CFCM (Sample #3)

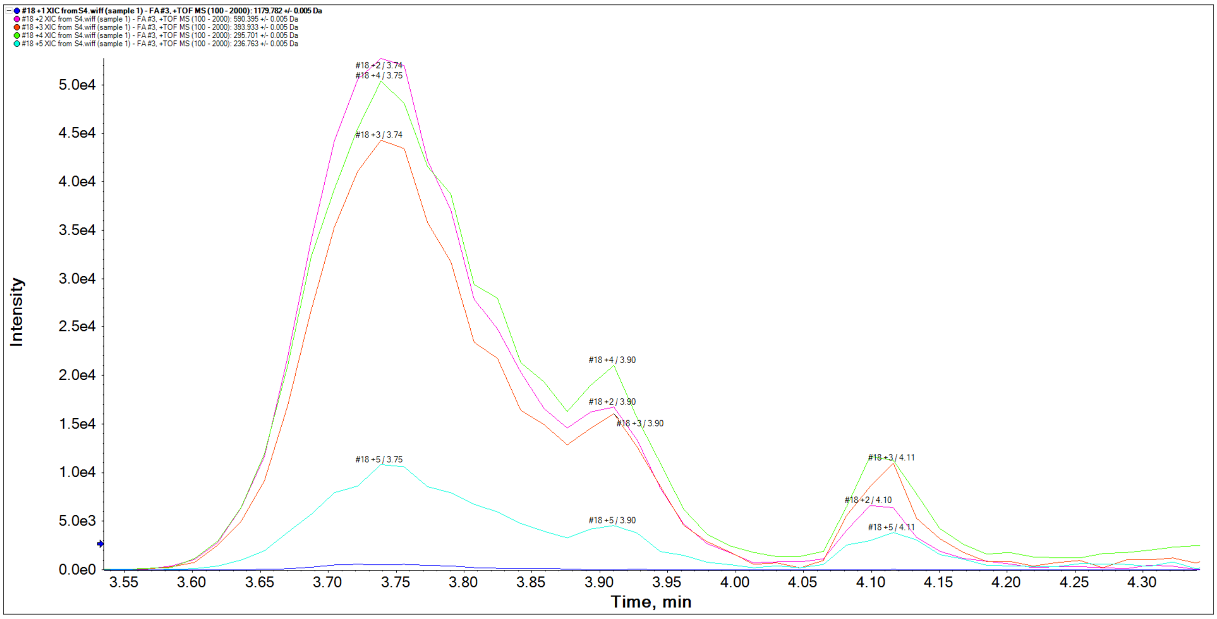

Figure S3/39. Extracted ion chromatograms (XICs) of all detected charge states of Compound #18.

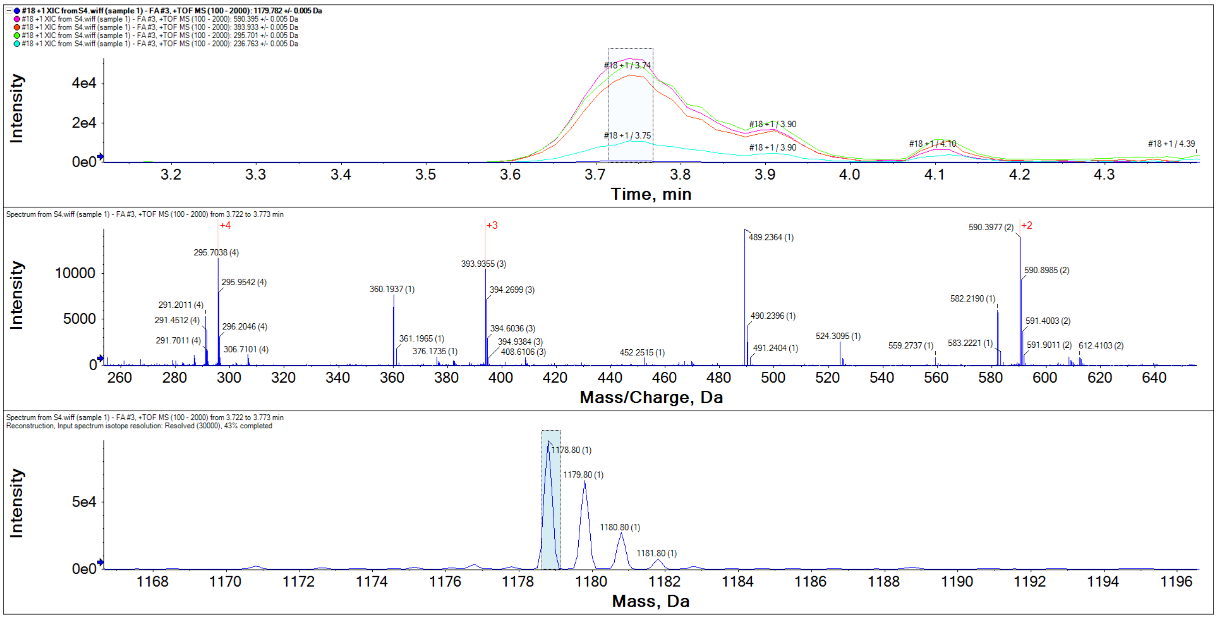

Figure S3/40. Extracted ion chromatograms (top), ESI mass spectra (middle), and reconstructed mass spectra (bottom) supporting the identification of Compound #18.

Compound #19 ($C_{59}H_{108}N_{14}O_{13}$) in induced EMC *fcl* KI CFCM (Sample #3)

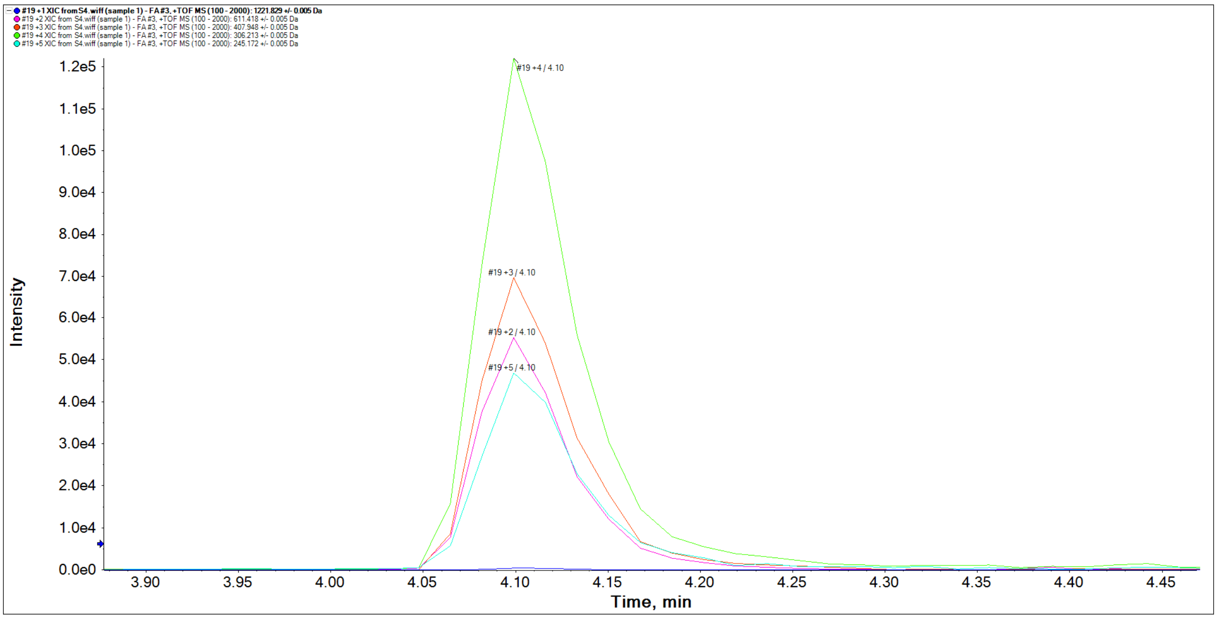

Figure S3/41. Extracted ion chromatograms (XICs) of all detected charge states of Compound #19.

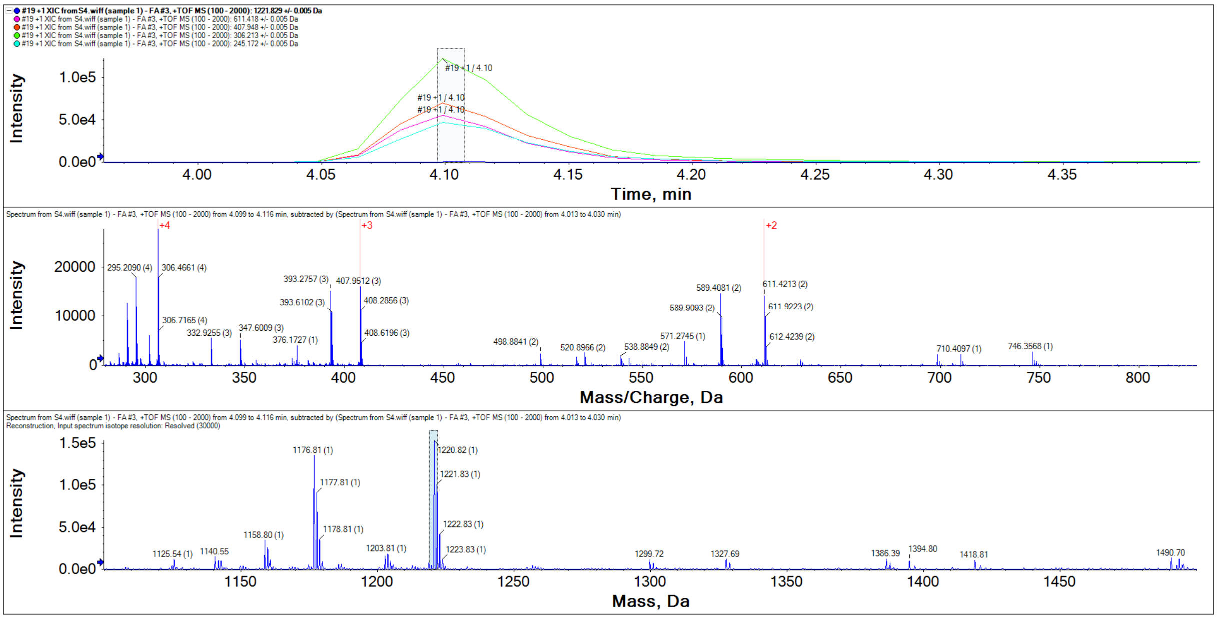

Figure S3/42. Extracted ion chromatograms (top), ESI mass spectra (middle), and reconstructed mass spectra (bottom) supporting the identification of Compound #19.

Compound #20 ($C_{60}H_{106}N_{12}O_{12}$) in induced EMC *fcl* KI CFCM (Sample #3)

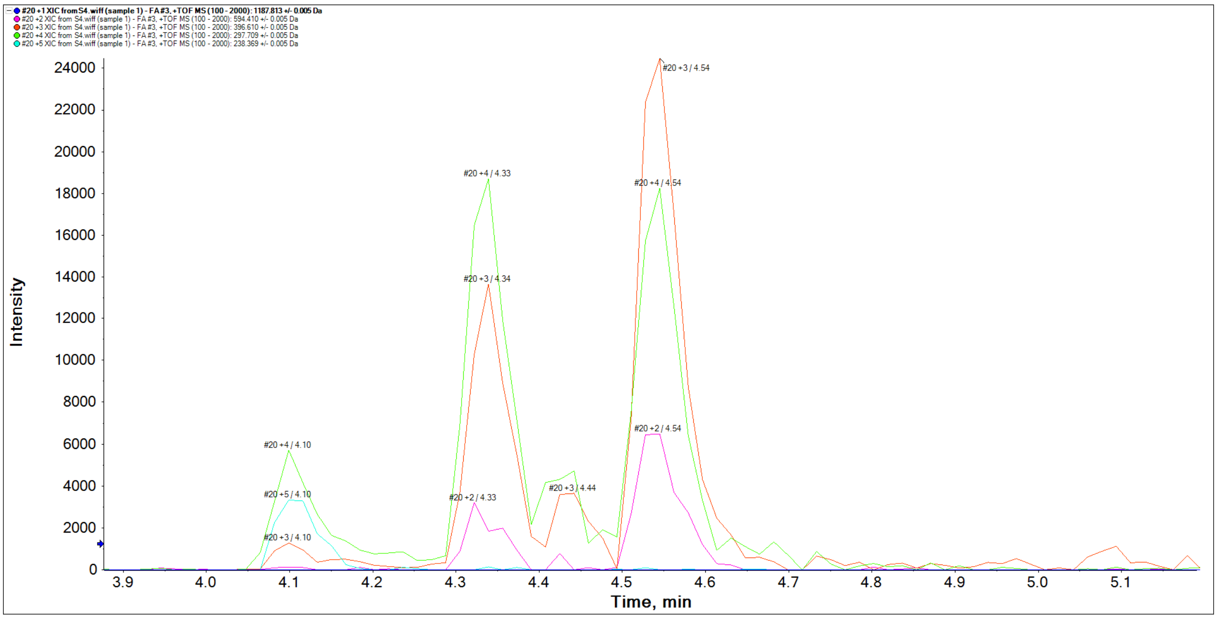

Figure S3/43. Extracted ion chromatograms (XICs) of all detected charge states of Compound #20.

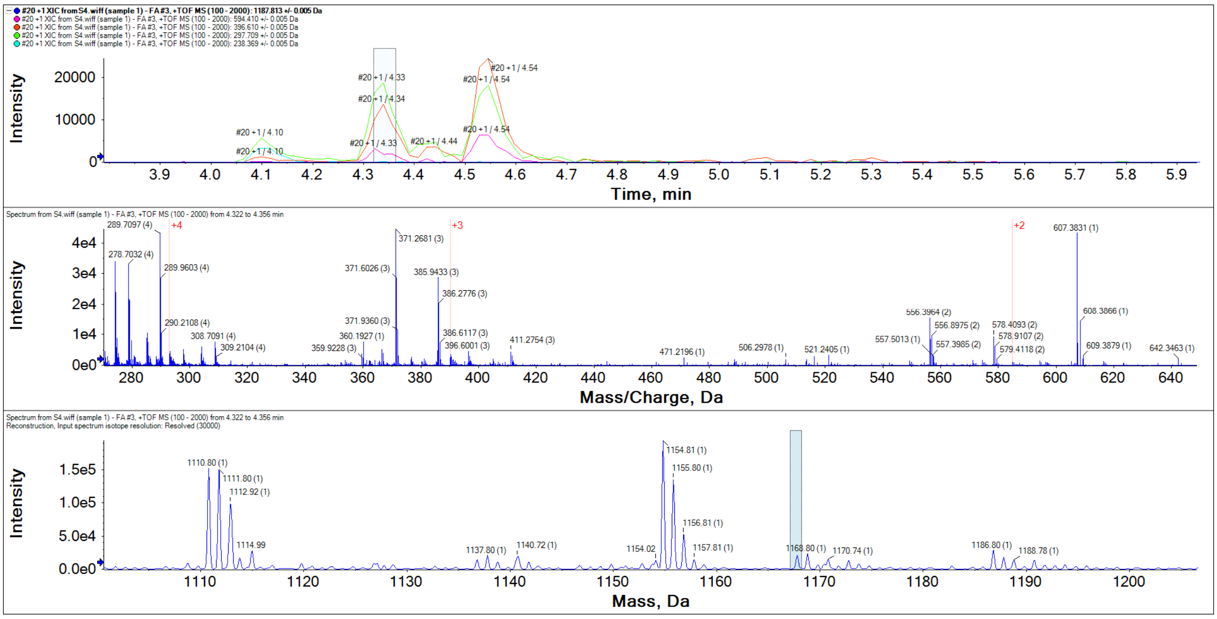

Figure S3/44. Extracted ion chromatograms (top), ESI mass spectra (middle), and reconstructed mass spectra (bottom) supporting the identification of Compound #20.

Compound #21 ($C_{59}H_{104}N_{12}O_{13}$) in induced EMC *fcl* KI CFCM (Sample #3)

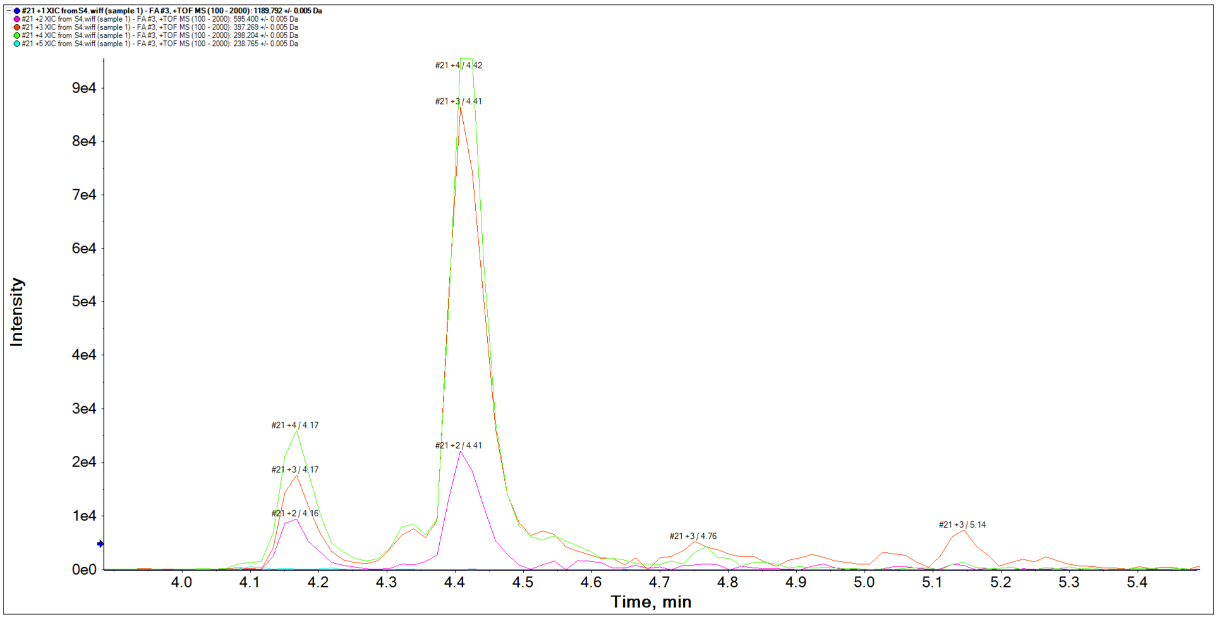

Figure S3/45. Extracted ion chromatograms (XICs) of all detected charge states of Compound #21.

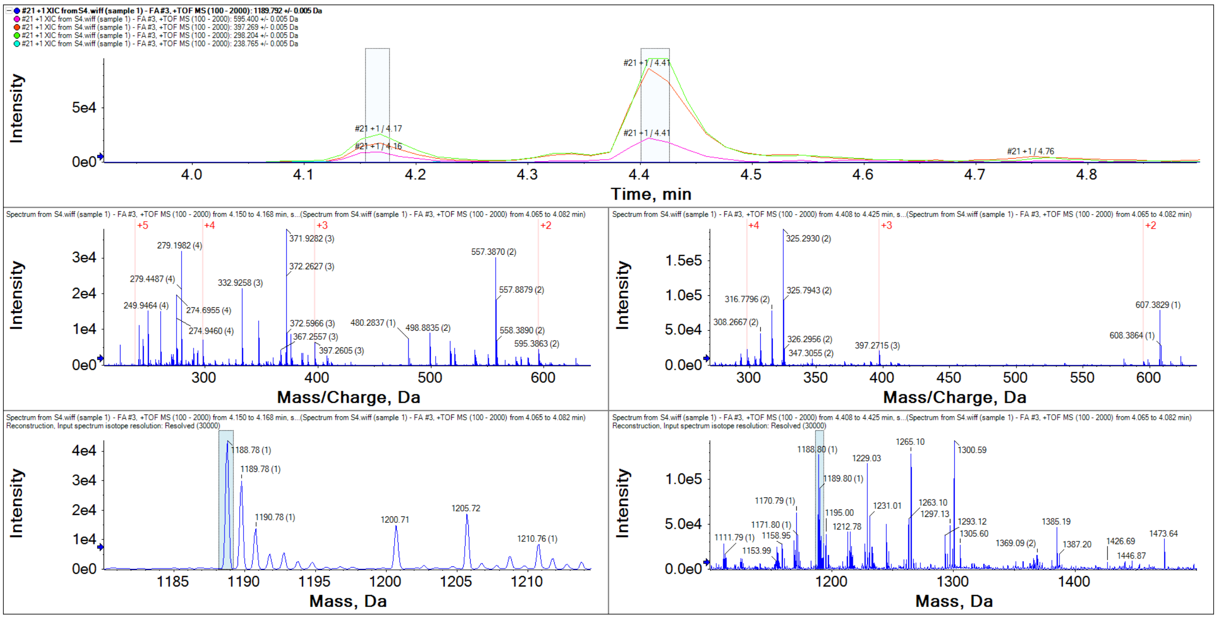

Figure S3/46. Extracted ion chromatograms (top), ESI mass spectra (middle), and reconstructed mass spectra (bottom) supporting the identification of Compound #21.

Compound #22 ($C_{62}H_{110}N_{12}O_{13}$) in induced EMC *fcl* KI CFCM (Sample #3)

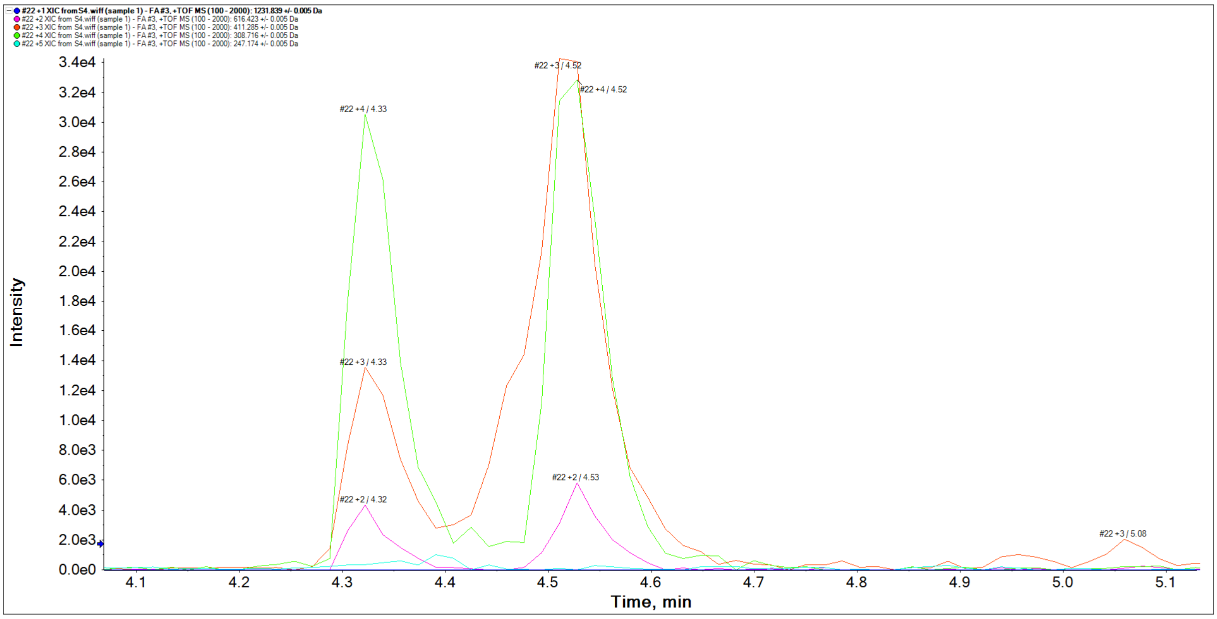

Figure S3/47. Extracted ion chromatograms (XICs) of all detected charge states of Compound #22.

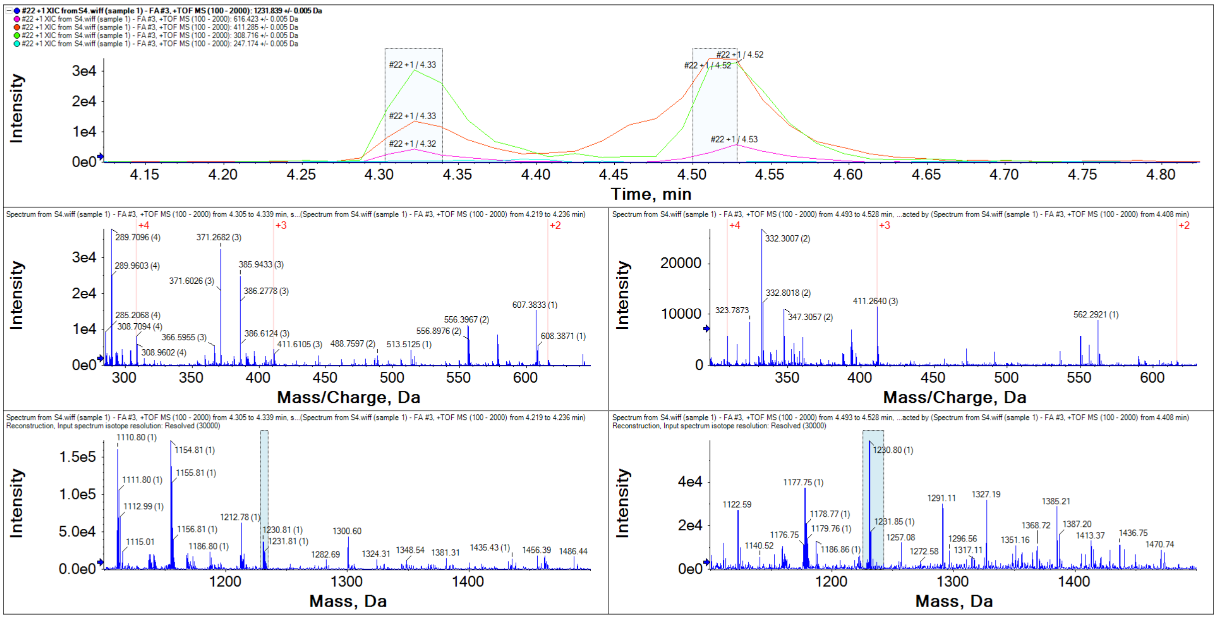

Figure S3/48. Extracted ion chromatograms (top), ESI mass spectra (middle), and reconstructed mass spectra (bottom) supporting the identification of Compound #22.
