# Supplementary material for: Anti-Fireblight Potential of Fabclavines, Synthesized by the Type Strains of Xenorhabdus szentirmaii and Xenorhabdus budapestensis
Source: Antibiotics (Basel). 2026 Sep 28;15(10):961. doi: 10.3390/antibiotics15100961 (PMC13700878; doi:10.3390/antibiotics15100961)
Supplement: Supplementary file 1 [file antibiotics-15-00961-s001.zip › Supplementary Table S1_Plasmids.pdf]

**Supplementary Table S1.** Relevant features of plasmids used in this study.

| **Name** | **Relevant features** | **References** |
|---|---|---|
| pSG76-K | R6Kγ-based $Km^R$ replicon | [65] |
| pJKI625 | A p15A-based $Km^R$ plasmid containing the P*araBAD* promoter and the *araC* gene | [64] |
| pJKI664 | A pEMBL19 derivative containing the *oriT* of RK2 | [66] |
| pBZS19 | pSG76-K derivative containing the P*araBAD* promoter and the *araC* gene | this work |
| pBZS20 | pBZS19 derivative containing the replication origin R6Kγ, *oriT* from pJKI664, P*araBAD* promoter and *araC* gene under the control of P*araC* promoter, and $Km^R$ cassette | this work |
| pBZS29 | *fcl* KI vector for EMC derived from pBZS20 | this work |
| pBZS31 | *fcl* KI vector for EMA derived from pBZS20 | this work |
