# Supplementary material for: Anti-Fireblight Potential of Fabclavines, Synthesized by the Type Strains of Xenorhabdus szentirmaii and Xenorhabdus budapestensis
Source: Antibiotics (Basel). 2026 Sep 28;15(10):961. doi: 10.3390/antibiotics15100961 (PMC13700878; doi:10.3390/antibiotics15100961)
Supplement: Supplementary file 1 [file antibiotics-15-00961-s001.zip › Supplementary Table S3_ List of strains.pdf]

Supplementary Table S3. Relevant features of bacterial strains used in this study.

Name	Relevant features	References
Xenorhabdus budapestensis (EMA) DSM16342	wt isolate, Ap^R	[38]
Xenorhabdus szentirmaii (EMC) DSM16338	wt isolate, Ap^R	[38]
Xenorhabdus budapestensis (EMA) Δ*hfq*	*hfq* deleted mutant, Ap^R	[42]
Xenorhabdus szentirmaii (EMC) Δ*hfq*	*hfq* deleted mutant, Ap^R	[42]
Xenorhabdus budapestensis (EMA) *fcl* KI	*hfq* deleted mutant, promoter of *fcl* operon exchanged to $P_{araBAD,}$ fabclavine production can be induced with d L-arabinose, Ap^R, Km^R	this work
Xenorhabdus szentirmaii (EMC) *fcl* KI	*hfq* deleted mutant, promoter of *fcl* operon exchanged to $P_{araBAD,}$ fabclavine production can be induced with L-arabinose, Ap^R, Km^R	this work
E. coli S17-1 *λpir*	S17-1 λpir, a λ lysogen derivative of S17-1 (*pro thi rec*A *hsd*R (r− m+) Tp^R Sm^R Km^S [Ω RP4-2-Tc::Mu-Km::Tn7]) expressing Π protein from *pir* gene of plasmid R6K	[44]
Erwinia amylovora strain Ea1	wt isolate, isolated from apple	[4]
Escherichia coli (E. coli ESBL)	wt isolate, extended spectrum beta-lactamase (ESBL) production, isolated from swine faeces	gifted from Á. Jerzsele
Pseudomonas aeruginosa (Pa)	wt isolate, isolated from canine external ear canal	gifted from Á. Jerzsele
Staphylococcus pseudintermedius (MRSP)	wt isolate, methicillin-resistant, isolated from canine skin	gifted from Á. Jerzsele
Enterococcus faecium ATCC6057 (*Ef*)	wt isolate	https://www.atcc.org/products/6057 gifted from Cs. Pál
