# Supplementary material for: Anti-Fireblight Potential of Fabclavines, Synthesized by the Type Strains of Xenorhabdus szentirmaii and Xenorhabdus budapestensis
Source: Antibiotics (Basel). 2026 Sep 28;15(10):961. doi: 10.3390/antibiotics15100961 (PMC13700878; doi:10.3390/antibiotics15100961)
Supplement: Supplementary file 1 [file antibiotics-15-00961-s001.zip › Supplementary Table S4_ List of primers.pdf]

Supplementary Table S4. List of primers used in this work.

Primer	sequence (5′-3′)	Reference
fabcl_EMA_Ndfor	aaacatatgtccaagacgtattttttgcatg	this work
fabcl_EMA_Prev	aactgcagctgtcttcattccactcaag	this work
fabcl_EMC_Ndfor	aaacatatgtctgagacatattttttacatgatagaaaaattcg	this work
fabcl_EMC_Prev	aactgcaggctactggcacaagcagc	this work
fabcl_EMA_test	gcaaggctgctggcac	this work
fabcl_EMC_test	ccgcccatcgctggaag	this work
hfqseqfor	tctcatgatgagatggtttatcgtg	[42]
hfqseqrev	gcacaggagaaacacctgcgg	[42]
ParaBfor	gcacggcgtcacactttgc	this work
araC_5out	taacctttcattcccagcggtc	this work
